# Novel Pyridothienopyrimidine Derivatives: Design, Synthesis and Biological Evaluation as Antimicrobial and Anticancer Agents

**DOI:** 10.3390/molecules27030803

**Published:** 2022-01-26

**Authors:** Eman M. Mohi El-Deen, Manal M. Anwar, Amina A. Abd El-Gwaad, Eman A. Karam, Mohamed K. El-Ashrey, Rafika R. Kassab

**Affiliations:** 1 Department of Therapeutic Chemistry, National Research Centre, Cairo 12622, Egypt; manal.hasan52@live.com (M.M.A.); aminanaser35@yahoo.com (A.A.A.E.-G.); 2 Department of Microbial Chemistry, National Research Centre, Cairo 12622, Egypt; eman_karam4@yahoo.com; 3 Department of Pharmaceutical Chemistry, Faculty of Pharmacy, Cairo University, Cairo 11562, Egypt; Mohamed.elashrey@pharma.cu.edu.eg; 4 Department of Chemistry, Faculty of Science (Girls), Al-Azhar University, Cairo 11754, Egypt; Rafikakassab.59@azhar.edu.eg

**Keywords:** thieno[2,3-*b*]pyridine, cyclization reactions, pyridothienopyrimidines, antimicrobial activity, HepG-2 cells, MCF-7 cells, EGFR-PK inhibition, molecular docking

## Abstract

The growing risk of antimicrobial resistance besides the continuous increase in the number of cancer patients represents a great threat to global health, which requires intensified efforts to discover new bioactive compounds to use as antimicrobial and anticancer agents. Thus, a new set of pyridothienopyrimidine derivatives **2a,b–9a,b** was synthesized via cyclization reactions of 3-amino-thieno[2,3-*b*]pyridine-2-carboxamides **1a,b** with different reagents. All new compounds were evaluated against five bacterial and five fungal strains. Many of the target compounds showed significant antimicrobial activity. In addition, the new derivatives were further subjected to cytotoxicity evaluation against HepG-2 and MCF-7 cancer cell lines. The most potent cytotoxic candidates (**3a**, **4a**, **5a**, **6b**, **8b** and **9b**) were examined as EGFR kinase inhibitors. Molecular docking study was also performed to explore the binding modes of these derivatives at the active site of EGFR-PK. Compounds **3a**, **5a** and **9b** displayed broad spectrum antimicrobial activity with MIC ranges of 4–16 µg/mL and potent cytotoxic activity with IC_50_ ranges of 1.17–2.79 µM. In addition, they provided suppressing activity against EGFR with IC_50_ ranges of 7.27–17.29 nM, higher than that of erlotinib, IC_50_ = 27.01 nM.

## 1. Introduction

In the past twenty years, the incidence of microbial infections associated with increased antimicrobial resistance has increased by alarming levels worldwide, endangering the possibility of curing many infectious diseases, and representing a global threat to population health [1,2,3]. One of the potential approaches to combat this resistance problem is based on designing innovative new molecules with different modes of action to overcome cross-resistance to present therapeutics [4,5]. Efforts in developing broad-spectrum as well as specific and targeted drugs against various microbes are continuous in all directions [5]. One of the recent strategies used for developing new antimicrobial agents is hybridization of different pharmacophores binding diverse biomolecular targets to exert synergistic effects against drug-resistant infectious diseases [6].

On the other hand, despite large advances in the diagnosis and treatment of various types of cancers, the survival of cancer patients remains poor because of the widespread side effects of anticancer therapeutics as well as the acquisition of multiple drug resistance by the cancer cells [7]. Breast and liver cancers are among the most medically significant cancers due to their high incidence and morbidity [8]. Therefore, obtaining new, effective, more selective, and less toxic anticancer agents remains one of the most urgent demands [9]. In addition, cancer patients are at higher risk of drug-resistant infectious diseases because of the weakness and immunosuppression caused by anticancer drug therapy [10], which necessitates the development of new drugs that have potent dual activity against cancer and pathogenic microbes [11].

Heterocyclic ring systems are considered key molecular structures in medicinal chemistry. In addition to their presence in the skeleton of many biological molecules such as hemoglobin, DNA, RNA, vitamins, hormones, and heterocyclic compounds [12,13,14], they produce a wide range of biological activities [15,16]. Accordingly, the structural diversity and biological prominence of heterocyclic compounds have made them attractive synthetic targets in drug design and discovery for many years [12,13]. Thieno[2,3-*b*]pyridines constitute a set of heterocyclic compounds that have beneficial effects in the treatment of many diseases. Recently, many studies have described various substituted thienopyridine compounds as significant antiproliferative agents against a wide range of human cancer cell lines [17,18,19] and as antimicrobial [20,21,22], anti-Alzheimer’s [23], anti-platelet [24], antiviral [25,26] and anti-inflammatory [27] candidates.

Furthermore, the pyrimidine structure is closely related to the nucleobases—uracil, thymine, and cytosine—which makes pyrimidine molecules important building blocks in all living cells [28,29]. Pyrimidine-based compounds exhibit a broad spectrum of pharmacological activity, such as antimalarial [30], antidiabetic [31], antimicrobial [32,33], and anticancer activities [34,35,36]. Therefore, combining thienopyridine and pyrimidine cores in the same molecular architecture, forming a pyridothienopyrimidine nucleus, serves as an attractive strategy for designing a novel scaffold with more favorable pharmacological effects [37]. Recently, various pyridothienopyrimidine derivatives were reported to produce significant antimicrobial [38,39] and anticancer activities [40,41], as well as to suppress protein kinases such as serine/threonine kinase and vascular endothelial growth factor receptor (VEGFR-2) [42,43].

**Figure 1:**
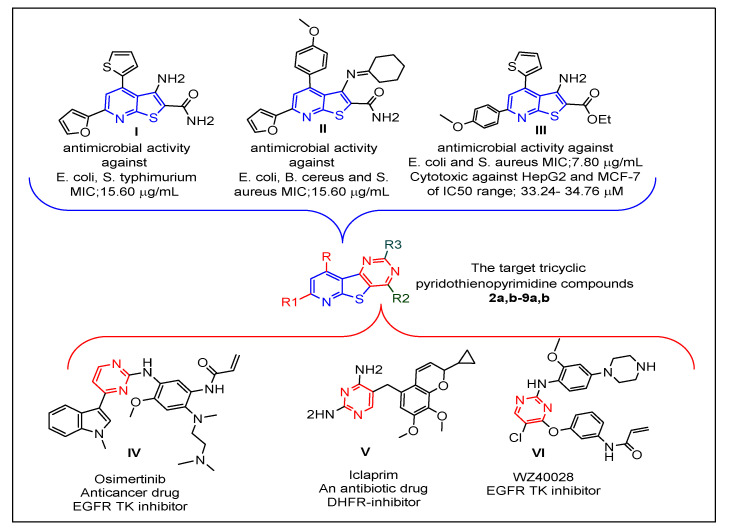
Reported thienopyridines (**I**–**III**) and pyrimidines (**IV**–**VI**) effective as antimicrobial and/or anticancer agents, and design of the new pyridothienopyrimidine compounds.

Prompted by the abovementioned issues, the strategy of this work was focused on the design of new tricyclic pyrido[3′,2′:4,5]thieno[3,2-*d*]pyrimidine compounds with structures that hybridize features of the reported thienopyridine and pyrimidine antimicrobial and/or anticancer agents [21,22,33,34] Figure 1. The resulting new pyridothienopyrimidines **2a,b–9a,b** were synthesized via different cyclization reactions of the key starting compounds, 3-aminothieno[2,3-*b*]pyridine-2-carboxamides **1a,b**. The tricyclic ring system of the target pyridothienopyridine compounds was linked at position-2 and/or position-4 to different privileged structural motifs such as morpholine, piprazine, spiro cycloalkane, oxirane, and acetamide, which are renowned for their valuable and diverse pharmacological activity [44,45,46,47,48]. All synthesized derivatives were evaluated as antimicrobial agents against a panel of Gram-positive and Gram-negative bacterial and fungal pathogens. Then, they were evaluated as cytotoxic agents against human liver carcinoma cells (HepG2) and human breast cancer cells (MCF-7) in an effort to gain new compounds possessing dual antimicrobial and anticancer activities. Since epidermal growth factor receptor (EGFR) is a key mediator in the regulation of different important cellular processes [49,50] and its overexpression displays a significant role in the development of many human cancers, including hepatic cancer and breast cancer [51,52], it was of interest to examine the EGFR inhibition activities of the compounds that revealed the most potent cytotoxic activities, as one of their cytotoxic mechanisms of action. Furthermore, molecular docking study was carried out to detect the compounds’ binding modes at the active site of EGFR-TK.

## 2. Results and Discussion

### 2.1. Chemistry

**Scheme 1:**
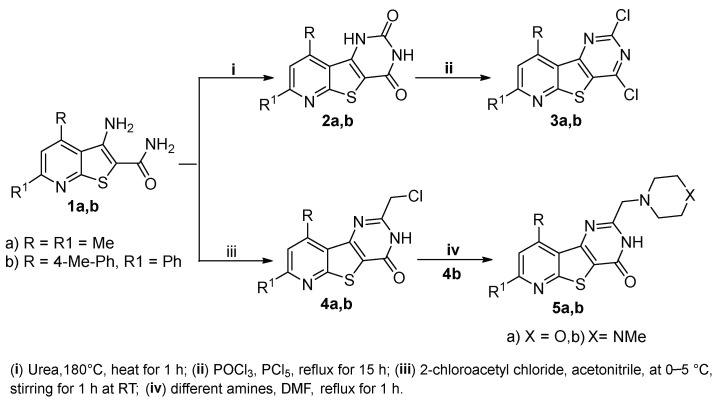
Synthesis of pyridothienopyridine derivatives **2a,b**–**5a,b**.

**Scheme 2:**
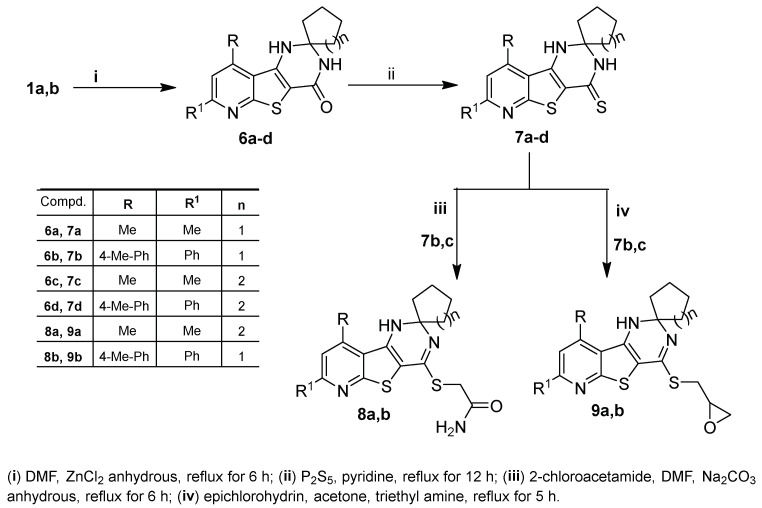
Synthesis of 1′*H*-spirocycloalkane-1,2′-pyrido[3′,2′:4,5]thieno[3,2-*d*]pyrimidine derivatives **6a–d–9a,b**.

The synthetic pathways utilized for the synthesis of the target pyridothienopyrimidine compounds **2a,b**–**9a,b** are depicted in Scheme 1 and Scheme 2. The structures of all new compounds were confirmed using ^1^H-NMR, ^13^C-NMR (Supplementary Materials), IR, and mass spectral data alongside the elemental microanalyses. Heating 3-amino-4,6-dimethyl/6-phenyl-4-(*p*-tolyl)thieno[2,3-*b*]pyridine-2-carboxamides **1a,b**, the starting compounds, with urea at 180 °C for 1 h led to the formation of the corresponding pyridothienopyrimidine-2,4-diones **2a,b,** which were further refluxed with a mixture of phosphorus oxychloride and phosphorus pentachloride to give the corresponding 2,4-dichloro derivatives **3a,b**, respectively. On the other hand, the treatment of the starting compounds **1a,b** with 2-chloroacetyl chloride in cold acetonitrile resulted in cyclization via substitution reaction followed by intramolecular elimination to give the 2-(chloromethyl)-pyridothienopyrimidin-4(3*H*)-ones **4a,b**, respectively. The subsequent treatment of **4b** with different amines, namely, morpholine and 1-methylpiperazine, resulted in the corresponding 2-(morpholinomethyl)-/2-((4-methylpiperazin-1-yl)methyl)-pyridothieopyrimidin-4(3*H*)-ones **5a,b** (Scheme 1). IR spectra of compounds **2a,b** found two bands in the region 3394–3112 cm^−1^ ascribed to 2NH and two bands in the region 1728–1640 cm^−1^ related to the 2C=O groups of the pyrimidine-2,4(1*H*,3*H*)-dione ring. By comparison, IR spectra of the 2,4-dichloro derivatives **3a,b** revealed the absence of the previous bands; instead, they showed two bands in the region 825–768 cm^−1^ referring to the newly formed C-Cl groups. In addition, ^1^H-NMR spectra of compounds **2a,b** revealed the two 2NH groups to be two D_2_O exchangeable singlets at δ 10.42–11.71 ppm, which vanished in the ^1^H-NMR spectra of 2,4-dichloro derivatives **3a,b**. Furthermore, the 2CH_3_ of **2a**, **3a** and CH_3_ of the p-tolyl moiety of **2b**, **3b** exhibited as singlet signals in the range 2.43–2.79 ppm along with aromatic protons in the range δ 7.21–8.24 ppm. The ^13^C NMR spectra of compounds **2a,b** and **3a,b** revealed CH_3_ moieties at δ 20.40–24.36 ppm in addition to the aromatic carbons.

IR spectra of compounds **4a,b**, **5a,b** represented NH and C=O groups as absorption bands in the regions 3440–3387 cm^−1^ and 1669–1655 cm^−1^, respectively, while the C-Cl band of **4a** appeared at 772 cm^−1^ and that of **4b** was at 776 cm^−1^. Furthermore, ^1^H-NMR spectra of **4a,b** and **5a,b** exhibited, in addition to the signals of the parent protons, a singlet signal in the region δ 3.29–4.66, referring to the methylene protons of the -NCH_2_ moiety. Additionally, the eight protons of the morpholine moiety of **5a** appeared as two singlets at δ 2.38 and 3.54 ppm, while those of the piperazine ring of **5b** appeared as multiplet signals in the range δ 2.81–3.01 ppm alongside a singlet signal at δ 2.54 ppm due to its -N-CH_3_ group. The ^13^C-NMR spectra of **5a** exhibited the 2CH_2_N- and 2CH_2_O of the morpholine moiety as two signals at δ 53.37 and 60.65 ppm, respectively.

The synthesis of spiro cycloalkane-pyridothienopyrimidin-4′-ones **6a–d** in good yields was achieved via cyclocondensation reactions of the starting **1a,b** with the appropriate cyclic ketones, cyclopentanone and/or cyclohexanone, in DMF containing zinc chloride anhydrous. Treating the latter compounds (**6a–d)** with phosphorus pentasulfide in refluxing pyridine yielded the corresponding 1′*H*-spiro[cycloalkane-1,2′-pyridothienopyrimidine]-4′(3′*H*)-thiones **7a–d** (Scheme 2). IR spectra of **6a–d** and **7a–d** revealed the presence of different absorption bands in the regions 3427–3157 cm^−1^ related to 2NH groups, 1660–1644 cm^−1^ related to the C=O groups of **6a-d** and 1247–1232 cm^−1^ related to the C=S groups of compounds **7a–d**. ^1^H-NMR spectra of **6a–d** and **7a–d** represented the eight protons of the spiro-cyclopentane and the ten protons of the spiro-cyclohexane substituents as multiplet signals in the range δ 1.21–2.11 ppm alongside one D_2_O exchangeable singlet in the range δ 4.59–6.49 ppm, corresponding to the NH at position-1, and another D_2_O exchangeable singlet appeared downfield in the range δ 7.81–9.85 ppm, related to the NH group at position-3. ^13^C-NMR spectra of **6a–d** and **7a–d** revealed various singlets in the ranges δ 19.26–38.33 ppm assignable to the cyclopentane and cyclohexane carbons, δ 69.49–70.81 ppm related to the spiro carbons, δ 164.40–167.80 ppm assigned to the C=O groups of **6a–d** and δ 181.33–183.35 ppm ascribed to C=S moieties of compounds **7a–d**, as well as the signals of the parent carbons, which appeared in their correct regions.

Compounds **7c,b** were treated with 2-chloroacetamide in refluxing DMF containing sodium carbonate anhydrous to obtain the corresponding 4-sulfanyl acetamide derivatives **8a,b**, while the treatment of **7c,b** with epichlorohydrin in refluxing acetone containing a catalytic amount of triethyl amine resulted in the formation of the corresponding 4-(oxiran-2-ylmethy)sulfanyl derivatives **9a,b** (Scheme 2). The S-alkylation at position-4 was supported by ^1^H NMR and ^13^C-NMR spectra of **8a,b** and **9a,b** due to the vanishing of both the signal related to one of the two NH protons and that of the C=S carbon. Moreover, the ^1^H NMR spectra of compounds **8a,b** exhibited singlet signals at δ 4.05 and 4.19 ppm due to SCH_2_ protons of the newly formed acetamide side chain. ^1^H NMR spectra of compounds **9a,b** represented the protons of the oxirane ring as two multiplets in the range δ 2.86–2.94 ppm, related to the methylene protons, and a third multiplet at δ 3.72–3.95 ppm, due to the methine proton, while the methylene protons of SCH_2_ appeared as a doublet signal at δ 3.51 and 3.67 ppm. Additionally, the ^13^C-NMR spectra **9a,b** showed three signals at the range δ 31.88–53.45 ppm referred to SCH_2_, OCH_2_, and OCH moieties.

Confirmation of the molecular structures of the new compounds was also supported by their mass spectra, which represented the correct molecular ion peaks.

### 2.2. Biological Activity

#### 2.2.1. Antimicrobial Activity

**Figure 2:**
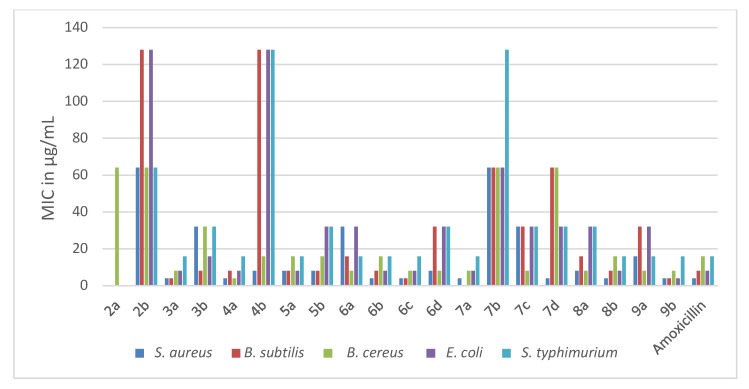
Antibacterial activities (MIC in µg/mL) of the new pyridothienpyrimidine compounds **2a,b–9a,b** and the reference antibiotic (amoxicillin trihydrate).

**Figure 3:**
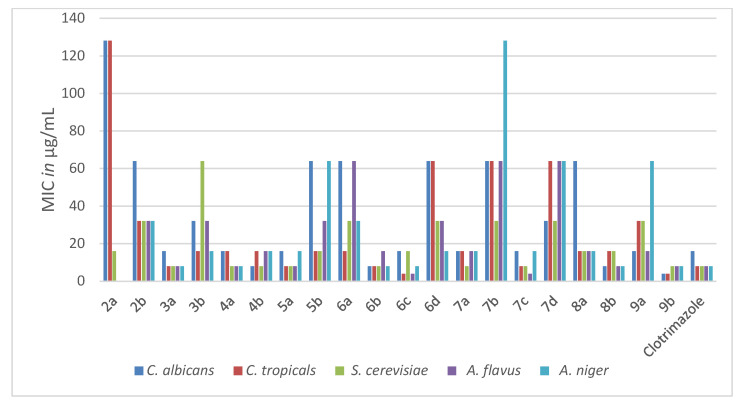
The antifungal activities (MIC in µg/mL) of the new pyridothienpyrimidine compounds **2a,b–9a,b** and the reference drug (clotrimazole).

The newly synthesized pyridothienopyrimidine componds **2a,b–9a,b** were investigated for their antimicrobial activities against a panel of microbial strains, three Gram-positive bacteria *viz. Staphylococcus aureus 25923*, *Bacillus subtilis 6633*, *Bacillus cereus 33018*, two Gram-negative bacteria *viz. Escherichia coli 8739*, *Salmonella typhimurium 14028*, three yeasts *viz*. *Candida albicans 10231*, *Candida tropicals 750*, *Saccharomycese cerevisiae* and two fungi *viz. Aspergillus flavus*, *Aspergillus niger EM77 (KF774181)*. Their activities were expressed in terms of minimal inhibitory concentration (MIC) (μg/mL). Additionally, amoxicillin trihydrate and clotrimazole were utilized as positive antibiotic and antifungal controls. The obtained MIC results are represented in Table 1 and Table 2 and Figure 2 and Figure 3.

Based on the MIC values in Table 1, the antibacterial activity of the target compounds **2a,b**–**9a,b** was compared with that of amoxicillin, whose MICs ranged between 4–16 μg/mL against the five bacterial strains. It is evident that pyridothienopyrimidine-2,4(1*H*,3*H*)-dione derivatives **2a,b** showed antibacterial activity varying from weak to inactive against the tested strains, with MICs ranging from 64 to >128 μg/mL. However, the conversion of **2a,b** to 2,4-dichloro derivatives **3a,b** led to a significant increase in antibacterial activity, especially for 7,9-dimethyl derivative **3a**, which exhibited more potent activity than amoxicillin against *B*. *subtilis* and *B. cereus* and equalized with it against the other strains. In addition, 2-chloromethyl derivatives **4a,b** revealed significant antibacterial activity, particularly 7,9-dimethyl derivative **4a**, which showed the most potent activity against *B. cereus* with MIC = 4 μg/mL and had equipotent activity to amoxicillin against the other strains. While 7-Phenyl-9-(p-tolyl) analogue **4b** revealed potent activity against *S. aureus* and *B*. *cereus*, it showed weak activity against other bacterial strains with MIC = 128 μg/mL. Further reaction of 2-chloromethyl derivative **4b** with amines led to enhanced antibacterial activity, where *2*-morpholinomethyl derivative **5a** showed potent activity equal to amoxicillin against all the tested bacterial strains except *S. aureus,* with MICs ranging from 8 to 16 μg/mL. In addition, 2-(4-methylpiperazin-1-yl) derivative **5b** revealed increasing activity against *B. subtilis, E. coli* and *S.* *typhimurium***.**

Spiro cycloalkane-1,2′-pyridothienopyrimidin]-4′(3′*H*)-ones **6a–d** exhibited antibacterial activity varying from potent to moderate against the tested strains, with MICs ranging from 4 to 32 μg/mL. Compound **6b** showed an activity equal to that of the reference drug against the five bacterial strains, while **6c** exceeded the potency of amoxicillin against *B*. *subtilis* and *B. cereus* and gave equipotent activity against the other strains. The other derivatives, **6a** and **6d,** showed activity varying from potent to moderate with MICs ranging 8–32 μg/mL. The conversion of **6a–d** to 4′(3′*H*)-thiones analogues **7a–d** resulted in an obvious weakening in the activity against the most of the tested strains, particularly derivative **7b**, which showed weak activity against the five strains with MICs in the range 64–128 μg/mL. However, the S-alkylation of **7b** and **7c** at position-4 afforded increases in the antibacterial activity. The spiro cyclopentane 4-sulfanylacetamide derivative **8b** revealed potent activity similar to that of amoxicillin, and the spiro cyclohexane analogue **8a** showed potent to moderate activity with MICs ranging from 8 to 32 μg/mL. Spiro cyclopentane 4-(oxiran-2-ylmethyl)sulfanyl derivative **9b** showed the most potent antibacterial activity against all tested strains, especially against *E. coli*, while spiro cyclohexane analogue **9a** showed antibacterial activity ranging from potent to moderate with MICs in the range 8–32 μg/mL.

The antifungal activity of the new compounds **2a,b**–**9a,b** was evaluated according to their MIC values in comparison to the MIC values of the reference antifungal drug clotrimazole, listed in Table 2. The target compounds (**3a**, **4a**, **4b**, **5a**, **6b**, **6c**, **7a**, **7c**, **8b** and **9b**) appeared to be potent antifungal candidates, representing MIC values ranging from 4 to 16 μg/mL against all the tested yeasts and fungi strains, similar to the range of clotrimazole (MICs; 4–16 μg/mL). Furthermore, 4-(oxiran-2-ylmethyl)sulfanyl derivative **9b** represented more potent antifungal activity than that of clotrimazole against the yeast pathogens *C. albicans* and *C. tropicals*, with MICs of 4 and 4 μg/mL, respectively. The rest of the target compounds displayed moderate to weak activity against the tested yeasts and fungi strains. The obtained results suggested that the conjugation of chloroine, chloromethyl, morpholinomethyl and spiro cyclopentane/cyclohexane at position-2 of the parent pyridothienopyrimid-4(3*H*)-one (**3a**, **4a,b**, **5a**, **6b,c**, **7c**) as well as the attachment of (oxiran-2-ylmethyl)sulfanyl and/or sulfanylacetamide side chains at position-4 of the pyridothienopyrimidine scaffold (**8b**, **9b**) produced a beneficial impact for antimicrobial activity, resulting in promising broad-spectrum growth inhibition activity against the examined Gram-positive and Gram-negative microbes as well as fungal pathogens.

#### 2.2.2. Cytotoxic Activity

The newly synthesized pyridothienopyrimidine derivatives **2a,b**–**9a,b** were subjected to antiproliferative activity evaluation against two cancer cell lines, hepatocellular carcinoma (HepG2) and breast cancer (MCF-7), by using the MTT colorimetric assay[53]. The cytotoxic activity of the target compounds was compared with that of doxorubicin, utilized as a positive control. The concentrations of the examined derivatives that induced 50% inhibition of cell viability (IC_50_, μM) were detected and are listed in Table 3.

**Figure 4:**
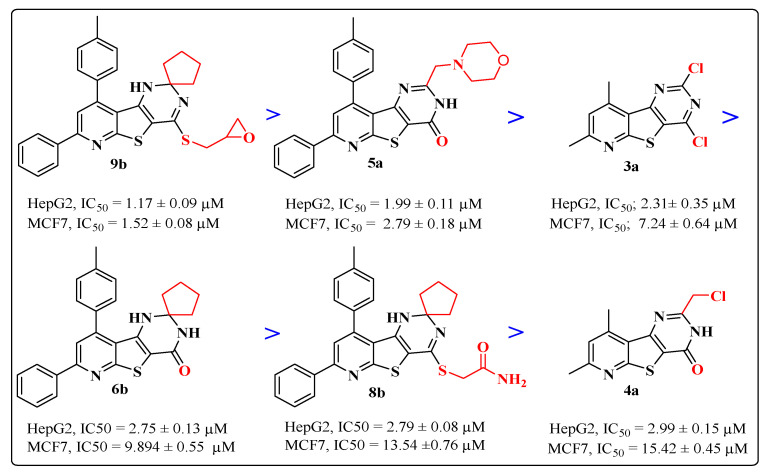
The cytotoxic activity of the most potent compounds.

Based on IC_50_ values from Table 3, the examined compounds displayed versatile anti-proliferative activities against the tested cell lines, producing more potent growth inhibitory activity against HepG2 cells than MCF-7 cells with IC_50_ ranges of 1.17–56.18 µM and 1.52–77.41μM, respectively, compared to doxorubicin’s IC_50_ values of 2.85 and 3.58 µM, respectively. The most active cytotoxic activity was exhibited by the target compounds (**9b**, **5a**, **3a**, **6b**, **8b** and **4a**), listed in descending order according to their activity against the two cell lines, as represented in Figure 4. Interestingly, the attachment of the sulfanylmethyl-oxirane side chain at position-4 of the spiro cyclopenane-1,2′-pyridothienopyrimidine nucleus in compound **9b** produced the most potent growth inhibition activity against both HepG2 and MCF-7 cancer cells, approximately 2–3 fold higher than doxorubicin, representing IC_50_ values of 1.17 and 1.52 μM, respectively. However, the activity slightly decreased against HepG2 cells and detectably decreased against MCF-7 for the spiro cyclohexane sulfanylmethyl oxirane analogue **9a** compared to doxorubicin, with IC_50_ values of 4.88 and 23.56 µM, respectively. Furthermore, the incorporation of the morpholine nucleus into the pyridothienopyrimidine scaffold at position-2 in compound **5a** led to a significant cytotoxic potency against both HepG2 and MCF-7, greater than that obtained from the reference drug, at IC_50_ values of 1.99 and 2.79 µM, respectively. However, the 4-methylpiperazinyl analogue **5b** exhibited an observable reduction in growth inhibition activity towards both tested cancer cell lines with IC_50_ values of 10.16 and 21.06 µM, respectively. A comparable growth inhibitory activity to doxorubicin was displayed against HepG2 cancer cells by the 2,4-dichloro-pyridothienopyrimidine derivative **3a** at an IC_50_ value of 2.31 μM, but its activity was less against MCF-7 with an IC_50_ value of 7.24 µM, while its 7-phenyl-9-*p*-tolyl analogue **3b** represented a detectable drop in potency, with IC_50_ values of 11.34 and 24.72 µM against HepG2 and MCF-7, respectively. The spirocyclopentane-pyridothienopyrimidin-4-one derivative **6b** was nearly equipotent to doxorubicin in inhibiting the growth of HepG2 cancer cells, with an IC_50_ value of 2.75 µM, but produced a mild decrease in activity against MCF-7 with IC_50_ of 9.89 µM; however, the displacement of the spiro pentane moiety with spirocyclohexane in the analogue **6d** led to a twofold decrease in cytotoxic activity against HepG2 cells and a significant drop in potency against MCF-7 cancer cells with IC_50_ values of 4.45 and 21.67 µM, respectively. The other members in this series, **6a,c**, appeared to be significantly less potent candidates than the reference drug, with IC_50_ ranges of 12.11–77.41 µM.

Additionally, the spiro cyclopentane sulfanylacetamide **8b** produced an equivalent cytotoxic potency to that obtained by doxorubicin against HepG2 cells, with an IC_50_ value of 2.79 µM, but less potency than doxorubicin against MCF7, with an IC_50_ value of 13.54 µM. The spiro cyclohexane sulfanylacetamide analogue **8a** appeared to be a less potent growth inhibitor towards both HepG2 and MCF-7 cancer cells, with IC_50_ values of 6.78 and 20.88 µM, respectively. Finally, the 2-chloromethyl-7,9-dimethyl derivative **4a**, the last among the most potent compounds, showed significant growth inhibitory activity against HepG2 cancer cells with IC_50_ = 2.99 µM, but its potency decreased against MCF-7 cells with an IC_50_ value of 15.42 µM, whereas a high drop in activity was shown by the 7-phenyl-9-p-tolyl analogue **4b** towards the two cancer cell lines with IC_50_ values of 36.52 and 43.27 µM, respectively. The rest of the target compounds, the spiro[cycloalkane-1,2′-pyridothienopyrimidine-4′(3′*H*)-thiones **7a–d** and the pyridothienopyrimidine-2,4(1*H*,3*H*)-diones **2a,b**, showed moderate to weak cytotoxic activities in the IC_50_ range of 10.35–41.62 µM, compared to doxorubicin.

The frequency and severity of undesirable effects on normal cells at therapeutic doses are considered among the most important characteristics that differentiate anticancer drugs from each other. Accordingly, the cytotoxic activity of the most active compounds (**3a, 4a, 5a, 6b, 8b, 9b**) was evaluated against the normal WISH cell line (Human amnion normal Liver cells) via MTT assay as IC_50_ values in μM, listed in Table 3. The obtained results revealed that the tested compounds had IC_50_ values in the range 394.98–460.23 µM, nearly equal to that obtained by the reference drug doxorubicin, IC_50 doxorubicin_ 432.10 µM, which confirmed the high safety of the most potent compounds towards normal cells.

#### 2.2.3. In Vitro EGFR Enzyme Inhibition Assay

The most potent cytotoxic compounds (**3a, 4a, 5a, 6b, 8b, 9b**) were subjected to further investigations of their inhibiting profiles against EGFR, in order to study one of their proposed modes of action as potent cytotoxic agents [54]. Accordingly, these derivatives were assessed as EGFR kinase inhibitors, using erlotinib as a reference drug as it is one of the most potent EGFR inhibitors [55]. The obtained results were expressed as IC_50_ values (nM) (Table 4). Interestingly, the most cytotoxic derivatives, spiro cyclopentane-1,2′ pyridothienopyrimidine–oxirane **9b** and pyridothienopyrimidine–morpholine **5a**, appeared to be 3–2 times more potent than erlotinib, representing IC_50_ values of 7.27, 9.66 and 27.01 nM, respectively. The 2-chloromethyl-pyridothienopyrimidin-4-one derivative **4a** displayed a slight decrease in inhibition activity, but remained 1.5-fold more potent than erlotinib. An evident drop in EGFR inhibition activity was observed for the rest of the examined derivatives (**3a**, **6b**, **8b**) representing an IC_50_ range of 38.44–53.57 nM.

### 2.3. Molecular Docking Studies

**Figure 5:**
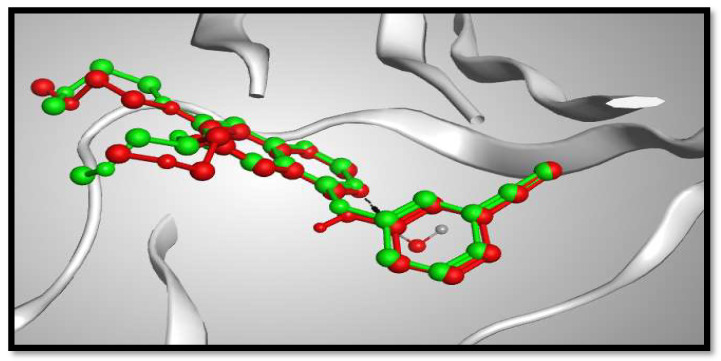
3D representation of the superimposition of the co-crystallized ligand (purple) and the docking pose (dark grey) of ERL at the active site of the EGFR enzyme.

Molecular docking studies were performed to study the binding modes of the most potent cytotoxic compounds (**3a, 4a, 5a, 6b, 8b, 9b**) to the active sites of the target EGFR compared with erlotinib (ERL) as EGFR inhibitor. Docking setup was first validated through self-docking of the co-crystallized ligand (ERL) in the vicinity of the binding site of the enzyme. The docking score (S) was –10.48 kcal/mol. and root mean square deviation (RMSD) was 1.03 Å (Figure 5). The calculated RMSD value confirms the validity of the docking procedure.

**Figure 6:**
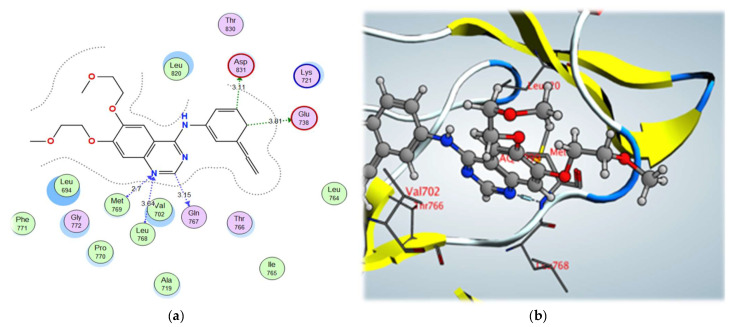
(**a**) 2D interactions and (**b**) 3D interactions of erlotinib within the EGFR kinase domain’s active site.

Examination of the binding interactions of erlotinib to the active site of the EGFR kinase domain showed several conventional hydrogen bond interactions with the Met769, Leu768, Val702 and Leu820 amino acids (Figure 6).

**Figure 7:**
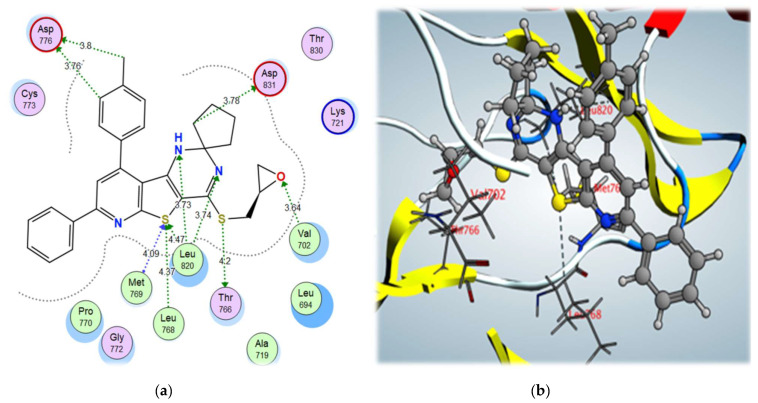
(**a**) 2D interactions and (**b**) 3D interactions of compound **9b** within the EGFR kinase domain’s active site.

From the docking results of the examined compounds (Table 5), it was noticed that all compounds showed binding interactions within the active site of EGFR kinase domain with binding scores ranging from –12.01 to –8.94 kcal/mol. The spiro cyclopentane 4-((oxiran-2-ylmethyl)sulfanyl derivative **9b**, which showed the highest biological activity, also showed the highest binding score of –12.01 kcal/mol, through binding with the key amino acids, Met769, Leu768, and Leu820, via the S atom of thiophene ring using a hydrogen bond acceptor, with further interactions with Thr766 via σ-hole bonding with the S atom of the S-methyl side chain. In addition, it bound to the amino acid Val702 through a hydrogen bond acceptor with the O atom of the oxirane moiety and showed high fitting in the vicinity of the active site, contributing to its high biological activity (Figure 7).

**Figure 8:**
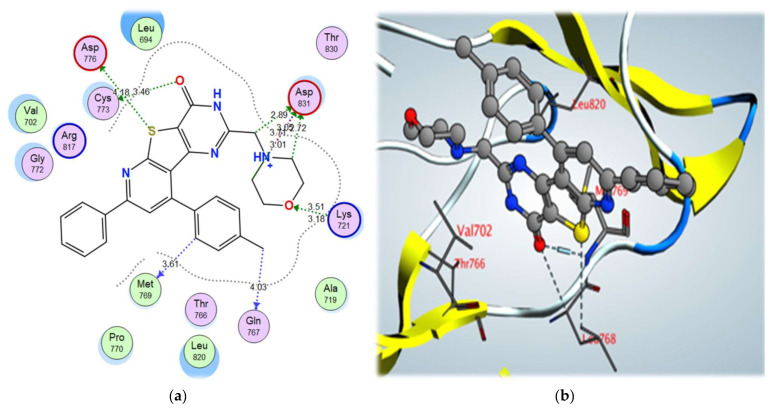
(**a**) 2D interactions and (**b**) 3D interactions of compound **5a** within the EGFR kinase domain’s active site.

**Figure 9:**
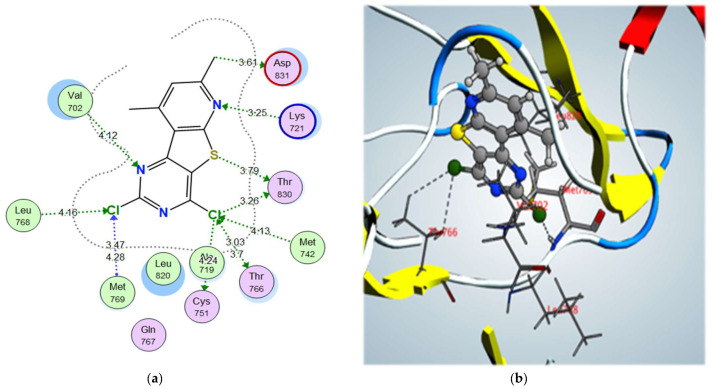
(**a**) 2D interactions and (**b**) 3D interactions of compound **3a** within the EGFR kinase domain’s active site.

*2*-morpholinomethyl derivative **5a** and 2,4-dichloro derivative **3a** also showed higher binding scores than the co-crystallized ligand, erlotinib, at –11.48 and –11.42 kcal/mol, respectively; they showed a good binding pattern with the key amino acids, and compound **5a** showed an ionic interaction with Asp831 revealing its high biological activity (Figure 8 and Figure 9). Other tested derivatives showed a lower binding score than erlotinib, with less binding interaction with the key amino acids.

## 3. Materials and Methods

### 3.1. Chemistry

#### 3.1.1. General Information

The melting points were obtained in open capillary tubes using an Electrothermal IA9100 digital melting point apparatus. Elemental microanalyses were carried out at the Micro Analytical Unit at Cairo University. ^1^H NMR and ^13^C NMR spectra were recorded on a Bruker High Performance Digital FT-NMR Spectrometer Advance III (400/100 MHz) in the presence of TMS as the internal standard at Ain Shams University, Cairo, Egypt. Infrared spectra were measured using the KBr disc technique on a Jasco FT/IR-6100 Fourier transform IR spectrometer (Japan) at the scale of 400–4000 cm^−1^. ESI-mass spectra were determined using an Advion Compact Mass Spectrometer (CMS), NY, USA. TLC on silica gel-precoated aluminum sheets (Type 60, F 254, Merck, Darmstadt, Germany) was used for following up the reactions and checking the purity of the chemical compounds using chloroform/methanol (3:1, *v*/*v*), and spots were detected with iodine vapor or through exposure to a UV lamp at δ 254 nm for several seconds. The nomenclature of the compounds is according to the IUPAC system. The starting compounds, 3-amino-thieno[2,3-*b*]pyridine-2-carboxamides (**1a,b**), were prepared using the reported method [56].

#### 3.1.2. Synthesis of Pyrido[3′,2′:4,5]thieno[3,2-d]pyrimidine-2,4(1H,3H)-diones 2a,b

A mixture of compounds 1a,b (5 mmol) and urea (0.42 g, 7 mmol) was heated at 180°C for 1 h. The solidified residue was treated with hot water and the obtained solid was separated by filtration. The solid was washed with hot water several times and recrystallized from acetone to yield compounds **2a,b.**

*7,9-Dimethylpyrido[3′,2′:4,5]thieno[3,2-d]pyrimidine-2,4(1H,3H)-dione* (**2a**) was obtained from **1a** (1.11 g, 5 mmol) in 85% yield (1.05 g) as a brown solid, m.p. 325 °C. IR (KBr, υ_max_/cm^−1^): 3394, 3112 (2NH), 3010 (CH-aromatic), 2828 (CH-aliphatic), 1728, 1662 (2C=O); ^1^H-NMR (DMSO-d6, 400 MHz): δ = 2.55 (s, 3H, CH_3_), 2.79 (s, 3H, CH_3_), 7.21 (s, 1H, Ar-H), 10.59, 11.65 (2s, 2H, 2NH, D_2_O exchangeable); ^13^C-NMR (DMSO-d6, 100 MHz): δ = 20.40 (CH_3_), 24.36 (CH_3_), 108.64, 120.72, 122.85, 140.22, 145.63, 152.01, 160.07, 160.41, 161.48 (Ar-C, 2 C=O); ESI-MS: *m*/*z* = 247.33 [M − H^+^]. Anal. Calcd. for C_11_H_9_N_3_O_2_S (247.27): C, 53.43; H, 3.67; N, 16.99; S, 12.97% Found: C, 53.12, H, 3.91; N, 16.62; S, 13.31%.

*7-Phenyl-9-(p-tolyl)pyrido[3′,2′:4,5]thieno[3,2-d]pyrimidine-2,4(1H,3H)-dione* (**2b**) was obtained from **1b** (1.79 g, 5 mmol) in 83% yield (1.60 g) as an orange solid, m.p. 316–317 °C. IR (KBr, υ_max_/cm^−1^): 3370, 3154 (2NH), 3024 (CH-aromatic), 2925, 2855 (CH-aliphatic), 1702, 1640 (2C=O); ^1^H-NMR (DMSO-d6, 400 MHz): δ = 2.43 (s, 3H, CH_3_), 7.46–7.52 (m, 5H, Ar-H), 7.60 (d, 2H, *J* = 8 Hz, Ar-H), 7.96 (s, 1H, Ar-H), 8.24 (d, 2H, *J* = 5.2 Hz, Ar-H), 10.42, 11.71 (2s, 2H, 2NH, D_2_O exchangeable); ^13^C-NMR (DMSO-d6, 100 MHz): δ = 21.42 (CH_3_), 112.45, 115.62, 118.27, 121.46, 127.21, 127.87, 129.30, 129.69, 130.23, 133.99, 138.39, 139.64, 144.46, 149.89, 151.96, 156.07, 157.40, 160.46 (Ar-C, 2 C=O); ESI-MS: *m*/*z* = 384.39 [M − H^+^]. Anal. Calcd. for C_22_H_15_N_3_O_2_S (385.44): C, 68.56; H, 3.92; N, 10.90; S, 8.32% Found: C, 68.74, H, 4.21; N, 10.68; S, 8.59%.

#### 3.1.3. Synthesis of 2,4-Dichloropyrido[3′,2′:4,5]thieno[3,2-*d*]pyrimidines **3a,b**

To a solution of compounds **2a,b** (1 mmol) in phosphorus oxychloride (20 mL), phosphorus pentachloride (0.41 g, 2 mmol) was added. The reaction mixture was refluxed for 15 h, then left to cool. The mixture was poured slowly onto crushed ice and the formed solid was separated by filtration, washed with water several times, and recrystallized from ethanol to yield the 2,4-dichloro compounds **3a,b**.

*2,4-Dichloro-7,9-dimethylpyrido[3′,2′:4,5]thieno[3,2-d]pyrimidine* (**3a**) was obtained from **2a** (0.25 g, 1 mmol) in 67% (0.19 g) yield as a buff solid, m.p. 250–251 °C. IR (KBr, υ_max_/cm^−1^): 3015 (CH-aromatic), 2920, 2853 (CH-aliphatic), 825, 768 (C-Cl); ^1^H-NMR (DMSO-d6, 400 MHz): δ = 2.59 (s, 3H, CH_3_), 2.76 (s, 3H, CH_3_), 7.34 (s, 1H, Ar-H); ^13^C-NMR (DMSO-d6, 100 MHz): δ = 20.43 (CH_3_), 24.29 (CH_3_), 116.33, 120.63, 123.01, 140.22, 145.69, 152.10, 159.19, 161.22, 163.17 (Ar-C); ESI-MS: *m*/*z* = 283.13 [M − H^+^]. Anal. Calcd. for C_11_H_7_Cl_2_N_3_S (284.16): C, 46.50; H, 2.48; N, 14.79; S, 11.28% Found: C, 46.32, H, 2.59; N, 14.63; S, 11.09%.

*2,4-Dichloro-7-phenyl-9-(p-tolyl)pyrido[3′,2′:4,5]thieno[3,2-d]pyrimidine* (**3b**) was obtained from 2b (0.39 g, 1 mmol) in 73% yield (0.31 g) as a brown solid, m.p. 220 °C. IR (KBr, υ_max_/cm^−1^): 3094 (CH-aromatic), 2922 (CH-aliphatic), 1621 (C=N), 819, 768 (C-Cl); ^1^H-NMR (DMSO-d6, 400 MHz): δ = 2.47 (s, 3H, CH_3_), 7.34 (d, 2H, *J* = 8.4 Hz, Ar-H), 7.49–7.61 (m, 3H, Ar-H), 7.77 (d, 2H, *J* = 8.4 Hz, Ar-H), 8.14 (s, 1H, Ar-H), 8.24 (d, 2H, *J* = 3.6 Hz, Ar-H); ^13^C-NMR (DMSO-d6, 100 MHz): δ = 21.38 (CH_3_), 113.52, 118.73, 119.18, 127.79, 129.21, 129.46, 130.38, 132.69, 133.23, 137.29, 138.95, 140.27, 150.70, 155.97, 157.27, 160.01, 161.26 (Ar-C); ESI-MS: *m*/*z* = 421.31 [M − H^+^]. Anal. Calcd. for C_22_H_13_Cl_2_N_3_S (422.33): C, 62.57; H, 3.10; N, 9.95; S, 7.59% Found: C, 62.76, H, 3.35; N, 9.73; S, 7.78 %.

#### 3.1.4. Synthesis of 2-(Chloromethyl)pyrido[3′,2′:4,5]thieno[3,2-*d*]pyrimidin-4(3*H*)-ones **4a,b**

To a cold solution of **1a,b** (5 mmol) in acetonitrile (30 mL) at 0–5 °C, 2-chloroacetyl chloride (0.56 g, 5 mmol) was added dropwise while stirring. After addition, the reaction mixture was stirred for 1 h at room temperature and the solution was evaporated until dryness under reduced pressure, and then the oily residue was treated with hot petroleum ether 40–60. Then the formed solid was collected by filtration and recrystallized from ethanol to yield **4a,b**.

*2-(Chloromethyl)-7,9-dimethylpyrido[3′,2′:4,5]thieno[3,2-d]pyrimidin-4(3H)-one* (**4a**) was obtained from **1a** (1.11 g, 5 mmol) in 78% yield (1.09 g) as a yellow solid, m.p. 330–331 °C. IR (KBr, υ_max_/cm^−1^): 3387 (NH), 3097 (CH-aromatic), 2821 (CH-aliphatic), 1669 (C=O), 772 (C-Cl); ^1^H-NMR (DMSO-d6, 400 MHz): δ = 2.59 (s, 3H, CH_3_), 2.88 (s, 3H, CH_3_), 4.66 (s, 2H, CH_2_Cl), 7.28 (s, 1H, Ar-H), 13.20 (s, 1H, NH, D_2_O exchangeable); ^13^C-NMR (DMSO-d6, 100 MHz): δ = 19.17, 24.37 (2CH_3_), 52.80 (CH_2_-Cl), 123.27, 124.88, 144.56, 147.42, 152.87, 158.65, 159.42, 160.22 (Ar-C), 161.93 (C=O); ESI-MS: *m*/*z* = 278.69 [M − H^+^]. Anal. Calcd. for C_12_H_10_ClN_3_OS (279.74): C, 51.52; H, 3.60; N, 15.02; S, 11.46% Found: C, 51.28; H, 3.35; N, 14.82; S, 11.20%.

*2-(Chloromethyl)-7-phenyl-9-(p-tolyl)pyrido[3′,2′:4,5]thieno[3,2-d]pyrimidin-4(3H)-one* (**4b**) was obtained from **1b** (1.79 g, 5 mmol) in 82% yield (1.71 g) as a yellow solid, m.p. 310 °C. IR (KBr, υ_max_/cm^−1^): 3437 (NH), 3119 (CH-aromatic), 2920 (CH-aliphatic), 1669 (C=O), 776 (C-Cl); ^1^H-NMR (DMSO-d6, 400 MHz): δ = 2.43 (s, 3H, CH_3_), 4.38 (s, 2H, CH_2_Cl), 7.32 (d, 2H, *J* = 6.4 Hz, Ar-H), 7.45–7.57 (m, 3H, Ar-H), 7.66 (d, 2H, *J* = 6.4 Hz, Ar-H), 7.99 (s, 1H, Ar-H), 8.21 (d, 2H, *J* = 8.4 Hz, Ar-H), 9.79 (s, 1H, NH, D_2_O exchangeable); ^13^C-NMR (DMSO-d6, 100 MHz): δ = 21.32 (CH_3_), 53.71 (CH_2_-Cl), 119.37, 126.63, 127.15, 127.94, 129.16, 129.51, 130.08, 132.64, 139.94, 140.57, 147.84, 148.12, 149.61,154.64, 155.69, 159.02, 160.33 (Ar-C, C=O); ESI-MS: *m*/*z* = 416.87 [M − H^+^]. Anal. Calcd. for C_23_H_16_ClN_3_OS (417.91): C, 66.10; H, 3.86; N, 10.06; S, 7.67% Found: C, 66.41, H, 4.06; N, 9.86; S, 7.88%.

#### 3.1.5. Synthesis of 2-Substituted-7-phenyl-9-(p-tolyl)pyrido[3′,2′:4,5]thieno[3,2-*d*]pyrimidin-4(3*H*)-ones **5a,b**

A mixture of **4b** (0.42 g, 1 mmol) and the appropriate amine (1 mmol) in DMF (15 mL) was refluxed for 1 hr, and then the reaction mixture was poured onto cold water. The obtained precipitate was separated by filtration, washed with water, and recrystallized from ethanol to yield **5a,b**.

*2-(Morpholinomethyl)-7-phenyl-9-(p-tolyl)pyrido[3′,2′:4,5]thieno[3,2-d]pyrimidin-4(3H)-one* (**5a**) was obtained by reaction of **4b** with morpholine (0.087 g, 1mmol) in 79% yield (0.37 g) as a pale yellow solid, m.p. 245 °C. IR (KBr, υ_max_/cm^−1^): 3440 (NH), 3094 (CH-aromatic), 2919, 2854 (CH-aliphatic), 1662 (C=O); ^1^H-NMR (DMSO-d6, 400 MHz): δ = 2.38 (s, 4H, 2CH_2_N-morpholine), 2.39 (s, 3H, CH_3_), 3.29 (s, 2H, CH_2_N), 3.54 (s, 4H, 2CH_2_O), 7.28 (d, 2H, *J* = 10.4 Hz, Ar-H), 7.49–7.60 (m, 3H, Ar-H), 7.63 (d, 2H, *J* =10.4 Hz, Ar-H), 7.97 (s, 1H, Ar-H), 8.27 (d, 2H, *J* = 8.4 Hz, Ar-H), 12.66 (s, 1H, NH, D_2_O exchangeable); ^13^C-NMR (DMSO-d6, 100 MHz): δ = 21.44 (CH_3_), 53.37 (2CH_2_N-morpholine), 60.65 (CH_2_N), 66.56 (2CH_2_O), 119.80, 127.49, 127.82, 128.47, 129.49, 130.45, 130.83, 132.14, 139.71, 140.50, 150.02, 151.48, 155.65, 157.06, 158.75 (Ar-C), 163.50 (C=O); ESI-MS: *m*/*z* = 467.50 [M − H^+^]. Anal. Calcd. for C_27_H_24_N_4_O_2_S (468.58): C, 69.21; H, 5.16; N, 11.96; S, 6.84 % Found: C, 69.48; H, 5.39; N, 11.72; S, 6.51%.

*2-((4-Methylpiperazin-1-yl)methyl)-7-phenyl-9-(p-tolyl)pyrido[3′,2′:4,5]thieno[3,2-d]pyrimidin-4(3H)-one* (**5b**) was obtained by reaction of **4b** with 4-methylpiperazine (0.100 g, 1mmol) in 73% yield (0.35 g) as a yellowish white solid, m.p. 260–261 °C. IR (KBr, υ_max_/cm^−1^): 3433 (NH), 3032 (CH-aromatic), 2920 (CH-aliphatic), 1655 (C=O); ^1^H-NMR (CDCl_3_, 400 MHz): δ = 2.48 (s, 3H, CH_3_), 2.54 (s, 3H, NCH_3_), 2.81–3.01 (m, 8H, 4CH_2_N piperazine), 3.57 (s, 2H, NCH_2_), 7.32 (d, 2H, *J* = 8 Hz, Ar-H), 7.48–7.54 (m, 3H, Ar-H), 7.64 (d, 2H, *J* = 8.4 Hz, Ar-H), 7.81 (s, 1H, Ar-H), 8.17 (d, 2H, *J* = 6.8 Hz, Ar-H), 11.32 (s, 1H, NH, D_2_O exchangeable); ^13^C-NMR (DMSO-d6, 100 MHz): δ = 21.34 (CH_3_), 48.22 (CH_3_N), 56.13, 57.56 (4CH_2_N-piperazine), 61.49 (CH_2_N), 119.11, 126.96, 127.21, 127.79, 128.90, 129.01, 129.44, 130.08, 132.56, 139.95, 140.21, 145.01, 149.47, 150.17, 155.56, 157.15, 158.44 (Ar-C), 163.25 (C=O); ESI-MS: *m*/*z* = 480.64 [M − H^+^]. Anal. Calcd. for C_28_H_27_N_5_OS (481.62): C, 69.83; H, 5.65; N, 14.54; S, 6.66% Found: C, 69.62, H, 5.90; N, 14.21; S, 6.79%.

#### 3.1.6. Synthesis of 1′*H*-Spiro[cycloalkane-1,2′-pyrido[3′,2′:4,5]thieno[3,2-*d*]pyrimidin]-4′(3′*H*)-ones **6a–d**

A mixture of **1a,b** (0.02 mol)) and the appropriate cyclic ketone (0.03 mol) in DMF (50 mL) containing zinc chloride anhydrous (2.72 g, 0.02 mol) was heated under reflux for 6 h, and then the reaction mixture was poured onto cold water. The obtained precipitate was separated by filtration, washed with water, and recrystallized from DMF/H_2_O to yield **6a–d.**

*7′,9′-Dimethyl-1′H-spiro[cyclopentane-1,2′-pyrido[3′,2′:4,5]thieno[3,2-d]pyrimidin]-4′(3′H)-one* (**6a**) was obtained by reaction of **1a** (4.42 g, 0.02 mol) with cyclopentanone (2.52 g, 0.03 mol) in 75% yield (4.31 g) as a brown solid, m.p. 245–246 °C. IR (KBr, υmax/cm−1): 3319, 3210 (2NH), 3070 (CH-aromatic), 2921, 2828 (CH-aliphatic), 1650 (C=O); 1H-NMR (DMSO-d6, 400 MHz): δ = 1.72–2.07 (m, 8H, 4CH2), 2.51 (s, 3H, CH3), 2.66)s, 3H, CH3), 6.48 (s, 1H, NH, D2O exchangeable), 7.04 (s,1H, Ar-H), 9.82 (s, 1H, NH, D2O exchangeable); 13C-NMR (DMSO-d6, 100 MHz): δ = 20.41 (CH3), 22.65, (2CH2), 24.33 (CH3), 37.55 (2CH2), 70.15 (spiro C), 122.54, 123.29, 135.88, 145.63, 148.71, 159.30, 161.57 (Ar-C), 167.65 (C=O); ESI-MS: *m/z* = 286.41 [M − H^+^]. Anal. Calcd. for C15H17N3OS (287.38): C, 62.69; H, 5.96; N, 14.62; S, 11.16% Found: C, 62.52, H, 5.74; N, 14.41; S, 10.98%.

*7′-Phenyl-9′-(p-tolyl)-1′H-spiro[cyclopentane-1,2′-pyrido[3′,2′:4,5]thieno[3,2-d]pyrimidin]-4′(3′H)-one* (**6b**) was obtained by reaction of **1b** (7.19 g, 0.02 mol) with cyclopentanone (2.52 g, 0.03 mol) in 71% yield (6.04 g) as a yellow solid, m.p. 210 °C. IR (KBr, υ_max_/cm^−1^): 3410, 3200 (2NH), 3055 (CH-aromatic), 2919 (CH-aliphatic), 1644 (C=O); ^1^H-NMR (DMSO-d6, 400 MHz): δ = 1.21–1.85 (m, 8H, 4CH_2_), 2.43 (s, 3H, CH_3_), 5.94 (s, 1H, NH, D_2_O exchangeable), 7.29 (d, 2H, *J* = 7.4 Hz, Ar-H), 7.41–7.62 (m, 5H, Ar-H), 7.81(s, 1H, NH, D_2_O exchangeable), 7.96 (s, 1H, Ar-H), 8.22 (d, 2H, *J* = 10.2 Hz, Ar-H); ^13^C-NMR (DMSO-d6, 100 MHz): δ = 21.36, 21.54 (2CH_2_, CH_3_), 38.33 (2CH_2_), 70.81 (spiro C), 118.26, 121.25, 127.54, 127.65, 129.39, 129.79, 130.32, 133.82, 139.15, 139.31, 148.17, 150.62, 155.15, 163.25 (Ar-C), 167.38 (C=O); ESI-MS: *m*/*z* = 424.50 [M − H^+^]. Anal. Calcd. for C_26_H_23_N_3_OS (425.55): C, 73.38; H, 5.45; N, 9.87; S, 7.53% Found: C, 73.64, H, 5.21; N, 10.08; S, 7.38%.

*7′,9′-Dimethyl-1′H-spiro[cyclohexane-1,2′-pyrido[3′,2′:4,5]thieno[3,2-d]pyrimidin]-4′(3′H)-one* (**6c**) was obtained by reaction of **1a** (4.42 g, 0.02 mol) with cyclohexanone (2.94 g, 0.03 mol) in 79% yield (4.76 g) as a brown solid, m.p. 290–291 °C. IR (KBr, υ_max_/cm^−1^): 3317, 3168 (2NH), 3043 (CH-aromatic), 2932 (CH-aliphatic), 1649 (C=O); ^1^H-NMR (DMSO-d6, 400 MHz): δ = 1.23–2.06 (m, 10H, 5CH_2_), 2.51 (s, 3H, CH_3_), 2.71 (s, 3H, CH_3_), 5.91 (s, 1H, NH, D_2_O exchangeable), 7. 07 (s, 1H, Ar-H), 7.86 (s, 1H, NH, D_2_O exchangeable); ^13^C-NMR (DMSO-d6, 100 MHz): δ = 19.36, 20.16, 22.27, 24.32 (3CH_2_, 2CH_3_), 36.24 (2CH_2_), 69.49 (spiro C), 122.11, 123.58, 134.96, 145.31, 148.44, 159.14, 161.51 (Ar-C), 167.80 (C=O); ESI-MS: *m/z* = 300.46 [M − H^+^]. Anal. Calcd. for C_16_H_19_N_3_OS (301.41): C, 63.76; H, 6.35; N, 13.94; S, 10.64% Found: C, 63.49, H, 6.17; N, 13.69; S, 10.41%.

*7′-Phenyl-9′-(p-tolyl)-1′H-spiro[cyclohexane-1,2′-pyrido[3′,2′:4,5]thieno[3,2-d]pyrimidin]-4′(3′H)-one* (**6d**) was obtained by reaction of **1b** (7.19 g, 0.02 mol) with cyclohexanone (2.94 g, 0.03 mol) in 77% yield (6.77 g) as a yellow solid, m.p. 263 °C. IR (KBr, υ_max_/cm^−1^): 3415, 3166 (2NH), 3053 (CH-aromatic), 2924, 2854 (CH-aliphatic), 1660 (C=O); ^1^H-NMR (DMSO-d6, 400 MHz): δ = 1.34–1.87 (m, 10H, 5CH_2_), 2.42 (s, 3H, CH_3_), 4.59 (s, 1H, NH, D_2_O exchangeable), 7.38 (d, 2H, *J* = 6.8 Hz, Ar-H), 7.49–7.54 (m, 5H, Ar-H), 7.83 (s, 1H, Ar-H), 8.06 (s, 1H, NH, D_2_O exchangeable), 8.21 (d, 2H, *J* = 6 Hz, Ar-H); ^13^C-NMR (DMSO-d6, 100 MHz): δ = 19.96, 21.38, 22.54 (3CH_2_, CH_3_), 35.86 (2CH_2_), 70.67 (spiro C), 118.52, 121.27, 127.48, 127.84, 129.24, 129.67, 129.96, 130.86, 133.48, 137.29, 139.20, 139.50, 148.67, 155.50, 156.14 (Ar-C), 167.73 (C=O); ESI-MS: *m/z* = 438.52 [M − H^+^]. Anal. Calcd. for C_27_H_25_N_3_OS (439.58): C, 73.77; H, 5.73; N, 9.56; S, 7.29% Found: C, 73.54, H, 5.49; N, 9.69; S, 7.59%.

#### 3.1.7. Synthesis of 1′*H*-Spiro[cycloalkane-1,2′-pyrido[3′,2′:4,5]thieno[3,2-*d*]pyrimidine]-4′(3′*H*)-thiones **7a–d**

A mixture of **6a–d** (5 mmol) and phosphorus pentasulfide (1.11 g, 5 mmol) in pyridine (40 mL) was heated under reflux for 12 h. The reaction mixture was poured onto cold water and left overnight. The formed solid was separated by filtration, washed with water, and recrystallized from 1,4-dioxane to yield compounds **7a–d.**

*7′,9′-Dimethyl-1′H-spiro[cyclopentane-1,2′-pyrido[3′,2′:4,5]thieno[3,2-d]pyrimidine]-4′(3′H)-thione* (**7a**) was obtained from **6a** (1.44 g, 5 mmol) in 63% yield (0.95 g) as a burnt orange solid, m.p. 220–221 °C. IR (KBr, υ_max_/cm^−1^): 3372, 3157 (2NH), 3063 (CH-aromatic), 2922 (CH-aliphatic), 1247 (C=S); ^1^H-NMR (DMSO-d6, 400 MHz): δ = 1.36–2.03 (m, 8H, 4CH_2_), 2.57 (s, 3H, CH_3_), 2.73 (s, 3H, CH3), 6.49 (s, 1H, NH,D_2_O exchangeable), 7.03 (s,1H, Ar-H), 9.85 (s, 1H, NH,D_2_O exchangeable); ^13^C-NMR (DMSO-d6, 100 MHz): δ =19.56, 22.53 (CH_3_, 2CH_2_), 24.43 (CH_3_), 37.67 (2CH_2_), 78.59 (spiro C), 117.93, 122.11, 123.15, 139.87, 146.17, 159.21, 162.86 (Ar-C),182.25 (C=S); ESI-MS: *m/z* = 302.40 [M − H^+^]. Anal. Calcd. for C_15_H_17_N_3_S_2_ (303.44): C, 59.37; H, 5.65; N, 13.85; S, 21.13% Found: C, 59.09, H, 5.47; N, 13.56; S, 20.89%.

*7′-Phenyl-9′-(p-tolyl)-1′H-spiro[cyclopentane-1,2′-pyrido[3′,2′:4,5]thieno[3,2-d]pyrimidine]-4′(3′H)-thione* (**7b**) was obtained from **6b** (2.13 g, 5 mmol) in 68% yield (1.50 g) as a brown solid, m.p. 180 °C. IR (KBr, υ_max_/cm^−1^): 3402, 3186 (2NH), 3055 (CH-aromatic), 2923, 2852 (CH-aliphatic), 1232 (C=S); ^1^H-NMR (DMSO-d6, 400 MHz): δ = 1.34–2.01 (m, 8H, 4CH_2_), 2.42 (s, 3H, CH_3_), 4.67 (s, 1H, NH, D_2_O exchangeable), 7.42 (d, 2H, *J* = 7.2 Hz, Ar-H), 7.48–7.53 (m, 5H, Ar-H), 7.83 (s, 1H, Ar-H), 8.22 (d, 2H, *J* = 7.2 Hz, Ar-H), 9.68 (s, 1H, NH, D_2_O exchangeable); ^13^C-NMR (DMSO-d6, 100 MHz): δ = 21.47, 21.96 (2CH_2_, CH_3_), 36.50 (2CH_2_), 77.79 (spiro C), 118.42, 121.20, 124.67, 127.47, 127.71, 129.11, 129.74, 130.20, 133.56, 139.21, 139.34, 148.17, 149.62, 155.15, 163.25 (Ar-C), 183.35 (C=S); ESI-MS: *m/z* = 440.69 [M − H^+^]. Anal. Calcd. for C_26_H_23_N_3_S_2_ (441.61): C, 70.72; H, 5.25; N, 9.52; S, 14.52% Found: C, 70.53, H, 5.01; N, 9.37; S, 14.38%.

*7′,9′-Dimethyl-1′H-spiro[cyclohexane-1,2′-pyrido[3′,2′:4,5]thieno[3,2-d]pyrimidine]-4′(3′H)-thione* (**7c**) was obtained from **6c** (1.51 g, 5 mmol) in 71% yield (1.13 g) as a greenish grey solid, m.p. 201 °C. IR (KBr, υ_max_/cm^−1^): 3427, 3190 (2NH), 3048 (CH-aromatic), 2929, 2853 (CH-aliphatic), 1234 (C=S); ^1^H-NMR (DMSO-d6, 400 MHz): δ = 1.21–2.11 (m, 10H, 5CH_2_), 2.51 (s, 3H, CH_3_), 2.71 (s, 3H, CH_3_), 6.22 (s, 1H, NH, D_2_O exchangeable), 7. 07 (s, 1H, Ar-H), 9.68 (s, 1H, NH, D_2_O exchangeable); ^13^C-NMR (DMSO-d6, 100 MHz): δ = 19.26, 22.02, 24.32, 25.15 (3CH_2_, 2CH_3_), 34.46 (2CH_2_), 70.16 (spiro C), 118.38,122.29, 123.61, 138.66, 146.42, 159.31, 162.71 (Ar-C), 181.33 (C=S); ESI-MS: *m/z* = 316.40 [M − H^+^]. Anal. Calcd. for C_16_H_19_N_3_S_2_ (317.47): C, 60.53; H, 6.03; N, 13.24; S, 20.20% Found: C, 60.79, H, 6.17; N, 13.49; S, 20.44%.

*7′-Phenyl-9′-(p-tolyl)-1′H-spiro[cyclohexane-1,2′-pyrido[3′,2′:4,5]thieno[3,2-d]pyrimidine]-4′(3′H)-thione* (**7d**) was obtained from **6d** (2.19 g, 5 mmol) in 66% yield (1.50 g) as a yellow solid, m.p. 178–179 °C. IR (KBr, υ_max_/cm^−1^): 3402, 3188 (NH), 3068 (CH-aromatic), 2923, 2852 (CH-aliphatic), 1232 (C=S); ^1^H-NMR (DMSO-d6, 400 MHz): δ = 1.2 4–2.07 (m, 10H, 5CH_2_), 2.43 (s, 3H, CH_3_), 4.79 (s, 1H, NH, D_2_O exchangeable), 7.39 (d, 2H, *J* = 8.4 Hz, Ar-H), 7.48–7.57 (m, 5H, Ar-H), 7.87 (s, 1H, Ar-H), 9.66 (s, 1H, NH, D_2_O exchangeable), 8.22 (d, 2H, *J* = 6.4 Hz, Ar-H); ^13^C-NMR (DMSO-d6, 100 MHz): δ = 21.22, 21.47, 24.46 (3CH_2_, CH_3_), 34.87 (2CH_2_), 70.46 (spiro C), 118.21, 121.45, 124.25, 127.39, 127.73, 129.39, 129.66, 130.42, 133.92, 137.90, 139.96, 140.00, 148.82, 156.27, 163.83 (Ar-C), 182.12 (C=S); ESI-MS: *m/z* = 454.60 [M − H^+^]. Anal. Calcd. for C_27_H_25_N_3_S_2_ (455.64): C, 71.17; H, 5.53; N, 9.22; S, 14.07% Found: C, 71.36, H, 5.19; N, 9.46; S, 13.89%.

#### 3.1.8. Synthesis of 2-((1′*H*-Spiro[cycloalkane-1,2′-pyrido[3′,2′:4,5]thieno[3,2-*d*]pyrimidin]-4′-yl)sulfanyl)acetamides **8a,b**

A mixture of compounds **7c,b** (2 mmol) and 2-chloroacetamide (0.187 g, 2 mmol) in DMF (30 mL) containing sodium carbonate anhydrous (0.5 g) was refluxed for 6 h. The reaction mixture was poured onto iced water and the medium was neutralized with 1N HCl to pH = 7. The formed precipitate was separated by filtration, washed with water, and recrystallized from chloroform to yield the acetamide derivatives **8a,b**.

*2-((7′,9′-Dimethyl-1′H-spiro[cyclohexane-1,2′-pyrido[3′,2′:4,5]thieno[3,2-d]pyrimidin]-4′-yl)sulfanyl)acetamide* (**8a**) was obtained from **7c** (0.63 g, 2 mmol) in 73% yield (0.55 g) as a brown solid, m.p. 160–161 °C. IR (KBr, υ_max_/cm^−1^): 3403, 3233 (NH), 3048 (CH-aromatic), 2926, 2854 (CH-aliphatic), 1674 (C=O); ^1^H-NMR (DMSO-d6, 400 MHz): δ = 1.22–2.09 (m, 10H, 5CH_2_), 2.68 (s, 3H, CH_3_), 2.76 (s, 3H, CH_3_), 4.05 (s, 2H, SCH_2_), 6.24 (s, 1H, NH, D_2_O exchangeable), 7. 09 (s, 1H, Ar-H), 7.35 (s, 2H, NH_2_, D_2_O exchangeable); ^13^C-NMR (DMSO-d6, 100 MHz): δ = 19.21, 22.71, 24.73, 25.41 (3CH_2_, 2CH_3_), 36.82 (2CH_2_), 41.93 (SCH_2_), 72.43 (spiro C), 118.78, 122.54, 123.01, 124.67, 144.63, 147.53, 159.35, 163.15 (Ar-C), 168.23 (C=O); ESI-MS: *m/z* = 373.50 [M − H^+^]. Anal. Calcd. for C_18_H_22_N_4_OS_2_ (374.52): C, 57.73; H, 5.92; N, 14.96; S, 17.12% Found: C, 57.49, H, 5.18; N, 14.58; S, 17.43%.

*2-((7′-Phenyl-9′-(p-tolyl)-1′H-spiro[cyclopentane-1,2′-pyrido[3′,2′:4,5]thieno[3,2-d]pyrimidin]-4′-yl)sulfanyl)acetamide* (**8b**) was obtained from **7b** (0.88 g, 2 mmol) in 69% yield (0.69 g) as a brown solid, m.p. 148–149 °C. IR (KBr, υ_max_/cm^−1^): 3400, 3200 (NH), 3054 (CH-aromatic), 2918, 2853 (CH-aliphatic), 1667 (C=O); ^1^H-NMR (DMSO-d6, 400 MHz): δ = 1.21–2.03 (m, 8H, 4CH_2_), 2.43 (s, 3H, CH_3_), 4.19 (s, 2H, SCH_2_), 6.86 (s, 1H, NH, D_2_O exchangeable), 7.34 (d, 2H, *J* = 5.6 Hz, Ar-H), 7.44–7.63 (m, 5H, Ar-H), 7.72 (s, 2H, NH_2_, D_2_O exchangeable), 7.83 (s, 1H, Ar-H), 8.24 (d, 2H, *J* = 6.8 Hz, Ar-H); ^13^C-NMR (DMSO-d6, 100 MHz): δ = 21.43 (CH_3_), 29.61(2CH_2_), 33.86 (2CH_2_), 41.41(SCH_2_), 74.27 (spiro C), 118.55, 122.05, 126.94, 127.85, 127.96, 128.90, 129.50, 130.44, 133.49, 139.05, 140.57, 147.93, 150.02,155.45, 157.40, 163.26 (Ar-C), 169.38 (C=O); ESI-MS: *m/z* = 497.68 [M − H^+^]. Anal. Calcd. for C_28_H_26_N_4_OS_2_ (498.66): C, 67.44; H, 5.26; N, 11.24; S, 12.86% Found: C, 67.63, H, 5.44; N, 11.50; S, 13.05%.

#### 3.1.9. Synthesis of 4′-((oxiran-2-ylmethyl)sulfanyl)-1′*H*-spiro[cycloalkane-1,2′-pyrido[3′,2′:4,5]thieno-[3,2-*d*]pyrimidine] **9a,b**

A mixture of compounds **7c,b** (1mmol)) and epichlorohydrin (0.092 g, 1mmol) in acetone (20 mL) containing triethyl amine (0.1 mL) was refluxed for 5 h. The solvent was evaporated until dryness and the oily residue was treated with hot petroleum ether (30 mL). The formed solid was separated by filtration and recrystallized from ethanol to yield **9a,b**.

*7′,9′-Dimethyl-4′-((oxiran-2-ylmethyl)sulfanyl)-1′H-spiro[cyclohexane-1,2′-pyrido[3′,2′:4,5] thieno[3,2 -d]pyrimidine]* (**9a**), was obtained from **7c** (0.32 g, 1 mmol) in 78% yield (0.29 g) as a grey solid, m.p. 155 °C. IR (KBr, υ_max_/cm^−1^): 3430 (NH), 3040 (CH-aromatic), 2928, 2856 (CH-aliphatic), 1623 (C=N); ^1^H-NMR (DMSO-d6, 400 MHz): δ = 1.24–1.89 (m, 10H, 5CH_2_), 2.60 (s, 3H, CH_3_), 2.74 (s, 3H, CH_3_), 2.86–2.94 (2m, 2H, OCH_2_- oxirane), 3.67 (d, 2H, *J* = 10.8 Hz, SCH_2_), 3.72–3.77 (m, 1H, OCH-oxirane), 6.12 (s, 1H, NH, D_2_O exchangeable), 7. 06 (s, 1H, Ar-H); ^13^C-NMR (DMSO-d6, 100 MHz): δ = 19.27, 22.91, 24.70, 25.33 (3CH_2_, 2CH_3_), 32.19 (SCH_2_), 37.02 (2CH_2_), 44.76, 52.45 (OCH_2_, OCH, oxirane), 70.03 (spiro C), 118.74, 122.33, 123.12, 124.67, 145.00, 147.21, 159.19, 163.66 (Ar-C); ESI-MS: *m/z* = 372.60 [M − H^+^]. Anal. Calcd. for C_19_H_23_N_3_OS_2_ (373.53): C, 61.09; H, 6.21; N, 11.25; S, 17.17% Found: C, 61.23, H, 6.44; N, 11.51; S, 17.01%.

*4′-((Oxiran-2-ylmethyl)sulfanyl)-7′-phenyl-9′-(p-tolyl)-1′H-spiro[cyclopentane-1,2′-pyrido[3′,2′:4,5]thieno[3,2-d]pyrimidine]* (**9b**) was obtained from **7b** (0.44 g, 1 mmol) in 74% yield (0.37 g) as a pale yellow solid, m.p. 120 °C. IR (KBr, υ_max_/cm^−1^): 3422 (NH), 3094 (CH-aromatic), 2919 (CH-aliphatic), (C=N); ^1^H-NMR (DMSO-d6, 400 MHz): δ = 1.35–1.94 (m, 8H, 4CH_2_), 2.43 (s, 3H, CH_3_), 2.94–3.13 (2m, 2H, OCH_2_- oxirane), 3.51 (d, 2H, *J* = 6.0 Hz, SCH_2_), 3.91–3.95 (m, 1H, OCH-oxirane), 6.87 (s, 1H, NH, D_2_O exchangeable), 7.32 (d, 2H, *J* = 7.2 Hz, Ar-H), 7.48–7.69 (m, 5H, Ar-H), 7.87 (s, 1H, Ar-H), 8.26 (d, 2H, *J* = 7.6 Hz, Ar-H); ^13^C-NMR (DMSO-d6, 100 MHz): δ = 21.40 (CH_3_), 29.15 (2CH_2_), 31.88 (SCH_2_), 36.33 (2CH_2_), 46.33, 53.45 (OCH_2_, OCH, oxirane), 69.33 (spiro C), 118.49, 123.62, 127.81, 127.93, 128.78, 128.94, 129.43, 129.50, 130.53, 133.77, 139.17, 140.27, 148.73, 152.73,156.17, 163.83 (Ar-C); ESI-MS: *m/z* = 496.70 [M − H^+^]. Anal. Calcd. for C_29_H_27_N_3_OS_2_ (497.68): C, 69.99; H, 5.47; N, 8.44; S, 12.88% Found: C, 69.81, H, 5.31; N, 8.20; S, 12.63 %.

### 3.2. Antimicrobial Assay

All synthesized compounds (**2a,b**–**9a,b**) were screened for their in vitro antimicrobial activity against five bacterial strains (*S. aureus* 25923, *B. subtilis* 6633, *B. cereus* 33018, *E. coli* 8739, *S. typhimurium* 14028), three yeasts (*C. albicans* 10231, *C. tropicals* 750, *S. cerevisiae*) and two fungi (*A. flavus*, *A. niger* EM77). The MIC values (in μg/mL) of the tested compounds were determined using the broth dilution method and are listed in Table 1 and Table 2 [57]. (More details are provided in the Supplementary Materials).

### 3.3. In Vitro Cytotoxicity Screening

The in vitro cytotoxic activity of the target compounds **2a,b**–**9a,b** was screened against HepG-2 and MCF-7 cancer cell lines, as well as the WISH normal cell line for the most active compounds, using the MTT assay [53]. The cells used in the cytotoxicity assays were cultured in a RPMI 1640 medium supplemented with 10% fetal calf serum. The cytotoxicity was estimated as IC_50_ in μM for the tested compounds and the reference drug doxorubicin, and are listed in Table 3. (More details are provided in Supplementary Materials).

### 3.4. EGFR Kinase Inhibitory Assay

EGFR kinase inhibitory assays were performed for target compounds **3a**, **4a, 5a**, **6b**, **8b** and **9b** with erlotinib as a reference inhibitor using the EGFR kinase assay kit (Cat. # 40321). The assay kit is designed to measure EGFR Kinase activity for screening applications using Kinase-Glo^®^ MAX as a detection reagent. The luminescence was measured using a microplate reader (Infinite M200 microplate reader, Tecan, Männedorf, Switzerland) [54]. All assays were performed in triplicate and the relative inhibition (%) of inhibitors was then calculated via comparison with the control with no inhibitor. Then, the IC_50_ values (the concentration that provides 50% enzyme inhibition) and their standard deviation (SD) for the tested compounds and the reference drug were determined in μM and are listed in Table 4. (More details are provided in the Supplementary Materials).

### 3.5. Molecular Modeling Studies

To investigate molecular interactions between the most potent compounds and the active site of the epidermal growth factor receptor (EGFR), molecular docking study was performed using molecular operating environment software (MOE 2019.0102). Energy minimization was carried out until a RMSD gradient of 0.1 kcal∙mol^−1^Å^−1^ was achieved using a MMFF94x force field. The co-crystalized ligand (Erlotinib) was used to define the binding site for docking [58,59]. (More details are provided in the Supplementary Materials).

## 4. Conclusions

In conclusion, two novel series of pyridothienopyrimidine and spiro[cyclopentane/hexane-1,2′-pyrido[3′,2′:4,5]thieno[3,2-*d*]pyrimidine] derivatives **2a,b**–**5a,b** and **6a,b**–**9a,b**, were synthesized and structurally elucidated. The new derivatives were subjected to in vitro antimicrobial screening against a panel of bacterial and fungal pathogens. According to the MIC values, derivatives **3a**, **4a,b**, **5a**, **6b,c**, **7c**, **8b**, and **9b** exhibited significant antibacterial and antifungal activity with MIC ranges of 4–16 μg/mL, compared to amoxicillin trihydrate and clotrimazole as reference drugs with MIC ranges of 4–16 µM.

Furthermore, all new derivatives were evaluated as cytotoxic agents against HepG2 and MCF7 cancer cell lines. Compounds **9b**, **5a**, **3a**, **6b**, **8b**, **4a** produced the most potent antiproliferative activity with IC_50_ values ranging between 1.17–2.99 µM against HepG2 cells and IC_50_s ranging between 1.52–15.42 µM against MCF-7 cells, compared to doxorubicin as reference drug with IC_50_s of 2.85 and 3.58 µM, respectively. In addition, these derivatives exhibited a promising safety profile when evaluated against the human normal WISH cell line.

Compounds **3a**, **4a, 5a**, **6b**, **8b**, **9b** presented promising dual antimicrobial and antiproliferative activities, achieving the desired goal of this study.

The suppressing effect of compounds **3a**, **4a, 5a**, **6b**, **8b**, **9b** against EGFR TK was also evaluated. It was detected that compounds **9b**, **5a**, **4a** showed higher suppressing activity than erlotinib with IC_50_ values of 7.27, 9.66 and 27.01 nM, respectively. In addition, molecular docking study was performed to determine the modes of interaction of the examined derivatives with amino acid residues at the active site of EGFR-PK. The docking results revealed that compounds **9a** and **5a** showed higher binding scores (–12.01 and –11.48 kcal/mol) than that of erlotinib (–10.48 kcal/mol).

## Figures and Tables

**Table 1:** In vitro antibacterial activities of the synthesized compounds expressed as MICs (μg/mL) against the tested pathogenic bacteria.

Compd. No.	Gram +ve Bacteria			Gram −ve Bacteria	
	*S. aureus*	*B. subtilis*	*B. cereus*	*E. coli*	*S. typhimurium*
2a	NA	NA	64	NA	NA
2b	64	128	64	128	64
3a	4	4	8	8	16
3b	32	8	32	16	32
4a	4	8	4	8	16
4b	8	128	16	128	128
5a	8	8	16	8	16
5b	8	8	16	32	32
6a	32	16	8	32	16
6b	4	8	16	8	16
6c	4	4	8	8	16
6d	8	32	8	32	32
7a	4	NA	8	8	16
7b	64	64	64	64	128
7c	32	32	8	32	32
7d	4	64	64	32	32
8a	8	16	8	32	32
8b	4	8	16	8	16
9a	16	32	8	32	16
9b	4	4	8	4	16
Amoxicillin	4	8	16	8	16

NA = No Activity (MIC ˃ 128 µg/mL).

**Table 2:** In vitro antifungal activities of the synthesized compounds expressed as MICs (μg/mL) against the tested pathogenic yeasts and fungi.

Compd. No.	Yeasts			Fungi	
	*C. albicans*	*C. tropicals*	*S. cerevisiae*	*A. flavus*	*A. niger*
2a	128	128	16	NA	NA
2b	64	32	32	32	32
3a	16	8	8	8	8
3b	32	16	64	32	16
4a	16	16	8	8	8
4b	8	16	8	16	16
5a	16	8	8	8	16
5b	64	16	16	32	64
6a	64	16	32	64	32
6b	8	8	8	16	8
6c	16	4	16	4	8
6d	64	64	32	32	16
7a	16	16	8	16	16
7b	64	64	32	64	128
7c	16	8	8	4	16
7d	32	64	32	64	64
8a	64	16	16	16	16
8b	8	16	16	8	8
9a	16	32	32	16	64
9b	4	4	8	8	8
Clotrimazole	16	8	8	8	8

NA = No Activity (MIC ˃ 128 µg/mL).

**Table 3:** Cytotoxic activities (IC_50_ µM) of the new compounds and doxorubicin against HepG2, MCF7 and WISH cells.

Compd. No.	HepG2 (µM)	MCF7 (µM)	WISH
2a	56.57 ± 3.64	64.34 ± 2.91	
2b	33.21 ± 1.79	42.39 ± 2.24	
3a	2.31 ± 0.35	7.24 ± 0.64	416.83 ± 15.17
3b	11.34 ± 0.65	24.72 ± 1.32	
4a	2.99 ± 0.15	15.42 ± 0.45	460.23 ± 11.08
4b	36.52 ± 1.82	43.27 ± 2.28	
5a	1.99 ± 0.09	2.79 ± 0.18	408.48 ± 15.93
5b	10.16 ± 0.29	21.06 ± 1.16	
6a	52.18 ± 1.45	77.41 ± 3.62	
6b	2.75 ± 0.13	9.89 ± 0.55	394.98 ± 10.20
6c	12.11 ± 0.33	22.24 ± 0.67	
6d	4.45 ± 0.22	21.67 ± 0.70	
7a	26.05 ± 1.89	28.11 ± 0.92	
7b	39.74 ± 1.89	41.62 ± 2.20	
7c	10.35 ± 0.31	20.9 ± 0.93	
7d	23.25 ± 0.35	26.55 ± 1.62	
8a	6.78 ± 0.73	20.88 ± 1.46	
8b	2.79 ± 0.08	13.54 ± 0.76	401.37± 17.32
9a	4.88 ± 0.65	23.56 ± 1.24	
9b	1.17 ± 0.09	1.52 *±* 0.08	417.55 ± 14.1
Doxorubicin	2.85 ± 0.21	3.58 ± 0.33	432.10 ± 19.30

**Table 4:** In vitro enzymatic inhibitory activity against EGFR kinase.

Compound No.	EGFR IC_50_ (nM)
3a	17.29 ± 0.24
4a	53.57 ± 0.41
5a	9.66 ± 0.08
6b	53.19 ± 0.46
8b	38.44 ± 0.25
9b	7.27 ± 0.11
Erlotinib	27.01 ± 0.16

**Table 5:** Molecular docking results of the most active pyridothienopyrimidine compounds.

Compound	S (kcal/mol)	Amino Acids	Interacting Groups	Type of Bond	Length (Å)
**3a**	–11.42	Val702	N (Pyrimidine)	H-bond acceptor	4.12
		Lys721	N (Pyridine)	H-bond acceptor	3.25
		Met769	Cl (Pyrimidine)	Halogen bond	3.47
		Leu768	Cl (Pyrimidine)	Halogen bond	4.16
		Thr830	Cl (Pyridine)	Halogen bond	3.26
		Met742	Cl (Pyridine)	Halogen bond	4.13
		Thr766	Cl (Pyridine)	Halogen bond	3.03
		Thr830	S	σ-hole bond	3.79
**4a**	–8.94	Lys721	Cl	Halogen bond	3.69
		Cys751	O (C=O)	σ-hole bond	3.78
		Thr766	S	σ-hole bond	4.21
		Met769	S and N (Pyridine)	H-bond acceptor	3.41/3.55
		Leu768	N (Pyridine)	H-bond acceptor	3.84
**5a**	–11.48	Asp831	NH+	Ionic interaction	3.71
		Lys721	O (Morpholine)	H-bond acceptor	3.51
		Cys773	O (C=O)	σ-hole bond	3.46
		Asp776	S	σ-hole bond	4.18
**6b**	–10.06	Leu820	NH	H-bond acceptor	3.78
		Cys751	O (C=O)	H-bond acceptor	3.49
		Gln767	S	σ-hole bond	3.12
		Thr766	S	σ-hole bond	3.87
		Met769	S	H-bond acceptor	3.83
**8b**	–9.11	Leu694	S (Thiophene)	H-bond acceptor	4.38
		Leu694	S (Side chain)	σ-hole bond	3.79
		Thr766	N (Pyridine)	H-bond acceptor	3.71
**9b**	–12.01	Met769	S (thiophene)	H-bond acceptor	4.09
		Leu768	S (thiophene)	H-bond acceptor	4.37
		Leu820	S (thiophene)	H-bond acceptor	4.47
		Thr766	S (Side chain)	σ-hole bond	4.20
		Leu820	N and NH (Pyrimidine)	H-bond acceptor	3.73/3.74
		Val702	O (Oxirane)	H-bond acceptor	3.64
**erlotinib**	–10.48	Leu768	N (Pyrimidine)	H-bond acceptor	3.64
		Met769	N (Pyrimidine)	H-bond acceptor	2.70

## Data Availability

The data presented in this study are available in supplementary material.

## Supplementary Materials

Figures S2–S34: NMR spectral data of the new compounds, S35: in vitro antimicrobial assay, S35: cytotoxicity MTT assay, S36: in vitro EGFR kinase inhibitory assay, S37: docking modeling evaluation.

## Sample Availability

Samples of the compounds 5a, 6c and 7c are available from the authors.
