# Supplementary material for: Novel Pyridothienopyrimidine Derivatives: Design, Synthesis and Biological Evaluation as Antimicrobial and Anticancer Agents
Source: Molecules. 2022 Jan 26;27(3):803. doi: 10.3390/molecules27030803 (PMC8839448; doi:10.3390/molecules27030803)
Supplement: Supplementary file 1 [file molecules-27-00803-s001.zip › molecules-1555857-supplementary.pdf]

Supplementary Materials

Novel Pyridothienopyrimidine Derivatives: Design, Synthesis and Biological Evaluation as Antimicrobial and Anticancer Agents

Eman M. Mohi El-Deen [1,*] , Manal M. Anwar [1], Amina A. Abd El-Gwaad [1], Eman A. Karam [2], Mohamed K. El-Ashrey [3] and Rafika R. Kassab [4]

[1] Department of Therapeutic Chemistry, National Research Centre, Dokki, Cairo 12622, Egypt

[2] Department of Microbial Chemistry, National Research Centre, Dokki, Cairo 12622, Egypt

[3] Department of Pharmaceutical Chemistry, Faculty of Pharmacy, Cairo University, Cairo 11562, Egypt

[4] Department of Chemistry, Faculty of Science, Al-Azhar University, Cairo 11754, Egypt

* Correspondence: e.mohi.2010@live.com; Tel.: +20-0106-385-3338

Table of contents

Subject	Page
^{1}H-NMR and ^{13}C-NMR of the new compounds.	S2–S34
Minimum Inhibitory Concentration (MIC) Measurement.	S35
In vitro anticancer screening (MTT assay)	S35
In vitro EGFR kinase assay	S36
Molecular docking determination	S37

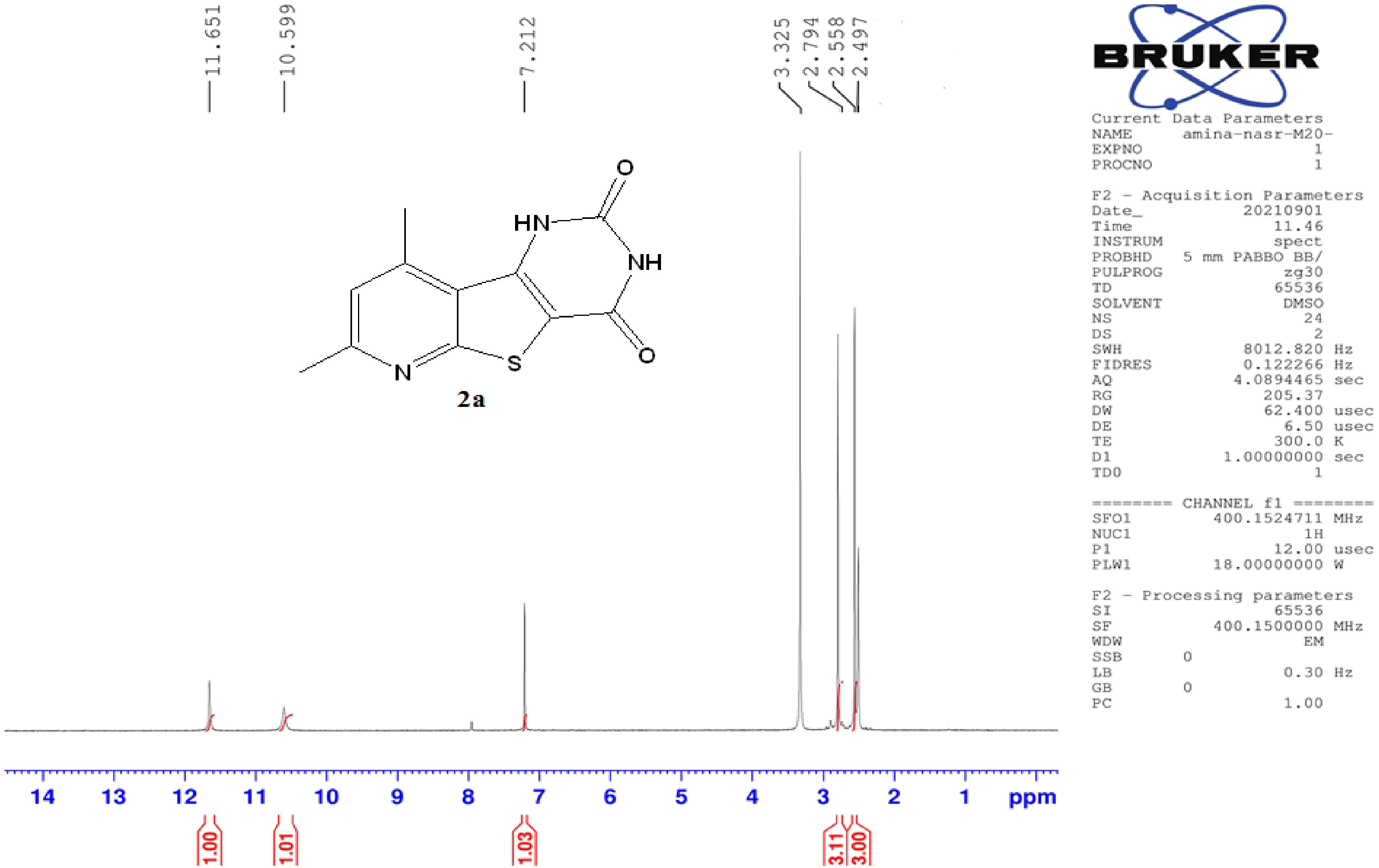
11.651
10.599
7.212
3.325
2.794
2.558
2.497
HN
O
NH
O
N
S
2a
14 13 12 11 10 9 8 7 6 5 4 3 2 1 ppm
1.00
1.01
1.03
3.11
3.00
BRUKER
Current Data Parameters
NAME amina-nasr-M20-
EXPNO 1
PROCNO 1
F2 - Acquisition Parameters
Date_ 20210901
Time 11.46
INSTRUM spect
PROBHD 5 mm PABBO BB/
PULPROG zg30
TD 65536
SOLVENT DMSO
NS 24
DS 2
SWH 8012.820 Hz
FIDRES 0.122266 Hz
AQ 4.0894465 sec
RG 205.37
DW 62.400 usec
DE 6.50 usec
TE 300.0 K
D1 1.00000000 sec
TD0 1
======== CHANNEL f1 ========
SFO1 400.1524711 MHz
NUC1 1H
P1 12.00 usec
PLW1 18.00000000 W
F2 - Processing parameters
SI 65536
SF 400.1500000 MHz
WDW EM
SSB 0
LB 0.30 Hz
GB 0
PC 1.00

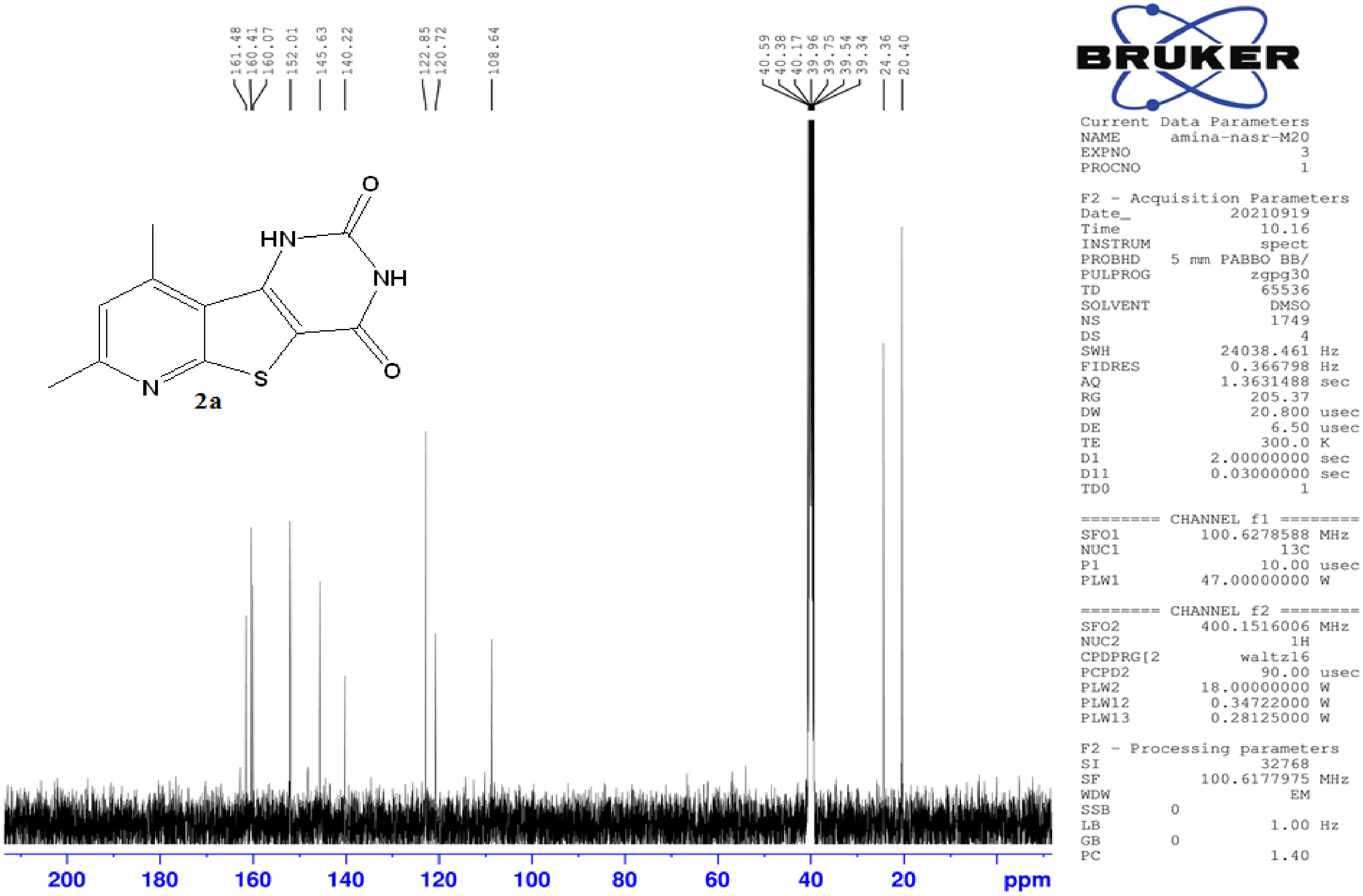
161.48
160.41
160.07
152.01
145.63
140.22
122.85
120.72
108.64
40.59
40.38
40.17
39.96
39.75
39.54
39.34
24.36
20.40
O
HN
NH
O
S
N
2a
BRUKER
Current Data Parameters
NAME amina-nasr-M20
EXPNO 3
PROCNO 1
F2 - Acquisition Parameters
Date_ 20210919
Time 10.16
INSTRUM spect
PROBHD 5 mm PABBO BB/
PULPROG zgpg30
TD 65536
SOLVENT DMSO
NS 1749
DS 4
SWH 24038.461 Hz
FIDRES 0.366798 Hz
AQ 1.3631488 sec
RG 205.37
DW 20.800 usec
DE 6.50 usec
TE 300.0 K
D1 2.00000000 sec
D11 0.03000000 sec
TD0 1
======== CHANNEL f1 ========
SFO1 100.6278588 MHz
NUC1 13C
P1 10.00 usec
PLW1 47.00000000 W
======== CHANNEL f2 ========
SFO2 400.1516006 MHz
NUC2 1H
CPDPRG[2 waltz16
PCPD2 90.00 usec
PLW2 18.00000000 W
PLW12 0.34722000 W
PLW13 0.28125000 W
F2 - Processing parameters
SI 32768
SF 100.6177975 MHz
WDW EM
SSB 0
LB 1.00 Hz
GB 0
PC 1.40
200
180
160
140
120
100
80
60
40
20
ppm

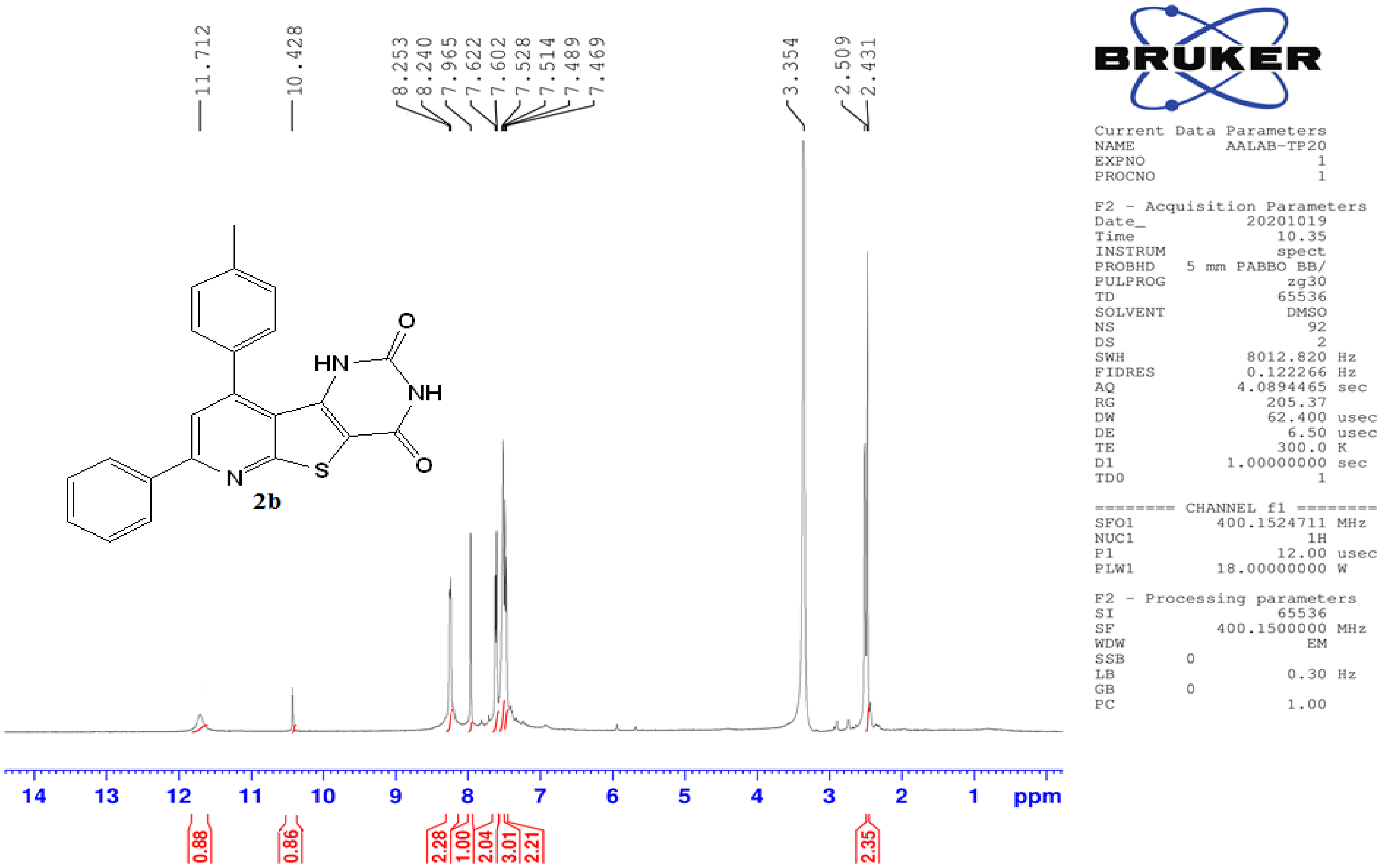

11.712
10.428
8.253
8.240
7.965
7.622
7.602
7.528
7.514
7.489
7.469
3.354
2.509
2.431
HN
NH
O
O
N
S
2b
BRUKER
Current Data Parameters
NAME AALAB-TP20
EXPNO 1
PROCNO 1
F2 - Acquisition Parameters
Date_ 20201019
Time 10.35
INSTRUM spect
PROBHD 5 mm PABBO BB/
PULPROG zg30
TD 65536
SOLVENT DMSO
NS 92
DS 2
SWH 8012.820 Hz
FIDRES 0.122266 Hz
AQ 4.0894465 sec
RG 205.37
DW 62.400 usec
DE 6.50 usec
TE 300.0 K
D1 1.00000000 sec
TD0 1
======== CHANNEL f1 ========
SFO1 400.1524711 MHz
NUC1 1H
P1 12.00 usec
PLW1 18.00000000 W
F2 - Processing parameters
SI 65536
SF 400.1500000 MHz
WDW EM
SSB 0
LB 0.30 Hz
GB 0
PC 1.00
14 13 12 11 10 9 8 7 6 5 4 3 2 1 ppm
0.88
0.86
2.28
1.00
2.04
3.01
2.21
2.35

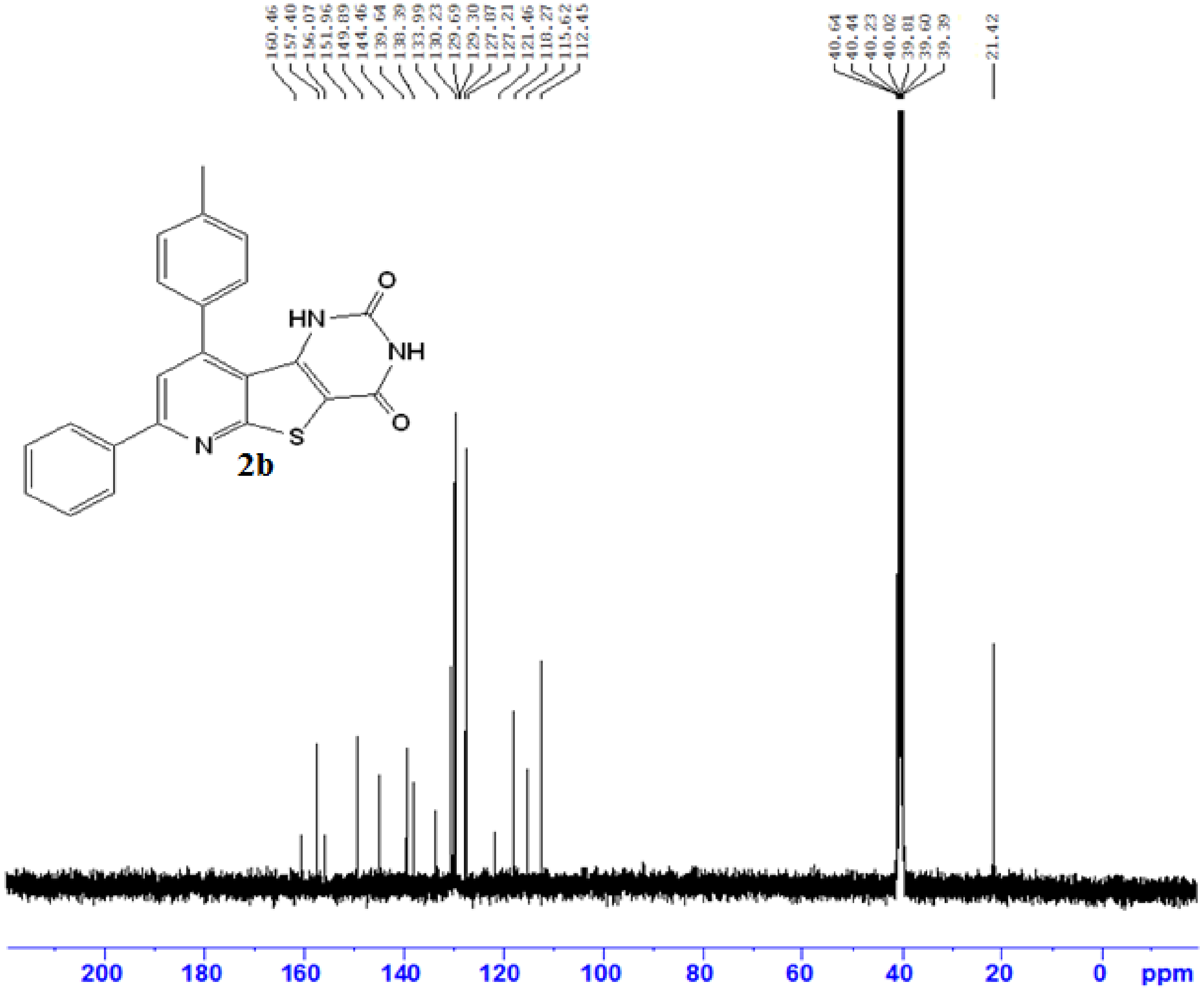

BRUKER

Current Data Parameters
NAME Eman-TP20
EXPNO 2
PROCNO 1

F2 - Acquisition Parameters
Date_ 20211205
Time 12.05
INSTRUM spect
PROBHD 5 mm PABBO BB/
PULPROG zgpg30
TD 65536
SOLVENT DMSO
NS 1098
DS 4
SWH 24038.461 Hz
FIDRES 0.366798 Hz
AQ 1.3631488 sec
RG 205.37
DW 20.800 usec
DE 6.50 usec
TE 300.0 K
D1 2.00000000 sec
D11 0.03000000 sec
TD0 1

======== CHANNEL f1 ========
SFO1 100.6278588 MHz
NUC1 13C
P1 10.00 usec
PLW1 47.00000000 W

======== CHANNEL f2 ========
SFO2 400.1516006 MHz
NUC2 1H
CPDPRG[2 waltz16
PCPD2 90.00 usec
PLW2 18.00000000 W
PLW12 0.34722000 W
PLW13 0.28125000 W

F2 - Processing parameters
SI 32768
SF 100.6177975 MHz
WDW EM
SSB 0
LB 1.00 Hz
GB 0
PC 1.40

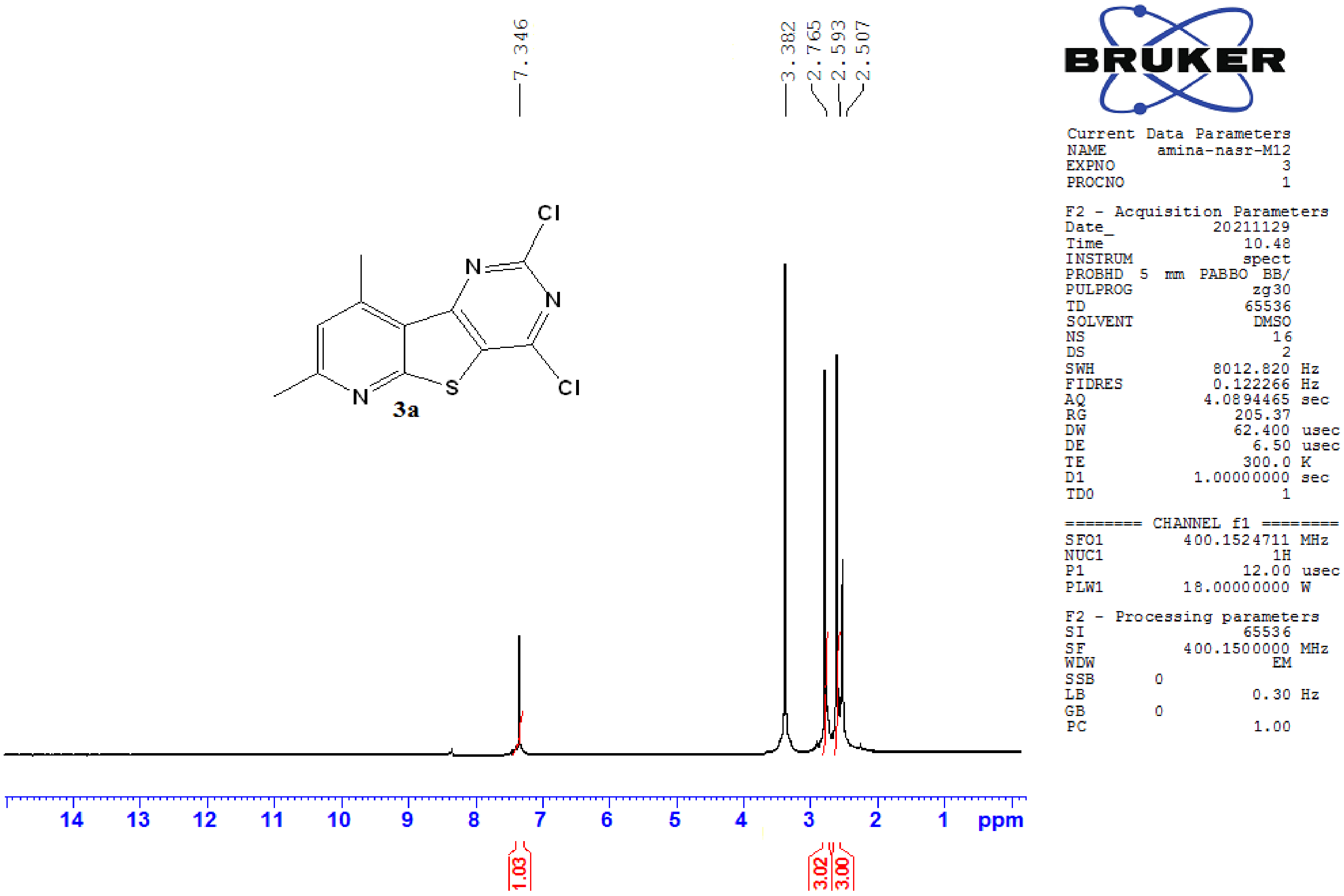
7.346
3.382
2.765
2.593
2.507
Cl
N
N
Cl
N
S
3a
BRUKER
Current Data Parameters
NAME amina-nasr-M12
EXPNO 3
PROCNO 1
F2 - Acquisition Parameters
Date_ 20211129
Time 10.48
INSTRUM spect
PROBHD 5 mm PABBO BB/
PULPROG zg30
TD 65536
SOLVENT DMSO
NS 16
DS 2
SWH 8012.820 Hz
FIDRES 0.122266 Hz
AQ 4.0894465 sec
RG 205.37
DW 62.400 usec
DE 6.50 usec
TE 300.0 K
D1 1.00000000 sec
TD0 1
======== CHANNEL f1 ========
SFO1 400.1524711 MHz
NUC1 1H
P1 12.00 usec
PLW1 18.00000000 W
F2 - Processing parameters
SI 65536
SF 400.1500000 MHz
WDW EM
SSB 0
LB 0.30 Hz
GB 0
PC 1.00
14 13 12 11 10 9 8 7 6 5 4 3 2 1 ppm
1.03
3.02
3.00

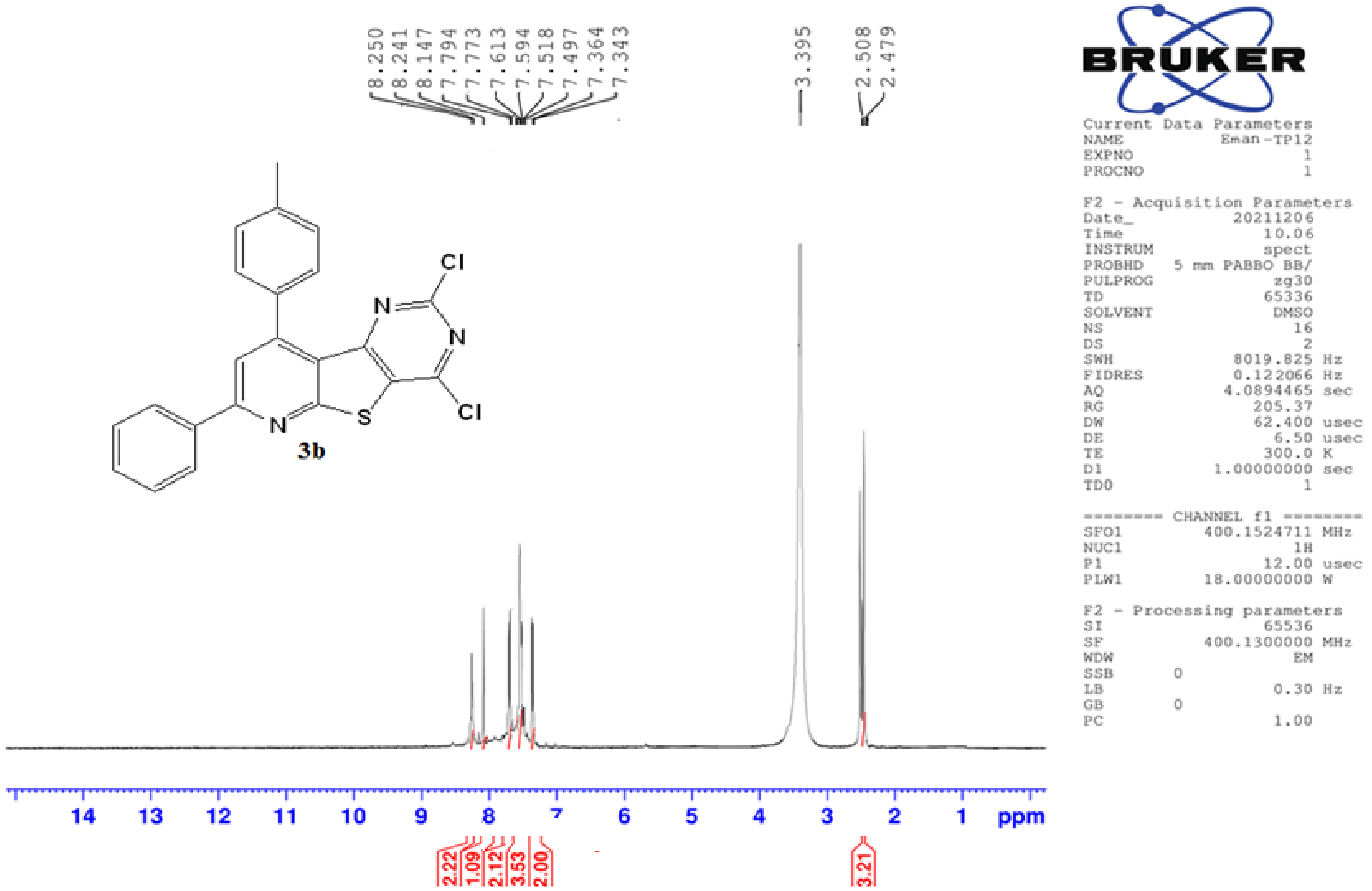
8.250
8.241
8.147
7.794
7.773
7.613
7.594
7.518
7.497
7.364
7.343
3.395
2.508
2.479
Cl
N
N
Cl
N
S
3b
BRUKER
Current Data Parameters
NAME Eman -TP12
EXPNO 1
PROCNO 1
F2 - Acquisition Parameters
Date_ 20211206
Time 10.06
INSTRUM spect
PROBHD 5 mm PABBO BB/
PULPROG zg30
TD 65336
SOLVENT DMSO
NS 16
DS 2
SWH 8019.825 Hz
FIDRES 0.122066 Hz
AQ 4.0894465 sec
RG 205.37
DW 62.400 usec
DE 6.50 usec
TE 300.0 K
D1 1.00000000 sec
TD0 1
======== CHANNEL f1 ========
SFO1 400.1524711 MHz
NUC1 1H
P1 12.00 usec
PLW1 18.00000000 W
F2 - Processing parameters
SI 65536
SF 400.1300000 MHz
WDW EM
SSB 0
LB 0.30 Hz
GB 0
PC 1.00
14 13 12 11 10 9 8 7 6 5 4 3 2 1 ppm
2.22
1.09
2.12
3.53
2.00
3.21

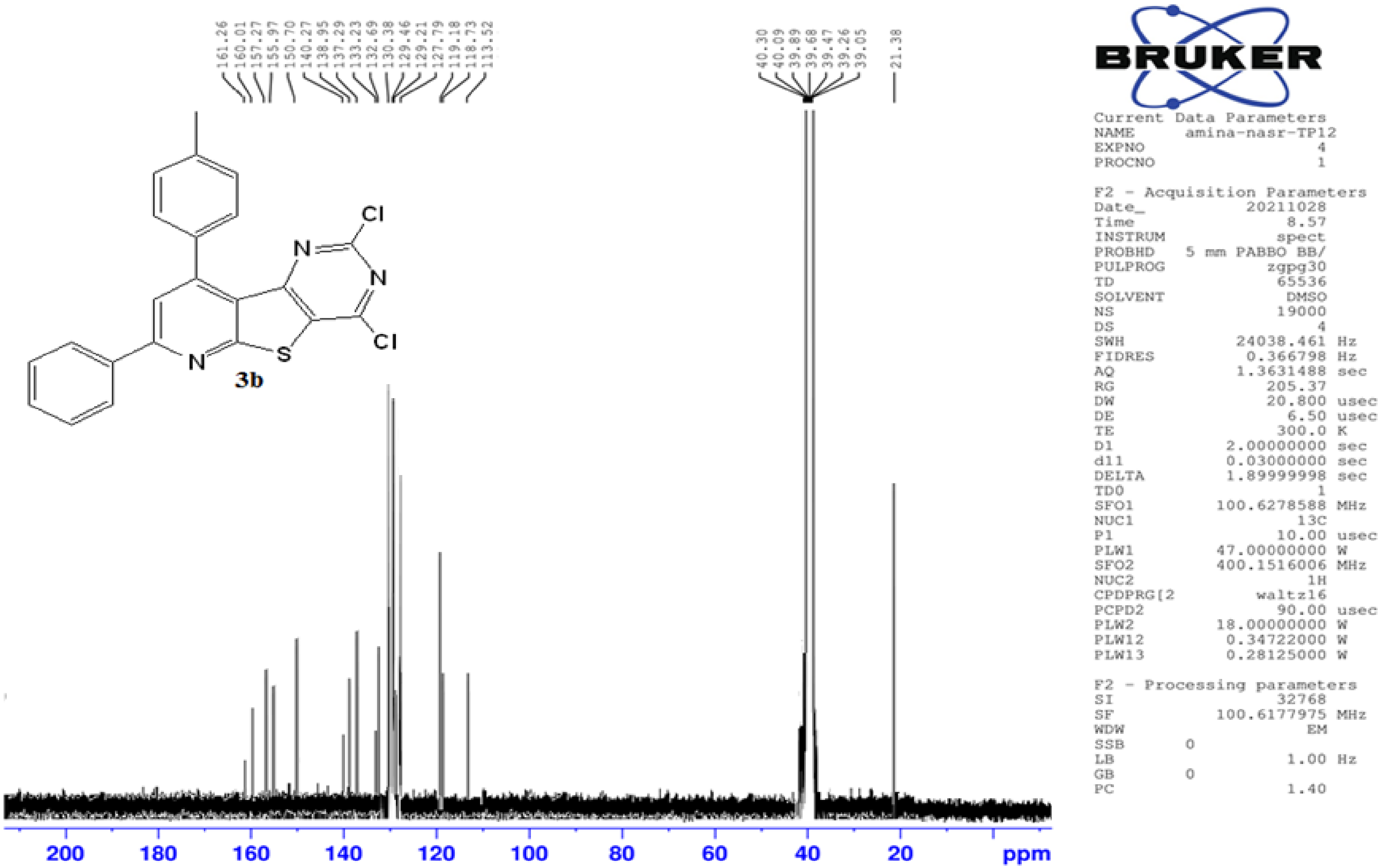
161.26
160.01
157.27
155.97
150.70
140.27
138.95
137.29
133.23
132.69
130.38
129.46
129.21
127.79
119.18
118.73
113.52
40.30
40.09
39.89
39.68
39.47
39.26
39.05
21.38
Cl
N
N
Cl
N
S
3b
BRUKER
Current Data Parameters
NAME amina-nasr-TP12
EXPNO 4
PROCNO 1
F2 - Acquisition Parameters
Date_ 20211028
Time 8.57
INSTRUM spect
PROBHD 5 mm PABBO BB/
PULPROG zgpg30
TD 65536
SOLVENT DMSO
NS 19000
DS 4
SWH 24038.461 Hz
FIDRES 0.366798 Hz
AQ 1.3631488 sec
RG 205.37
DW 20.800 usec
DE 6.50 usec
TE 300.0 K
D1 2.00000000 sec
d11 0.03000000 sec
DELTA 1.89999998 sec
TD0 1
SFO1 100.6278588 MHz
NUC1 13C
P1 10.00 usec
PLW1 47.00000000 W
SFO2 400.1516006 MHz
NUC2 1H
CPDPRG[2 waltz16
PCPD2 90.00 usec
PLW2 18.00000000 W
PLW12 0.34722000 W
PLW13 0.28125000 W
F2 - Processing parameters
SI 32768
SF 100.6177975 MHz
WDW EM
SSB 0
LB 1.00 Hz
GB 0
PC 1.40
200
180
160
140
120
100
80
60
40
20
ppm

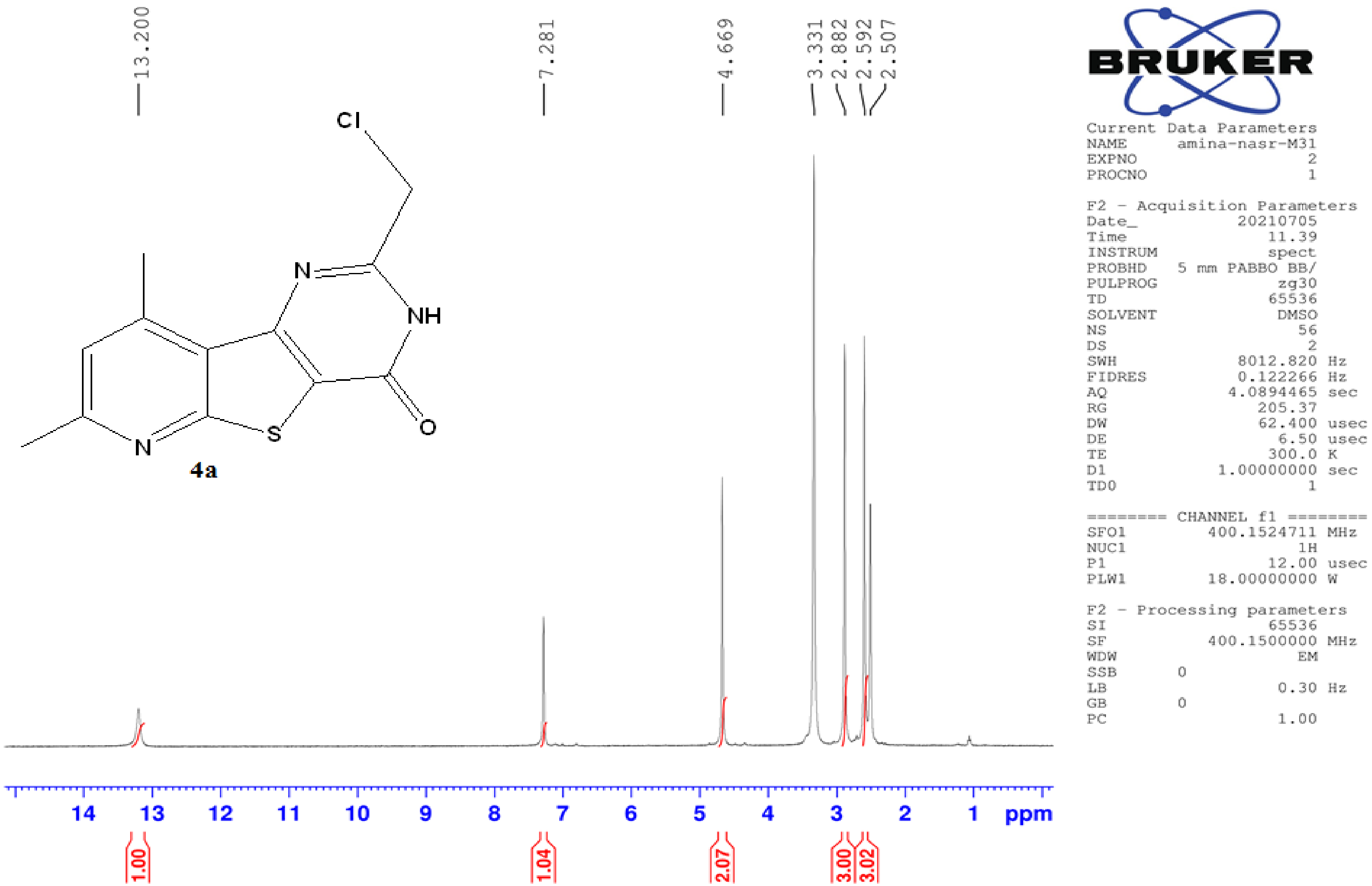
13.200
7.281
4.669
3.331
2.882
2.592
2.507
Cl
N
NH
N
S
O
4a
14
13
12
11
10
9
8
7
6
5
4
3
2
1
ppm
1.00
1.04
2.07
3.00
3.02
BRUKER
Current Data Parameters
NAME amina-nasr-M31
EXPNO 2
PROCNO 1
F2 - Acquisition Parameters
Date_ 20210705
Time 11.39
INSTRUM spect
PROBHD 5 mm PABBO BB/
PULPROG zg30
TD 65536
SOLVENT DMSO
NS 56
DS 2
SWH 8012.820 Hz
FIDRES 0.122266 Hz
AQ 4.0894465 sec
RG 205.37
DW 62.400 usec
DE 6.50 usec
TE 300.0 K
D1 1.00000000 sec
TD0 1
======== CHANNEL f1 ========
SFO1 400.1524711 MHz
NUC1 1H
P1 12.00 usec
PLW1 18.00000000 W
F2 - Processing parameters
SI 65536
SF 400.1500000 MHz
WDW EM
SSB 0
LB 0.30 Hz
GB 0
PC 1.00

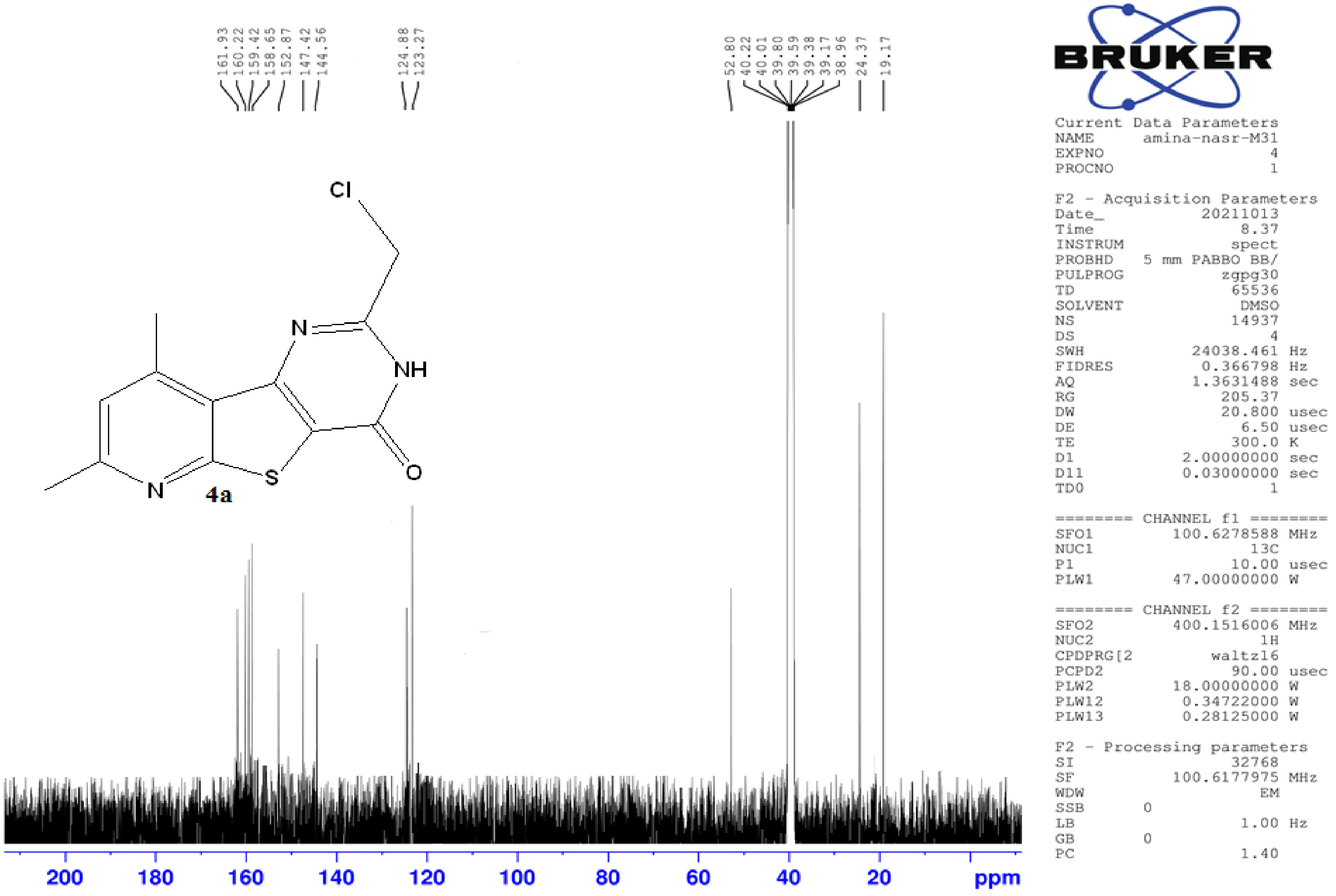
161.93
160.22
159.42
158.65
152.87
147.42
144.56
124.88
123.27
52.80
40.22
40.01
39.80
39.59
39.38
39.17
38.96
24.37
19.17
Cl
N
NH
N
S
O
4a
200
180
160
140
120
100
80
60
40
20
ppm
BRUKER
Current Data Parameters
NAME amina-nasr-M31
EXPNO 4
PROCNO 1
F2 - Acquisition Parameters
Date_ 20211013
Time 8.37
INSTRUM spect
PROBHD 5 mm PABBO BB/
PULPROG zgpg30
TD 65536
SOLVENT DMSO
NS 14937
DS 4
SWH 24038.461 Hz
FIDRES 0.366798 Hz
AQ 1.3631488 sec
RG 205.37
DW 20.800 usec
DE 6.50 usec
TE 300.0 K
D1 2.00000000 sec
D11 0.03000000 sec
TD0 1
======== CHANNEL f1 ========
SFO1 100.6278588 MHz
NUC1 13C
P1 10.00 usec
PLW1 47.00000000 W
======== CHANNEL f2 ========
SFO2 400.1516006 MHz
NUC2 1H
CPDPRG[2 waltz16
PCPD2 90.00 usec
PLW2 18.00000000 W
PLW12 0.34722000 W
PLW13 0.28125000 W
F2 - Processing parameters
SI 32768
SF 100.6177975 MHz
WDW EM
SSB 0
LB 1.00 Hz
GB 0
PC 1.40

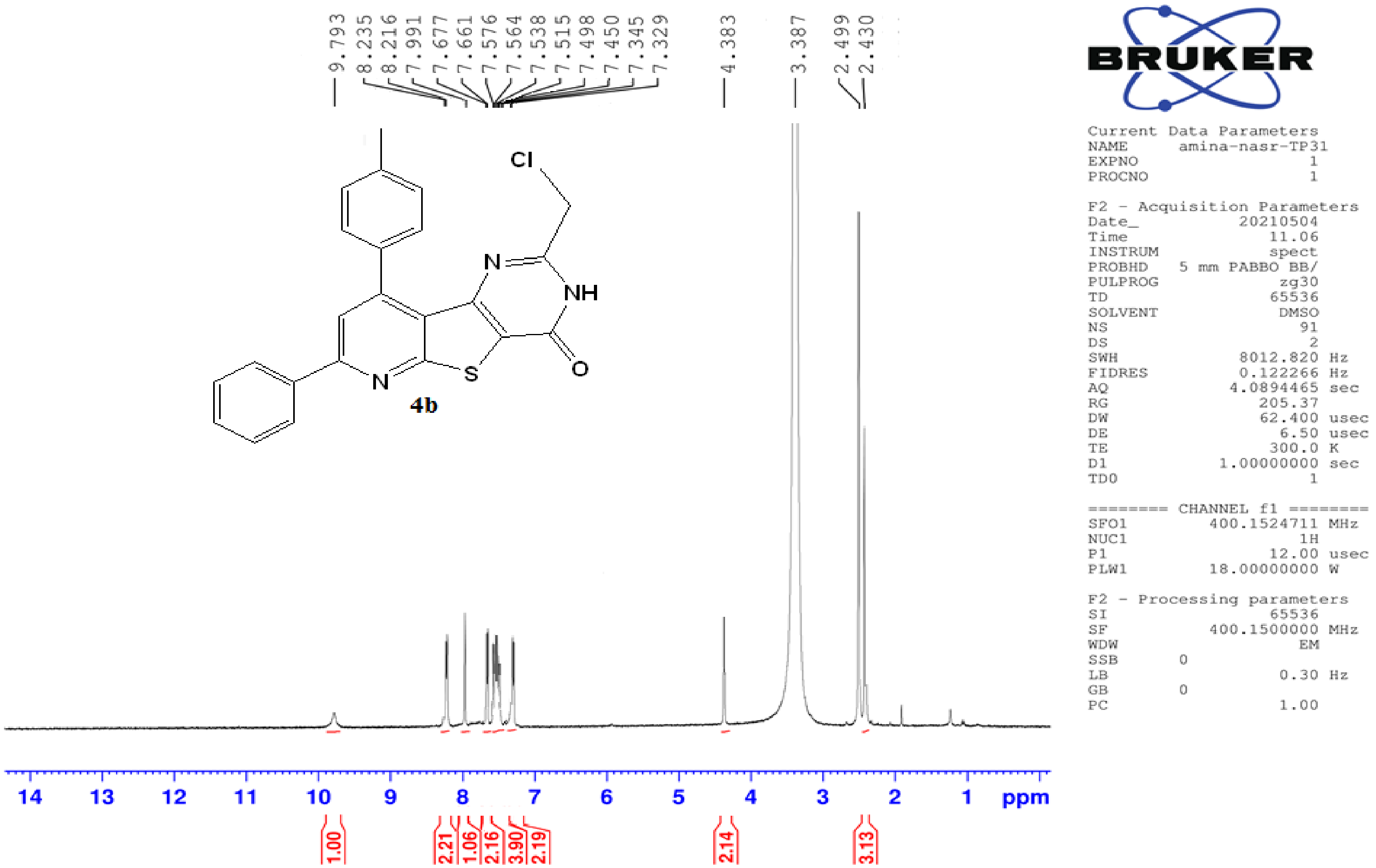
9.793
8.235
8.216
7.991
7.677
7.661
7.576
7.564
7.538
7.515
7.498
7.450
7.345
7.329
4.383
3.387
2.499
2.430
Cl
N
NH
S
O
N
4b
14 13 12 11 10 9 8 7 6 5 4 3 2 1 ppm
1.00
2.21
1.06
2.16
3.90
2.19
2.14
3.13
BRUKER
Current Data Parameters
NAME amina-nasr-TP31
EXPNO 1
PROCNO 1
F2 - Acquisition Parameters
Date_ 20210504
Time 11.06
INSTRUM spect
PROBHD 5 mm PABBO BB/
PULPROG zg30
TD 65536
SOLVENT DMSO
NS 91
DS 2
SWH 8012.820 Hz
FIDRES 0.122266 Hz
AQ 4.0894465 sec
RG 205.37
DW 62.400 usec
DE 6.50 usec
TE 300.0 K
D1 1.00000000 sec
TD0 1
======== CHANNEL f1 ========
SFO1 400.1524711 MHz
NUC1 1H
P1 12.00 usec
PLW1 18.00000000 W
F2 - Processing parameters
SI 65536
SF 400.1500000 MHz
WDW EM
SSB 0
LB 0.30 Hz
GB 0
PC 1.00

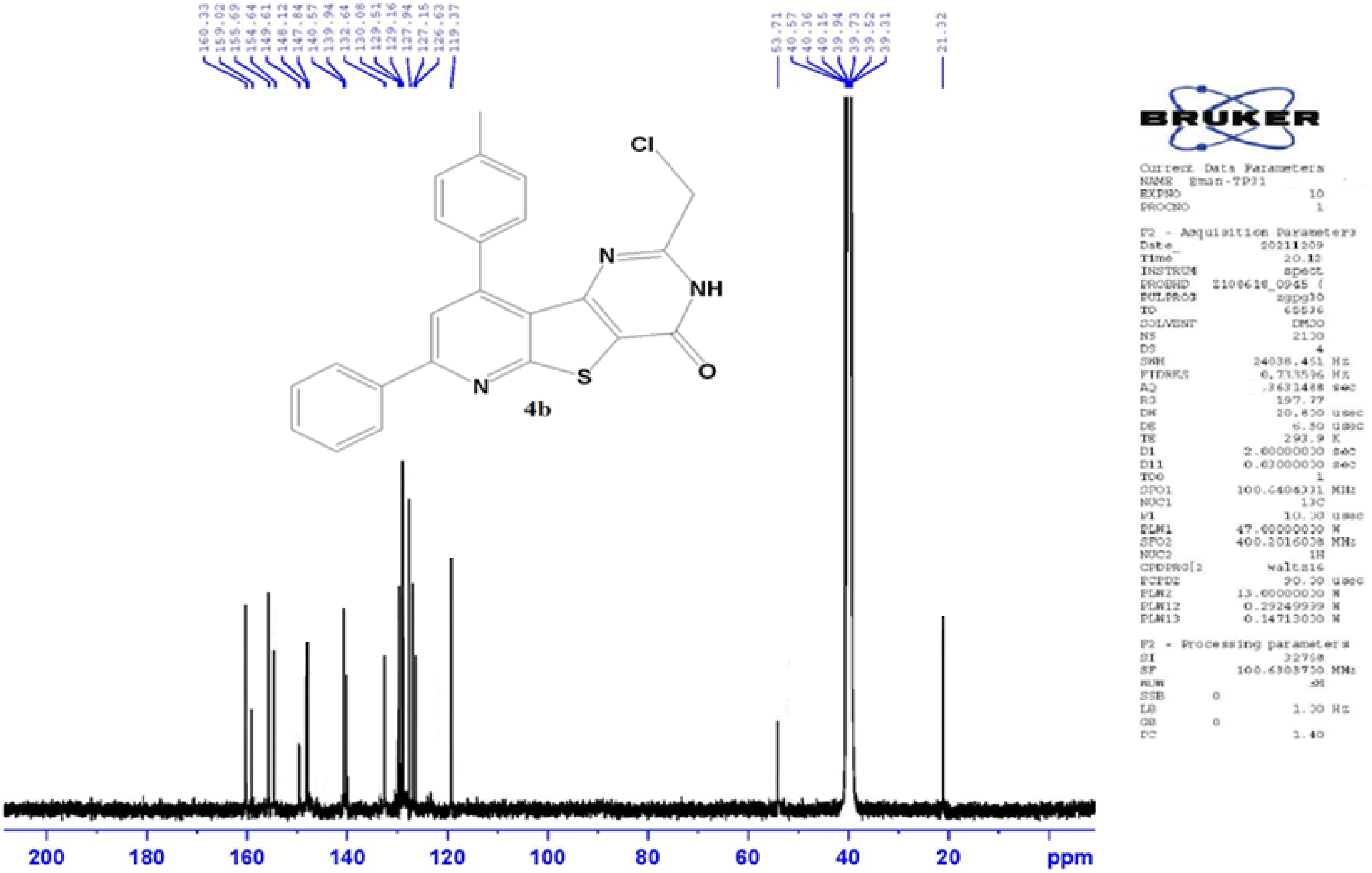
160.33
159.02
155.69
154.64
149.61
148.12
147.84
140.57
139.94
132.64
130.08
129.51
129.16
127.94
127.15
126.63
119.37
53.71
40.57
40.36
40.15
39.94
39.73
39.52
39.31
21.32
Cl
N
NH
O
S
N
4b
BRUKER
Current Data Parameters
EXPNO 10
PROCNO 1
F2 - Acquisition Parameters
Time 20.12
INSTRUM spect
SOLVENT DMSO
DS 4
RG 197.77
TE 293.9 K
D1 2.00000000 sec
TD0 1
NUC1 13C
PLW1 47.00000000 W
SFO2 400.2016008 MHz
NUC2 1H
PLW2 13.00000000 W
PLW12 0.29249999 W
PLW13 0.14713000 W
F2 - Processing parameters
SSB 0
LB 1.00 Hz
GB 0
PC 1.40
200
180
160
140
120
100
80
60
40
20
ppm

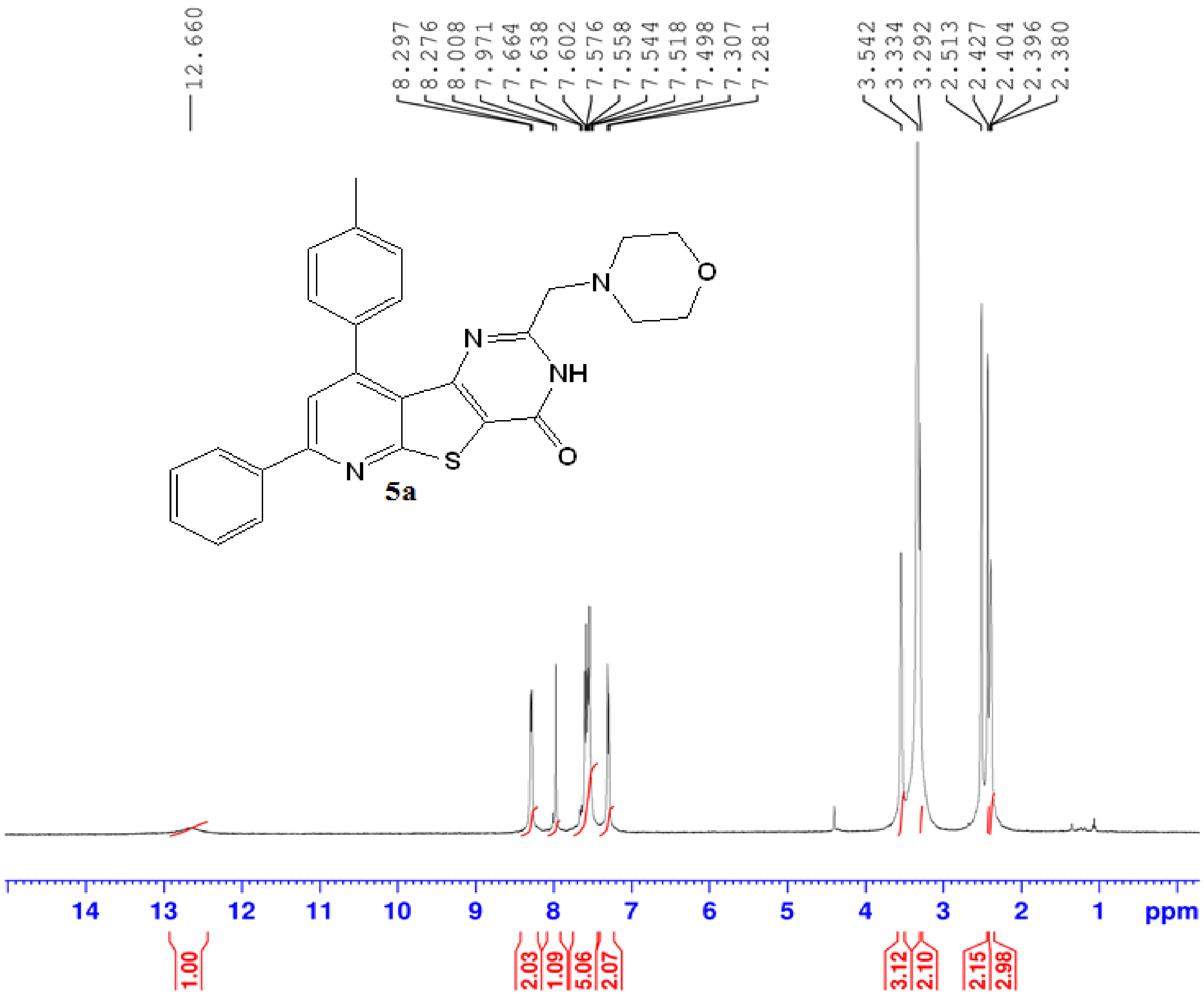

BRUKER

```
Current Data Parameters
NAME       amina-nasr-TP32
EXPNO                    1
PROCNO                   1

F2 - Acquisition Parameters
Date_             20210415
Time                 12.09
INSTRUM              spect
PROBHD     5 mm PABBO BB/
PULPROG               zg30
TD                   65536
SOLVENT               DMSO
NS                     128
DS                       2
SWH               8012.820 Hz
FIDRES            0.122266 Hz
AQ               4.0894465 sec
RG                  205.37
DW                  62.400 usec
DE                    6.50 usec
TE                   300.0 K
D1              1.00000000 sec
TD0                      1

======== CHANNEL f1 ========
SFO1         400.1524711 MHz
NUC1                  1H
P1                 12.00 usec
PLW1         18.00000000 W

F2 - Processing parameters
SI                   65536
SF           400.1500000 MHz
WDW                     EM
SSB      0
LB                    0.30 Hz
GB       0
PC                    1.00
```

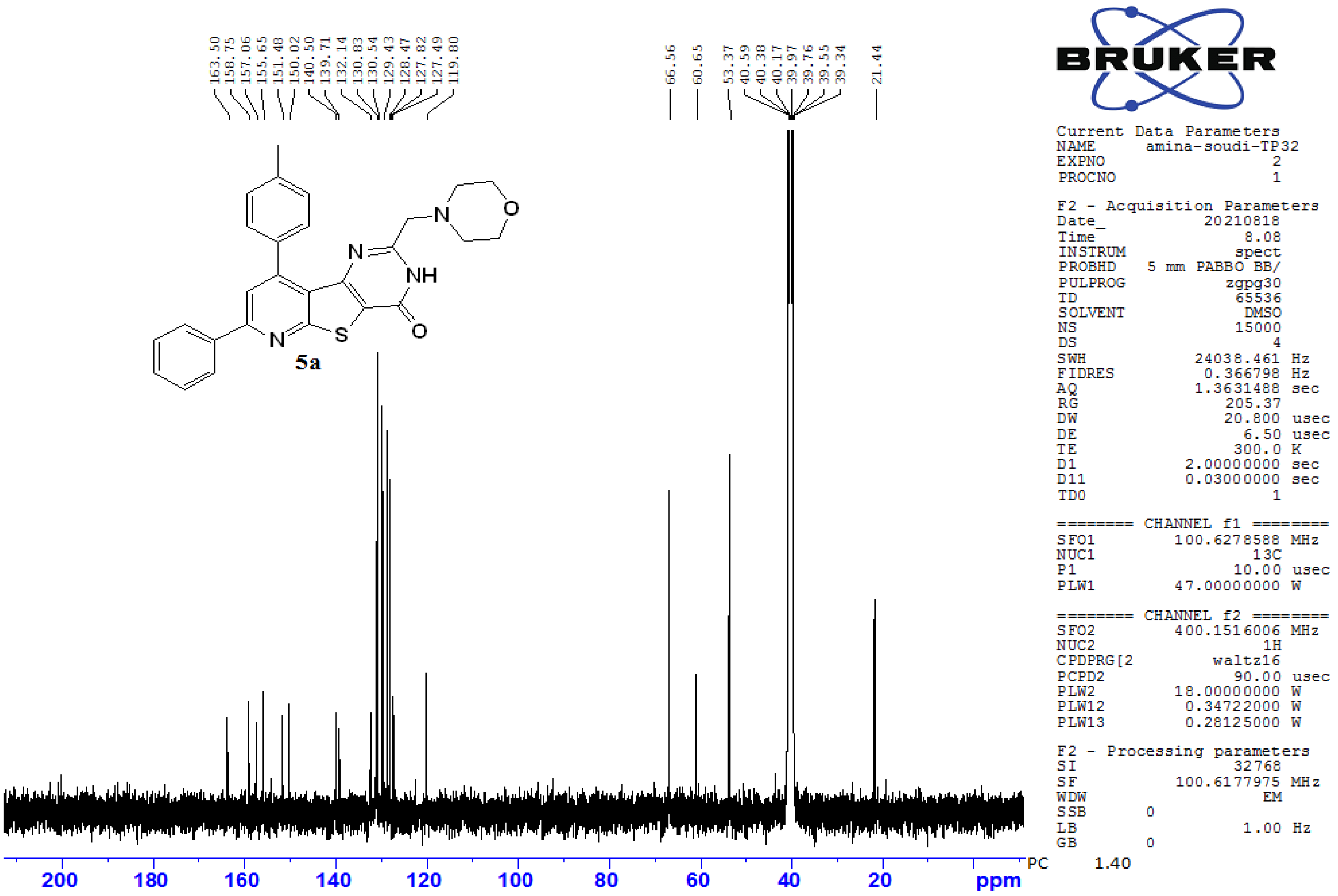
163.50
158.75
157.06
155.65
151.48
150.02
140.50
139.71
132.14
130.83
130.54
129.43
128.47
127.82
127.49
119.80
66.56
60.65
53.37
40.59
40.38
40.17
39.97
39.76
39.55
39.34
21.44
N
NH
O
S
N
N
O
5a
BRUKER
Current Data Parameters
NAME amina-soudi-TP32
EXPNO 2
PROCNO 1
F2 - Acquisition Parameters
Date_ 20210818
Time 8.08
INSTRUM spect
PROBHD 5 mm PABBO BB/
PULPROG zgpg30
TD 65536
SOLVENT DMSO
NS 15000
DS 4
SWH 24038.461 Hz
FIDRES 0.366798 Hz
AQ 1.3631488 sec
RG 205.37
DW 20.800 usec
DE 6.50 usec
TE 300.0 K
D1 2.00000000 sec
D11 0.03000000 sec
TD0 1
======== CHANNEL f1 ========
SFO1 100.6278588 MHz
NUC1 13C
P1 10.00 usec
PLW1 47.00000000 W
======== CHANNEL f2 ========
SFO2 400.1516006 MHz
NUC2 1H
CPDPRG[2 waltz16
PCPD2 90.00 usec
PLW2 18.00000000 W
PLW12 0.34722000 W
PLW13 0.28125000 W
F2 - Processing parameters
SI 32768
SF 100.6177975 MHz
WDW EM
SSB 0
LB 1.00 Hz
GB 0
PC 1.40
200
180
160
140
120
100
80
60
40
20
ppm

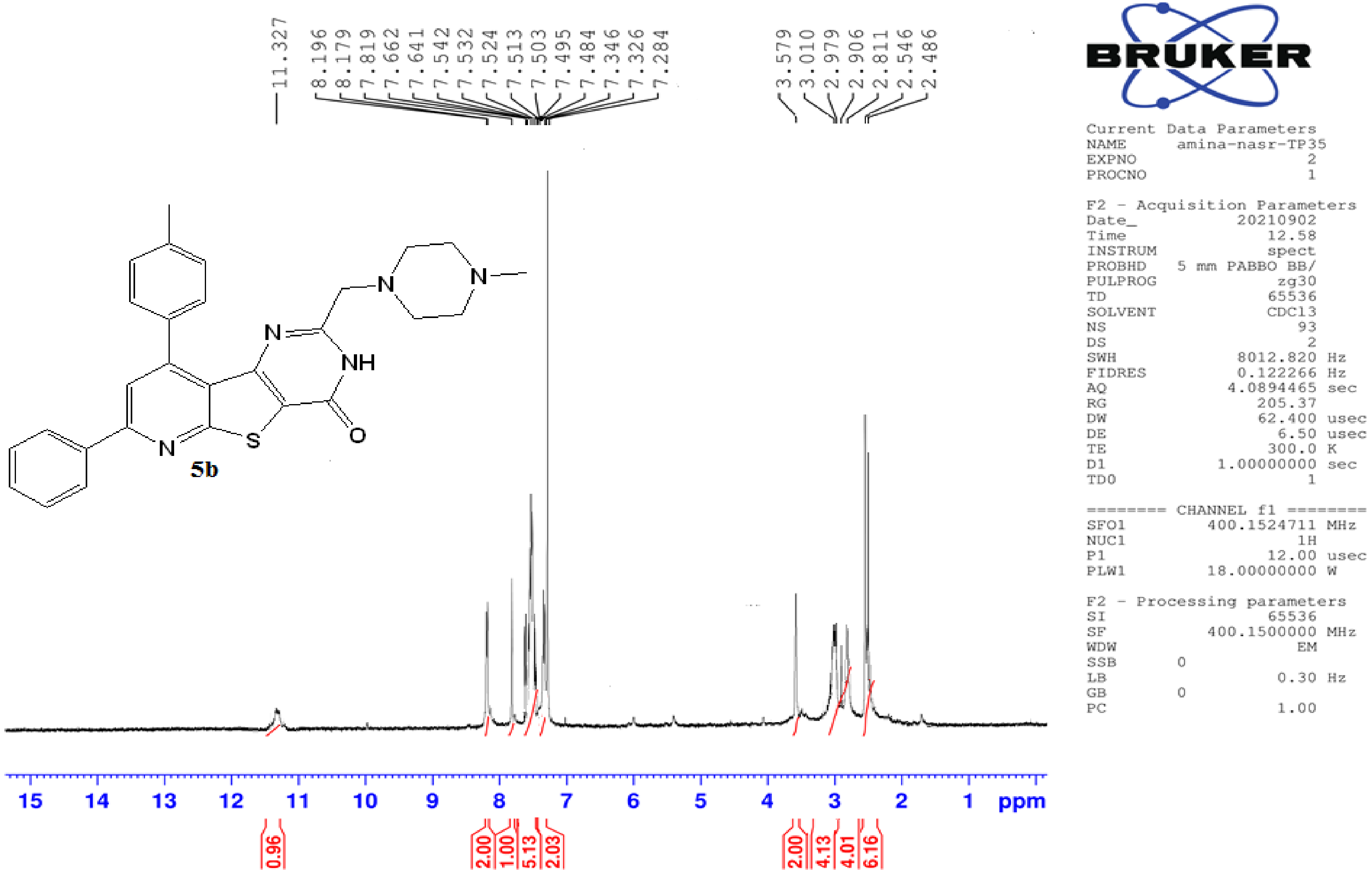
11.327
8.196
8.179
7.819
7.662
7.641
7.542
7.532
7.524
7.513
7.503
7.495
7.484
7.346
7.326
7.284
3.579
3.010
2.979
2.906
2.811
2.546
2.486
N
NH
N
N
O
S
N
5b
15 14 13 12 11 10 9 8 7 6 5 4 3 2 1 ppm
0.96
2.00
1.00
5.13
2.03
2.00
4.13
4.01
6.16
Current Data Parameters
NAME amina-nasr-TP35
EXPNO 2
PROCNO 1
F2 - Acquisition Parameters
Date_ 20210902
Time 12.58
INSTRUM spect
PROBHD 5 mm PABBO BB/
PULPROG zg30
TD 65536
SOLVENT CDCl3
NS 93
DS 2
SWH 8012.820 Hz
FIDRES 0.122266 Hz
AQ 4.0894465 sec
RG 205.37
DW 62.400 usec
DE 6.50 usec
TE 300.0 K
D1 1.00000000 sec
TD0 1
======== CHANNEL f1 ========
SFO1 400.1524711 MHz
NUC1 1H
P1 12.00 usec
PLW1 18.00000000 W
F2 - Processing parameters
SI 65536
SF 400.1500000 MHz
WDW EM
SSB 0
LB 0.30 Hz
GB 0
PC 1.00

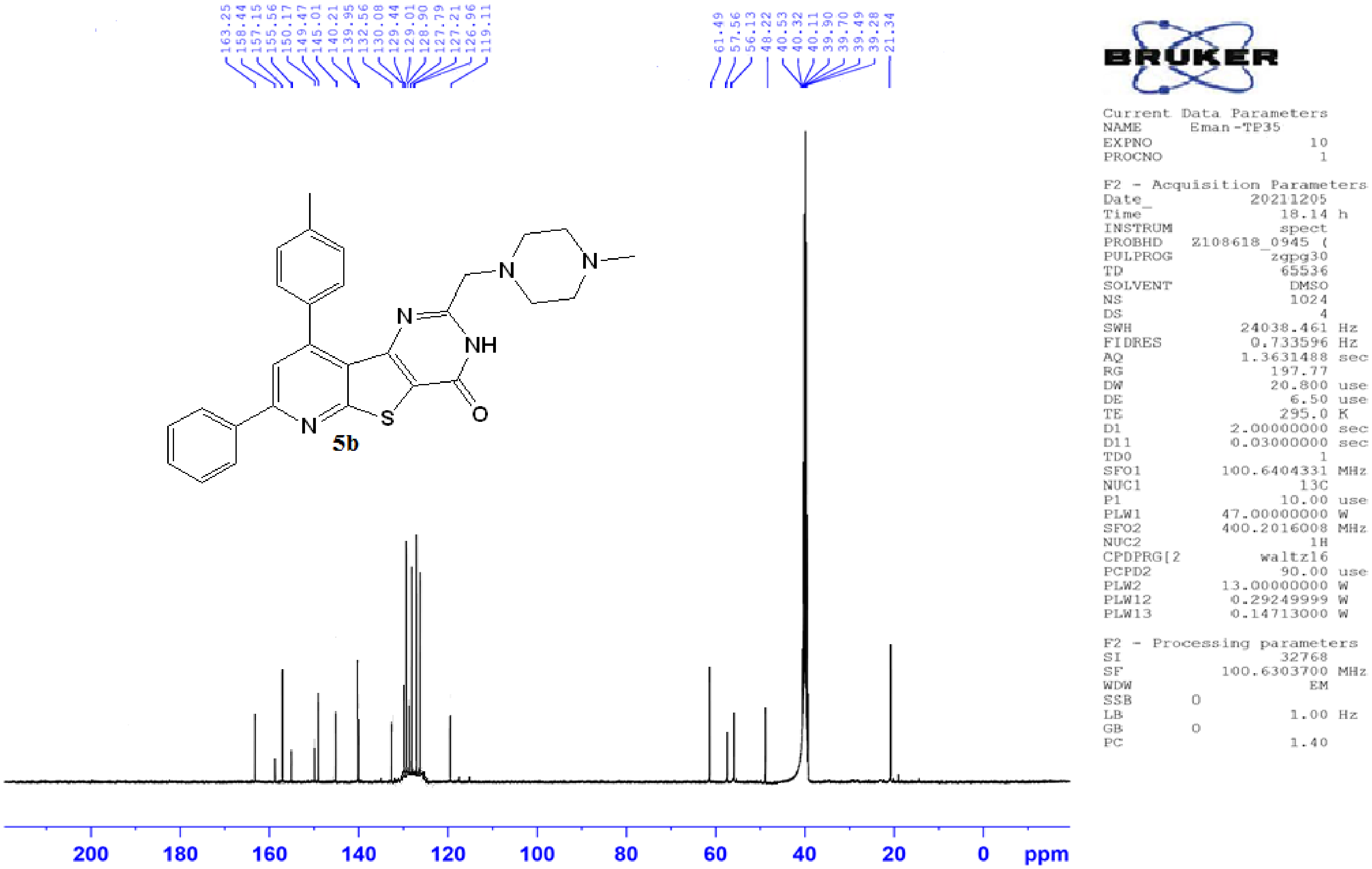
163.25
158.44
157.15
155.56
150.17
149.47
145.01
140.21
139.95
132.56
130.08
129.44
129.01
128.90
127.79
127.21
126.96
119.11
61.49
57.56
56.13
48.22
40.53
40.32
40.11
39.90
39.70
39.49
39.28
21.34
N
NH
N
N
O
S
N
5b
200
180
160
140
120
100
80
60
40
20
0
ppm
BRUKER
Current Data Parameters
NAME Eman-TP35
EXPNO 10
PROCNO 1
F2 - Acquisition Parameters
Date_ 20211205
Time 18.14 h
INSTRUM spect
PROBHD Z108618_0945 (
PULPROG zgpg30
TD 65536
SOLVENT DMSO
NS 1024
DS 4
SWH 24038.461 Hz
FIDRES 0.733596 Hz
AQ 1.3631488 sec
RG 197.77
DW 20.800 use
DE 6.50 use
TE 295.0 K
D1 2.00000000 sec
D11 0.03000000 sec
TD0 1
SFO1 100.6404331 MHz
NUC1 13C
P1 10.00 use
PLW1 47.00000000 W
SFO2 400.2016008 MHz
NUC2 1H
CPDPRG[2 waltz16
PCPD2 90.00 use
PLW2 13.00000000 W
PLW12 0.29249999 W
PLW13 0.14713000 W
F2 - Processing parameters
SI 32768
SF 100.6303700 MHz
WDW EM
SSB 0
LB 1.00 Hz
GB 0
PC 1.40

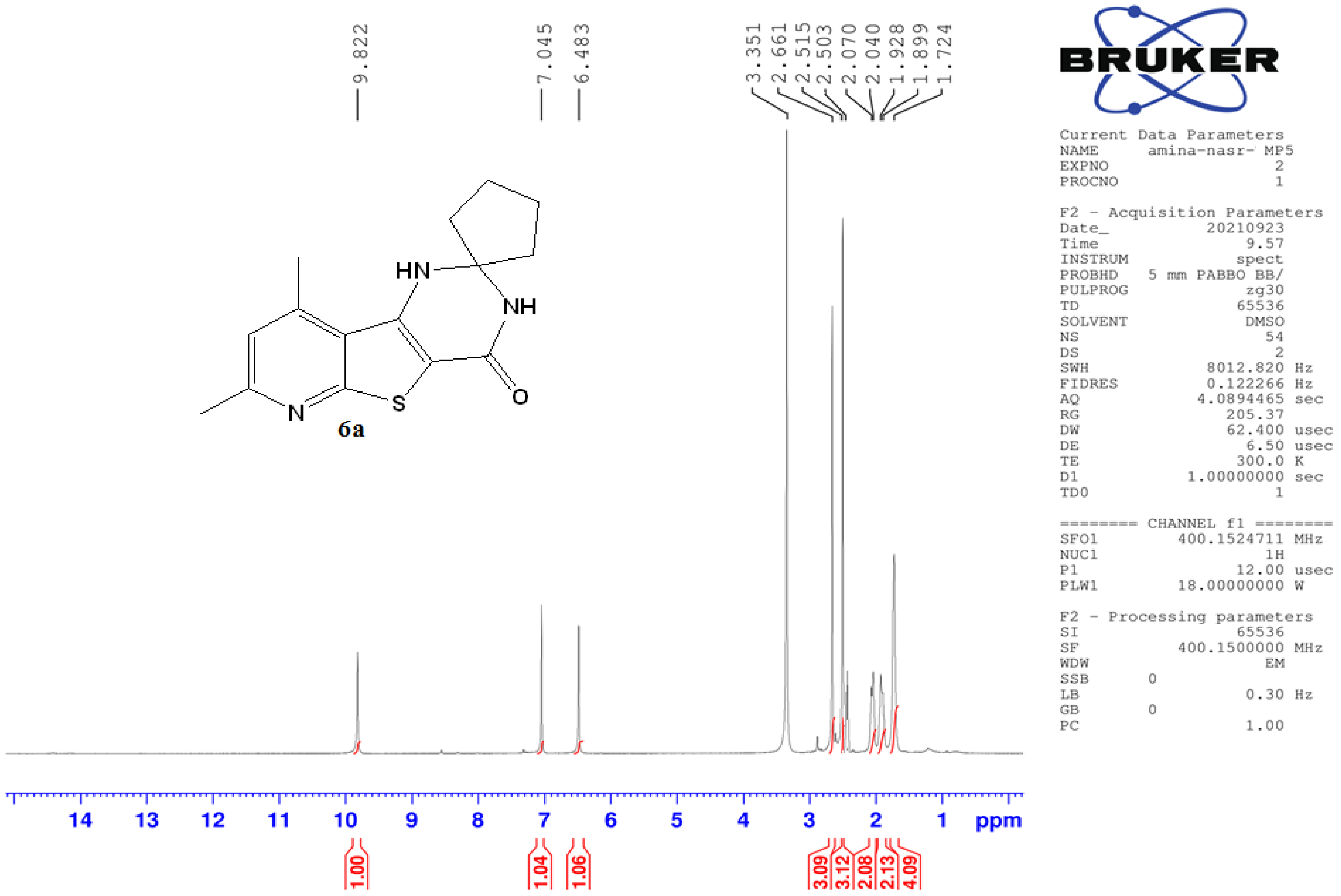
9.822
7.045
6.483
3.351
2.661
2.515
2.503
2.070
2.040
1.928
1.899
1.724
HN
NH
O
S
N
6a
14
13
12
11
10
9
8
7
6
5
4
3
2
1
ppm
1.00
1.04
1.06
3.09
3.12
2.08
2.13
4.09
BRUKER
Current Data Parameters
NAME amina-nasr- MP5
EXPNO 2
PROCNO 1
F2 - Acquisition Parameters
Date_ 20210923
Time 9.57
INSTRUM spect
PROBHD 5 mm PABBO BB/
PULPROG zg30
TD 65536
SOLVENT DMSO
NS 54
DS 2
SWH 8012.820 Hz
FIDRES 0.122266 Hz
AQ 4.0894465 sec
RG 205.37
DW 62.400 usec
DE 6.50 usec
TE 300.0 K
D1 1.00000000 sec
TD0 1
======== CHANNEL f1 ========
SFO1 400.1524711 MHz
NUC1 1H
P1 12.00 usec
PLW1 18.00000000 W
F2 - Processing parameters
SI 65536
SF 400.1500000 MHz
WDW EM
SSB 0
LB 0.30 Hz
GB 0
PC 1.00

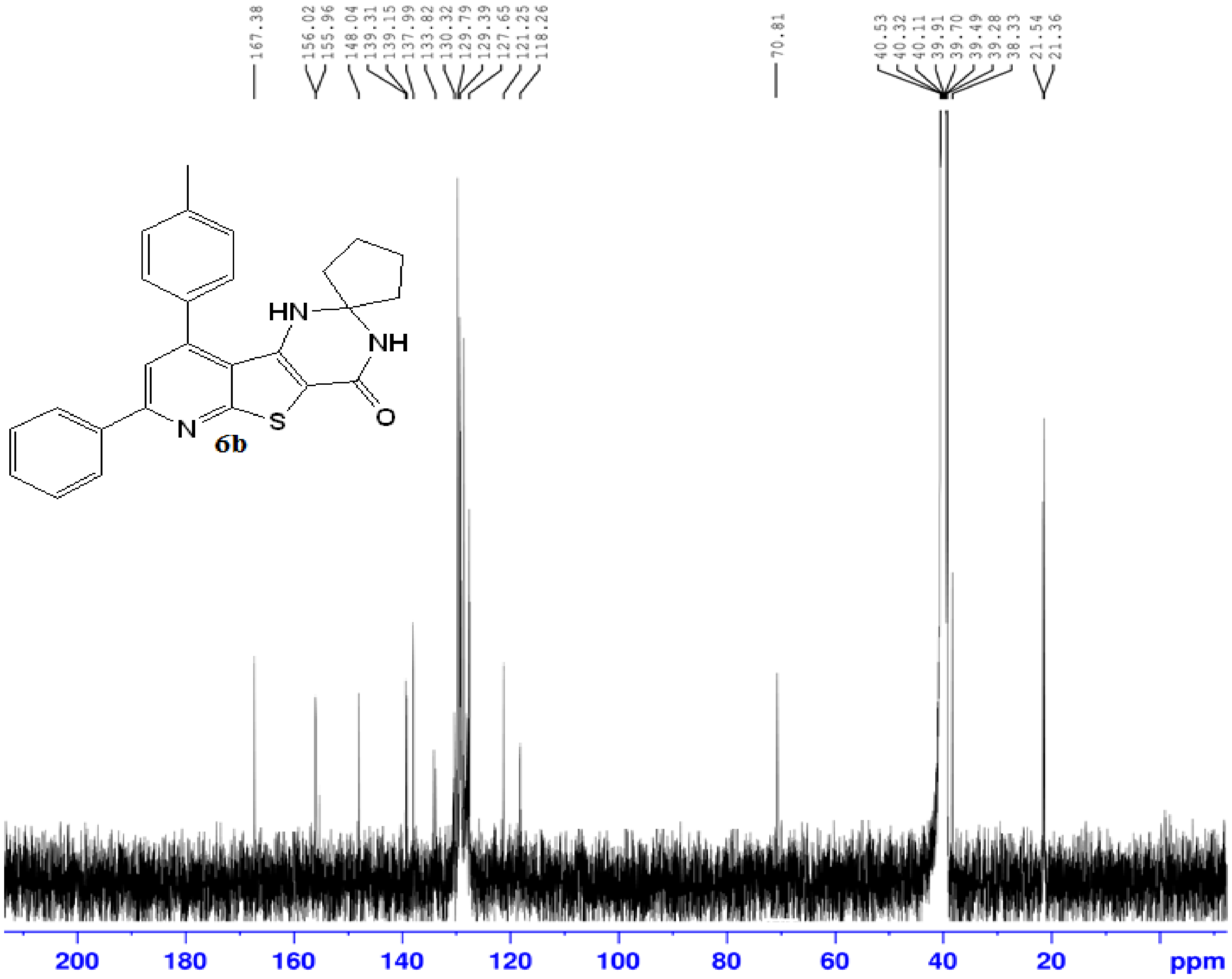

Current Data Parameters
NAME amina-nasr-TPP5
EXPNO 4
PROCNO 1

F2 - Acquisition Parameters
Date_ 20211019
Time 7.37
INSTRUM spect
PROBHD 5 mm PABBO BB/
PULPROG zgpg30
TD 65536
SOLVENT DMSO
NS 15000
DS 4
SWH 24038.461 Hz
FIDRES 0.366798 Hz
AQ 1.3631488 sec
RG 205.37
DW 20.800 usec
DE 6.50 usec
TE 300.0 K
D1 2.00000000 sec
D11 0.03000000 sec
TD0 1

======== CHANNEL f1 ========
SFO1 100.6278588 MHz
NUC1 13C
P1 10.00 usec
PLW1 47.00000000 W

======== CHANNEL f2 ========
SFO2 400.1516006 MHz
NUC2 1H
CPDPRG[2 waltz16
PCPD2 90.00 usec
PLW2 18.00000000 W
PLW12 0.34722000 W
PLW13 0.28125000 W

F2 - Processing parameters
SI 32768
SF 100.6177975 MHz
WDW EM
SSB 0
LB 1.00 Hz
GB 0
PC 1.40

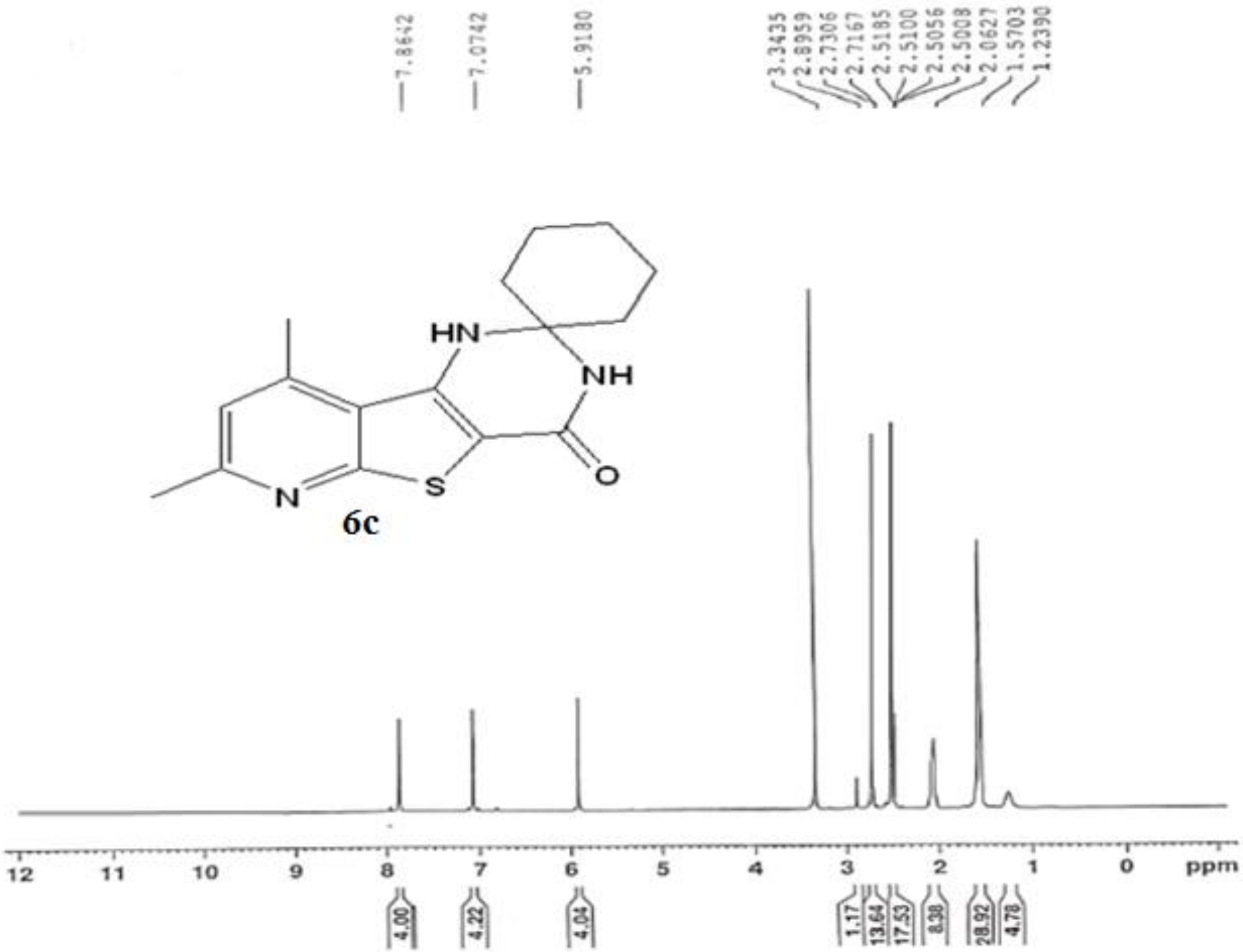

Current Data Parameters

NAME	amina-nasr- M5
EXPNO	1
PROCNO	1

F2 - Acquisition Parameters

Date_	20170913
Time	9.31
INSTRUM	spect
PROBHD	5 mm PABBO BB/
PULPROG	zg30
TD	65536
SOLVENT	DMSO
NS	121
DS	2
SWH	8012.820 Hz
FIDRES	0.122266 Hz
AQ	4.0894465 sec
RG	205.37
DW	62.400 usec
DE	6.50 usec
TE	300.0 K
D1	1.00000000 sec
TD0	1

======== CHANNEL f1 ========

SFO1	400.1524711 MHz
NUC1	1H
P1	12.00 usec
PLW1	18.00000000 W

F2 - Processing parameters

SI	65536
SF	400.1500000 MHz
WDW	EM
SSB	0
LB	0.30 Hz

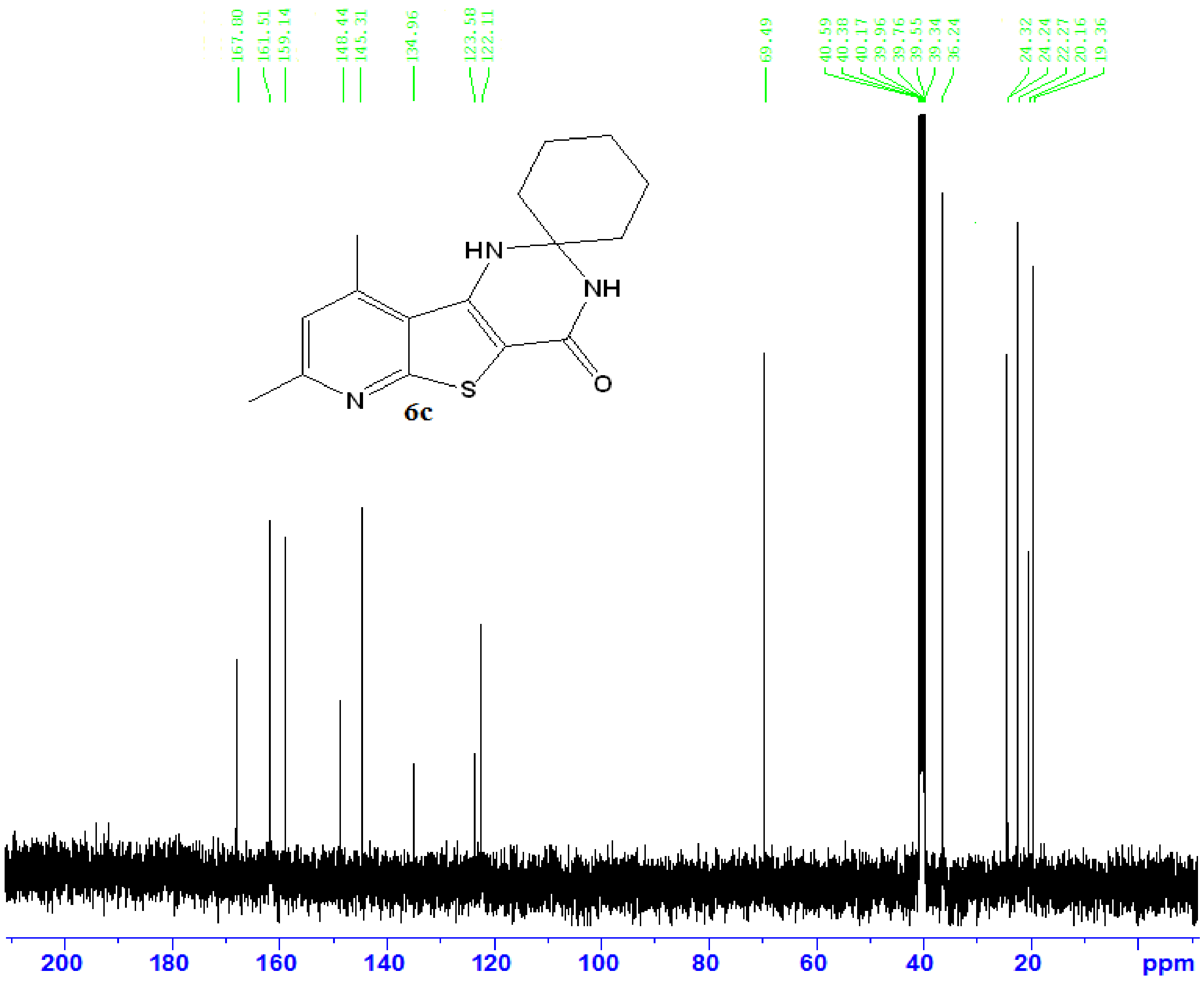


```
Current Data Parameters
NAME        amina-nasr-M5
EXPNO                   1
PROCNO                  1

F2 - Acquisition Parameters
Date_            20180919
Time                10.21
INSTRUM             spect
PROBHD   5 mm PABBO BB/
PULPROG           zgpg30
TD                  65536
SOLVENT              DMSO
NS                    603
DS                      4
SWH             24038.461 Hz
FIDRES           0.366798 Hz
AQ              1.3631488 sec
RG                 205.37
DW                 20.800 usec
DE                   6.50 usec
TE                  299.0 K
D1             2.00000000 sec
D11            0.03000000 sec
TD0                     1

======== CHANNEL f1 ========
SFO1          100.6278588 MHz
NUC1                  13C
P1                  10.00 usec
PLW1          47.00000000 W

======== CHANNEL f2 ========
SFO2          400.1516006 MHz
NUC2                   1H
CPDPRG[2          waltz16
PCPD2               90.00 usec
PLW2          18.00000000 W
PLW12          0.34722000 W
PLW13          0.28125000 W

F2 - Processing parameters
SI                  32768
SF            100.6177975 MHz
WDW                    EM
SSB      0
LB                   1.00 Hz
GB       0
PC                   1.40
```

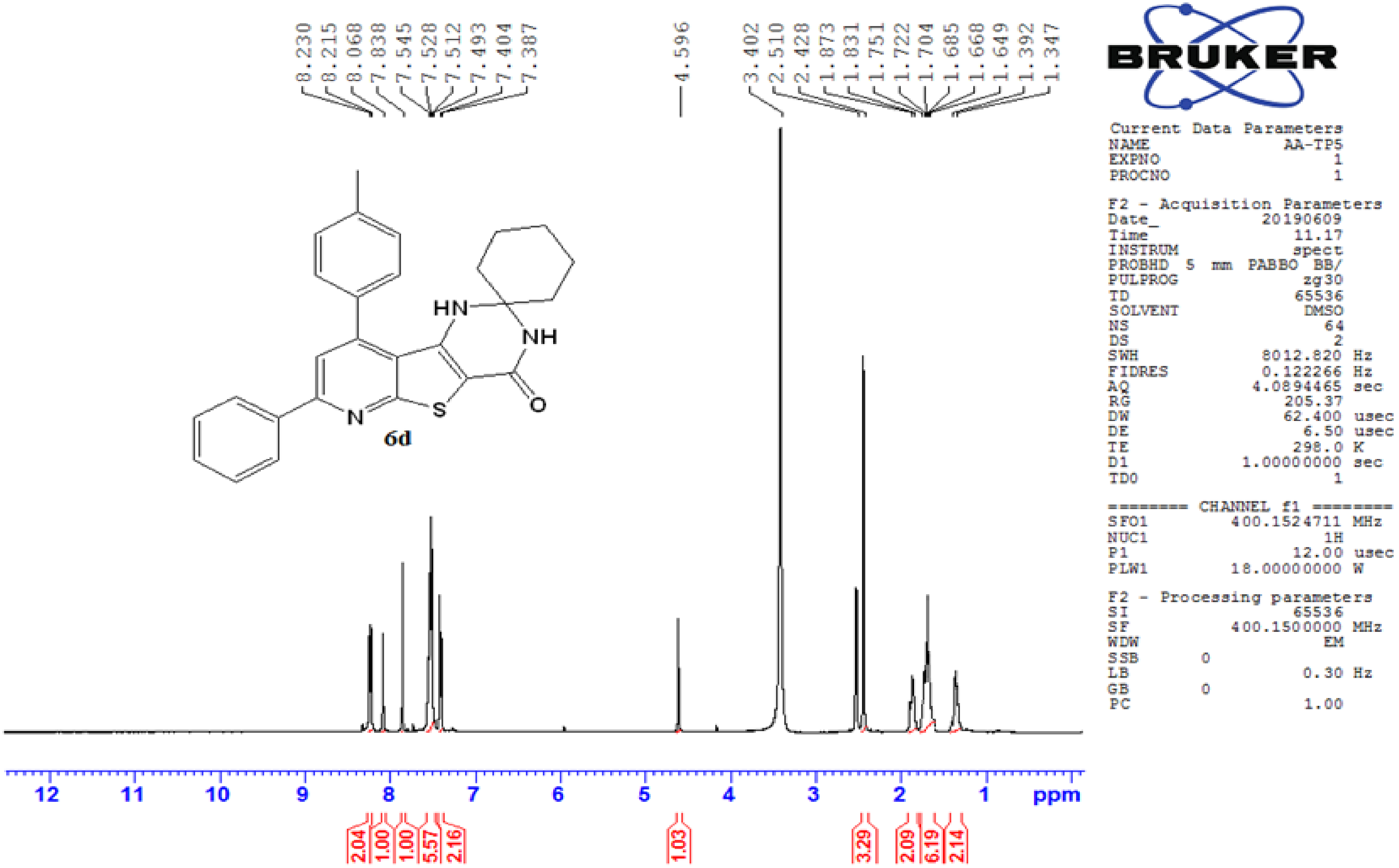
8.230
8.215
8.068
7.838
7.545
7.528
7.512
7.493
7.404
7.387
4.596
3.402
2.510
2.428
1.873
1.831
1.751
1.722
1.704
1.685
1.668
1.649
1.392
1.347
HN
NH
N
S
O
6d
BRUKER
Current Data Parameters
NAME AA-TP5
EXPNO 1
PROCNO 1
F2 - Acquisition Parameters
Date_ 20190609
Time 11.17
INSTRUM spect
PROBHD 5 mm PABBO BB/
PULPROG zg30
TD 65536
SOLVENT DMSO
NS 64
DS 2
SWH 8012.820 Hz
FIDRES 0.122266 Hz
AQ 4.0894465 sec
RG 205.37
DW 62.400 usec
DE 6.50 usec
TE 298.0 K
D1 1.00000000 sec
TD0 1
======== CHANNEL f1 ========
SFO1 400.1524711 MHz
NUC1 1H
P1 12.00 usec
PLW1 18.00000000 W
F2 - Processing parameters
SI 65536
SF 400.1500000 MHz
WDW EM
SSB 0
LB 0.30 Hz
GB 0
PC 1.00
12
11
10
9
8
7
6
5
4
3
2
1
ppm
2.04
1.00
1.00
5.57
2.16
1.03
3.29
2.09
6.19
2.14

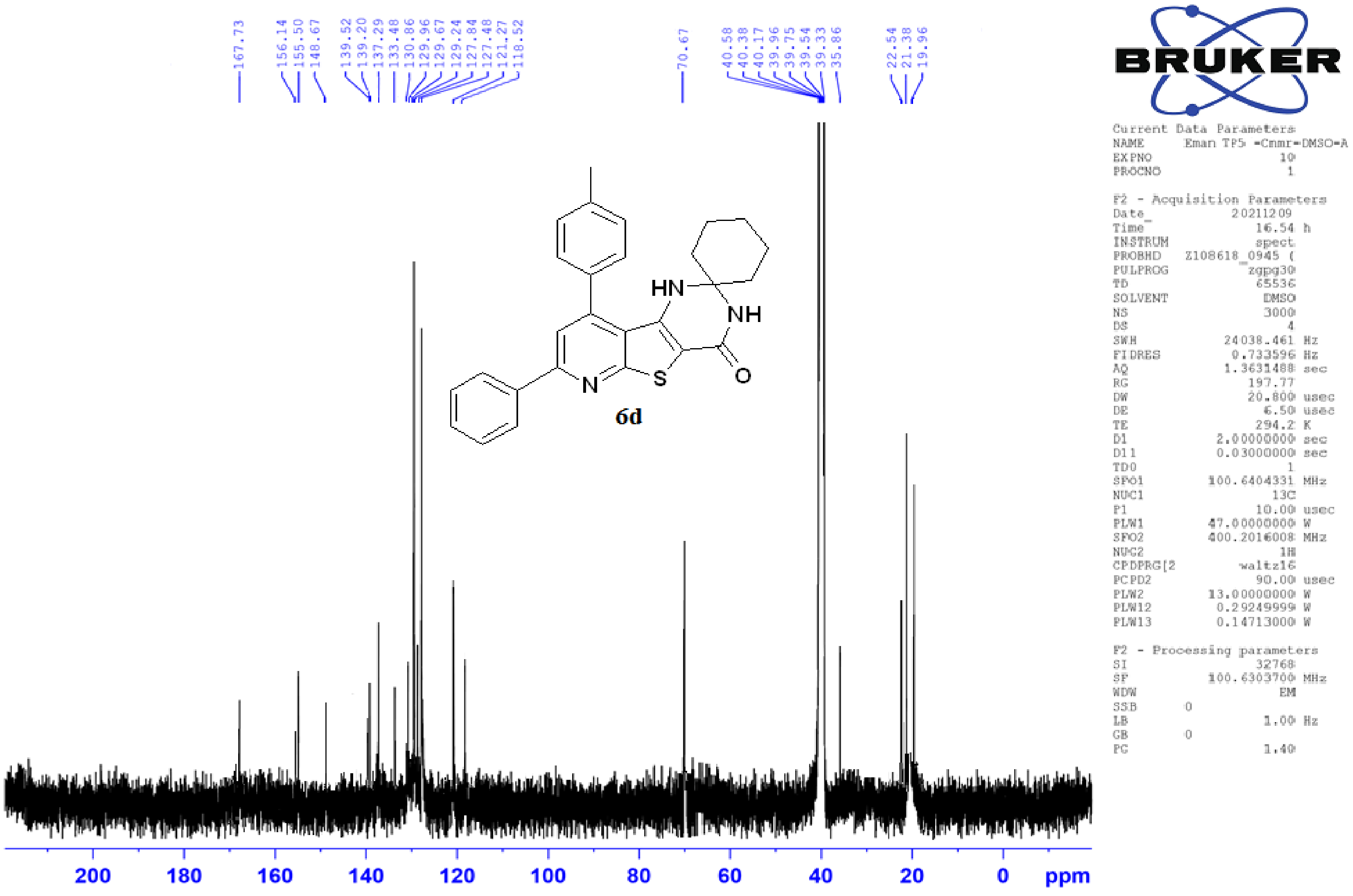
167.73
156.14
155.50
148.67
139.52
139.20
137.29
133.48
130.86
129.96
129.67
129.24
127.84
127.48
121.27
118.52
70.67
40.58
40.38
40.17
39.96
39.75
39.54
39.33
35.86
22.54
21.38
19.96
HN
NH
N
S
O
6d
200
180
160
140
120
100
80
60
40
20
0
ppm
BRUKER
Current Data Parameters
NAME Eman TP5 -Cnmr-DMSO-A
EXPNO 10
PROCNO 1
F2 - Acquisition Parameters
Date_ 20211209
Time 16.54 h
INSTRUM spect
PROBHD Z108618_0945 (
PULPROG zgpg30
TD 65536
SOLVENT DMSO
NS 3000
DS 4
SWH 24038.461 Hz
FIDRES 0.733596 Hz
AQ 1.3631488 sec
RG 197.77
DW 20.800 usec
DE 6.50 usec
TE 294.2 K
D1 2.00000000 sec
D11 0.03000000 sec
TD0 1
SFO1 100.6404331 MHz
NUC1 13C
P1 10.00 usec
PLW1 47.00000000 W
SFO2 400.2016008 MHz
NUC2 1H
CPDPRG[2 waltz16
PCPD2 90.00 usec
PLW2 13.00000000 W
PLW12 0.29249999 W
PLW13 0.14713000 W
F2 - Processing parameters
SI 32768
SF 100.6303700 MHz
WDW EM
SSB 0
LB 1.00 Hz
GB 0
PC 1.40

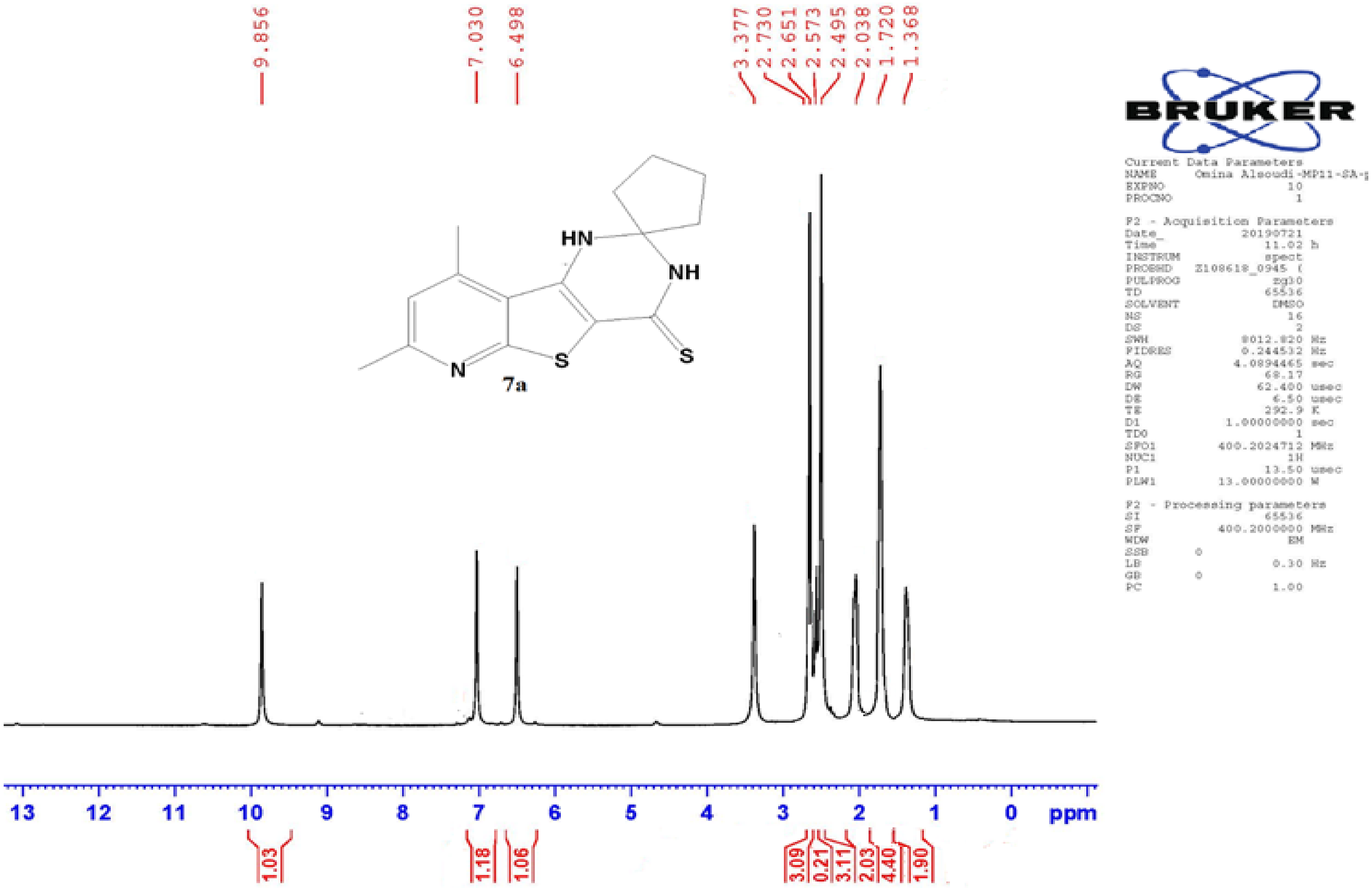
9.856
7.030
6.498
3.377
2.730
2.651
2.573
2.495
2.038
1.720
1.368
HN
NH
S
N
S
7a
Current Data Parameters
NAME Omina Alsoudi-MP11-SA-
EXPNO 10
PROCNO 1
F2 - Acquisition Parameters
Date_ 20190721
Time 11.02 h
INSTRUM spect
PROBHD Z108618_0945 (
PULPROG zg30
TD 65536
SOLVENT DMSO
NS 16
DS 2
SWH 8012.820 Hz
FIDRES 0.244532 Hz
AQ 4.0894465 sec
RG 68.17
DW 62.400 usec
DE 6.50 usec
TE 292.9 K
D1 1.00000000 sec
TD0 1
SFO1 400.2024712 MHz
NUC1 1H
P1 13.50 usec
PLW1 13.00000000 W
F2 - Processing parameters
SI 65536
SF 400.2000000 MHz
WDW EM
SSB 0
LB 0.30 Hz
GB 0
PC 1.00
13
12
11
10
9
8
7
6
5
4
3
2
1
0
ppm
1.03
1.18
1.06
3.09
0.21
3.11
2.03
4.40
1.90

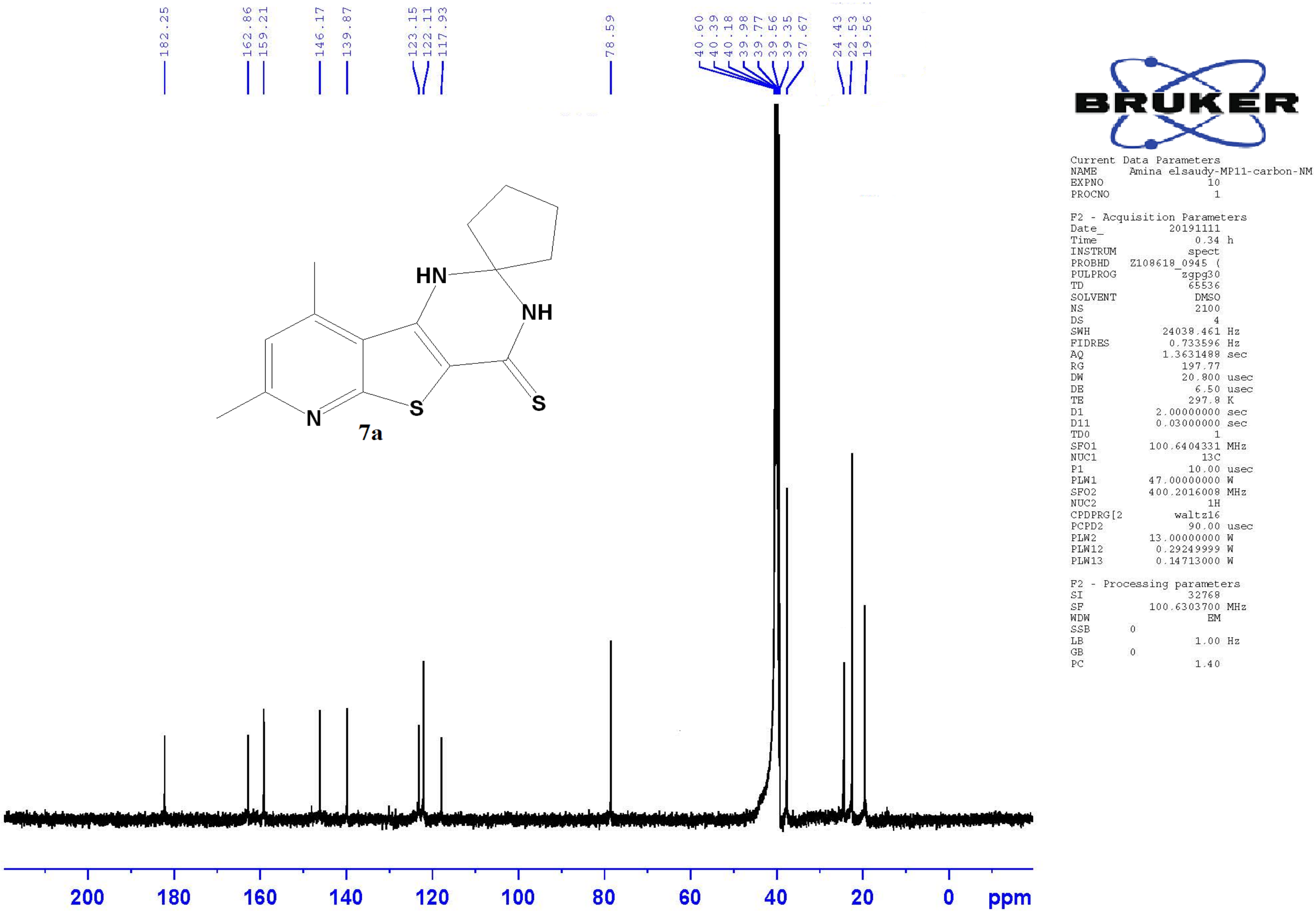
182.25
162.86
159.21
146.17
139.87
123.15
122.11
117.93
78.59
40.60
40.39
40.18
39.98
39.77
39.56
39.35
37.67
24.43
22.53
19.56
HN
NH
N
S
S
7a
BRUKER
Current Data Parameters
NAME Amina elsaudy-MP11-carbon-NM
EXPNO 10
PROCNO 1
F2 - Acquisition Parameters
Date_ 20191111
Time 0.34 h
INSTRUM spect
PROBHD Z108618_0945 (
PULPROG zgpg30
TD 65536
SOLVENT DMSO
NS 2100
DS 4
SWH 24038.461 Hz
FIDRES 0.733596 Hz
AQ 1.3631488 sec
RG 197.77
DW 20.800 usec
DE 6.50 usec
TE 297.8 K
D1 2.00000000 sec
D11 0.03000000 sec
TD0 1
SFO1 100.6404331 MHz
NUC1 13C
P1 10.00 usec
PLW1 47.00000000 W
SFO2 400.2016008 MHz
NUC2 1H
CPDPRG[2 waltz16
PCPD2 90.00 usec
PLW2 13.00000000 W
PLW12 0.29249999 W
PLW13 0.14713000 W
F2 - Processing parameters
SI 32768
SF 100.6303700 MHz
WDW EM
SSB 0
LB 1.00 Hz
GB 0
PC 1.40
200
180
160
140
120
100
80
60
40
20
0
ppm

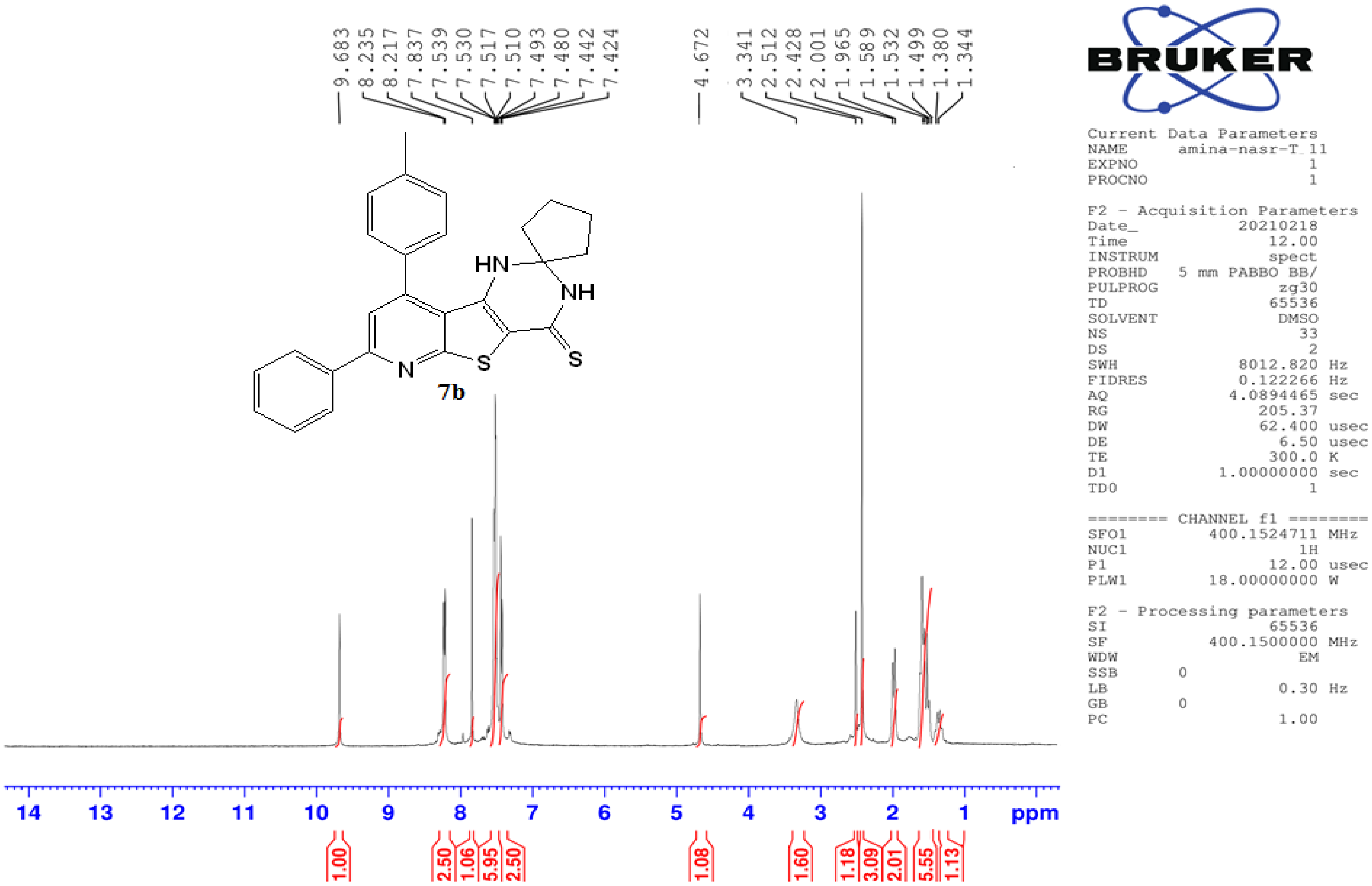
9.683 8.235 8.217 7.837 7.539 7.530 7.517 7.510 7.493 7.480 7.442 7.424
4.672
3.341 2.512 2.428 2.001 1.965 1.589 1.532 1.499 1.380 1.344
HN
NH
N
S
S
7b
14 13 12 11 10 9 8 7 6 5 4 3 2 1 ppm
1.00 2.50 1.06 5.95 2.50 1.08 1.60 1.18 3.09 2.01 5.55 1.13
BRUKER
Current Data Parameters
NAME amina-nasr-T 11
EXPNO 1
PROCNO 1
F2 - Acquisition Parameters
Date_ 20210218
Time 12.00
INSTRUM spect
PROBHD 5 mm PABBO BB/
PULPROG zg30
TD 65536
SOLVENT DMSO
NS 33
DS 2
SWH 8012.820 Hz
FIDRES 0.122266 Hz
AQ 4.0894465 sec
RG 205.37
DW 62.400 usec
DE 6.50 usec
TE 300.0 K
D1 1.00000000 sec
TD0 1
======== CHANNEL f1 ========
SFO1 400.1524711 MHz
NUC1 1H
P1 12.00 usec
PLW1 18.00000000 W
F2 - Processing parameters
SI 65536
SF 400.1500000 MHz
WDW EM
SSB 0
LB 0.30 Hz
GB 0
PC 1.00

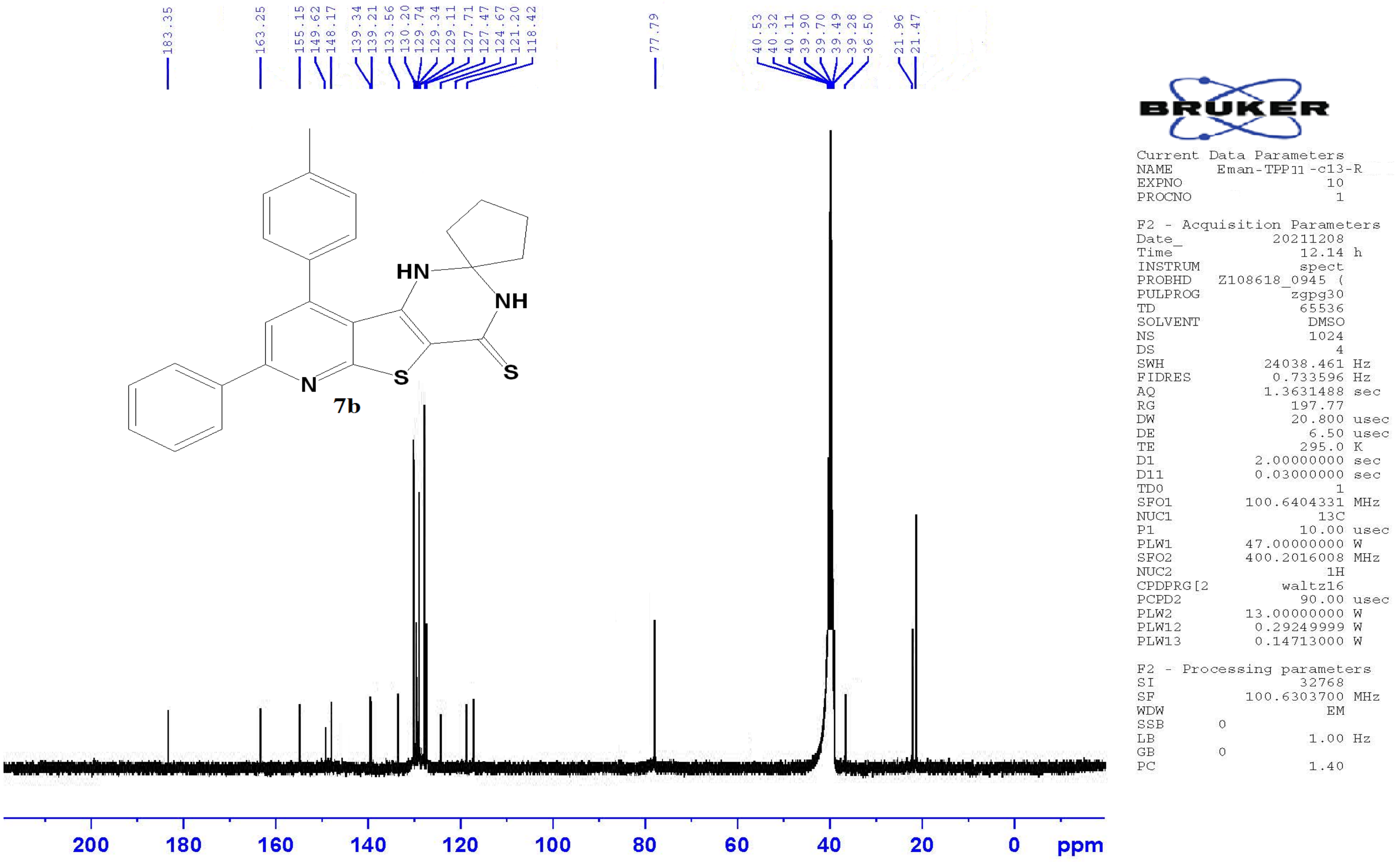
183.35
163.25
155.15
149.62
148.17
139.34
139.21
133.56
130.20
129.74
129.34
129.11
127.71
127.47
124.67
121.20
118.42
77.79
40.53
40.32
40.11
39.90
39.70
39.49
39.28
36.50
21.96
21.47
HN
NH
N
S
S
7b
BRUKER
Current Data Parameters
NAME Eman-TPP11-c13-R
EXPNO 10
PROCNO 1
F2 - Acquisition Parameters
Date_ 20211208
Time 12.14 h
INSTRUM spect
PROBHD Z108618_0945 (
PULPROG zgpg30
TD 65536
SOLVENT DMSO
NS 1024
DS 4
SWH 24038.461 Hz
FIDRES 0.733596 Hz
AQ 1.3631488 sec
RG 197.77
DW 20.800 usec
DE 6.50 usec
TE 295.0 K
D1 2.00000000 sec
D11 0.03000000 sec
TD0 1
SFO1 100.6404331 MHz
NUC1 13C
P1 10.00 usec
PLW1 47.00000000 W
SFO2 400.2016008 MHz
NUC2 1H
CPDPRG[2 waltz16
PCPD2 90.00 usec
PLW2 13.00000000 W
PLW12 0.29249999 W
PLW13 0.14713000 W
F2 - Processing parameters
SI 32768
SF 100.6303700 MHz
WDW EM
SSB 0
LB 1.00 Hz
GB 0
PC 1.40
200
180
160
140
120
100
80
60
40
20
0
ppm

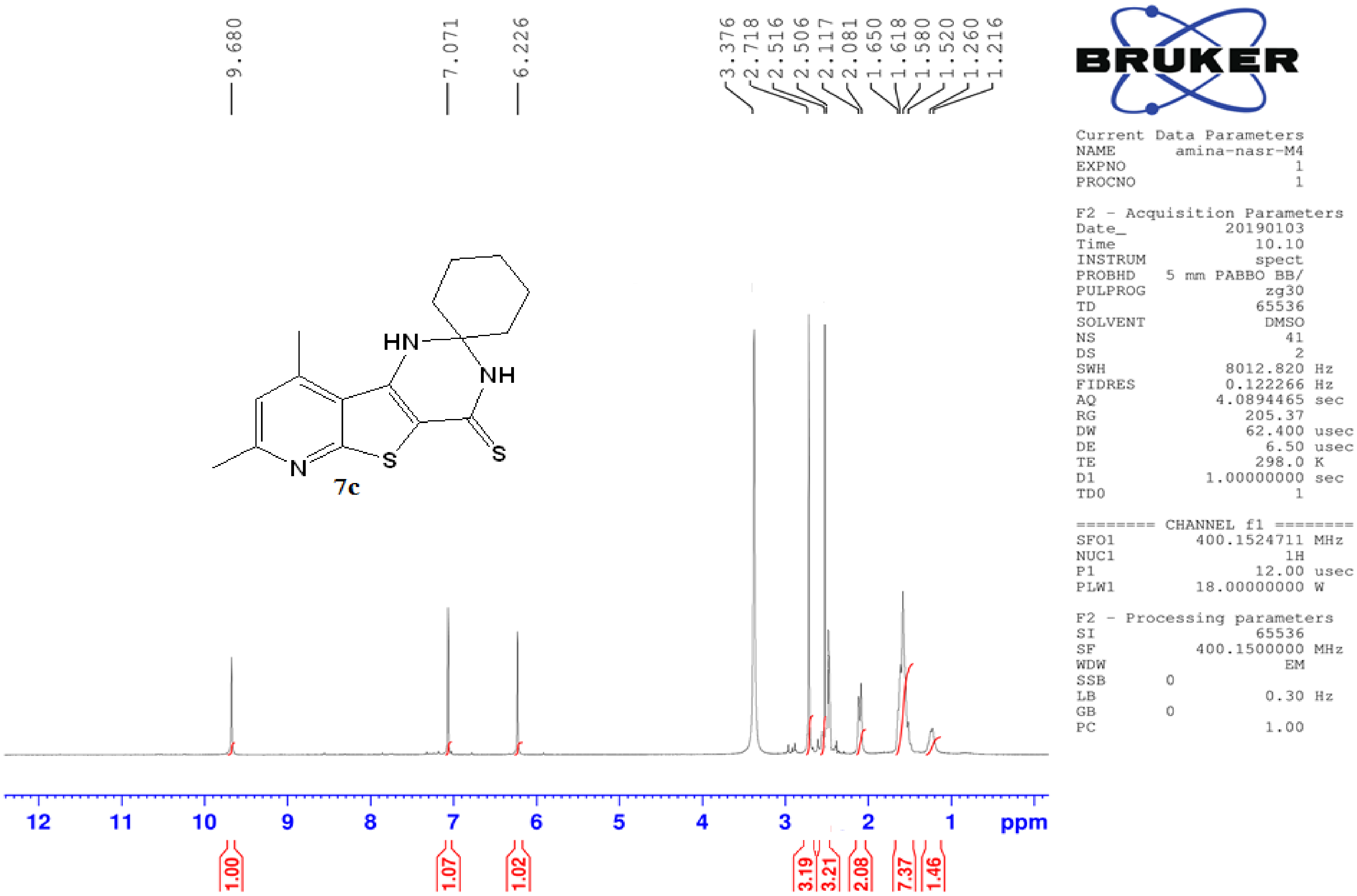
9.680
7.071
6.226
3.376
2.718
2.516
2.506
2.117
2.081
1.650
1.618
1.580
1.520
1.260
1.216
HN
NH
N
S
S
7c
12 11 10 9 8 7 6 5 4 3 2 1 ppm
1.00
1.07
1.02
3.19
3.21
2.08
7.37
1.46
BRUKER
Current Data Parameters
NAME amina-nasr-M4
EXPNO 1
PROCNO 1
F2 - Acquisition Parameters
Date_ 20190103
Time 10.10
INSTRUM spect
PROBHD 5 mm PABBO BB/
PULPROG zg30
TD 65536
SOLVENT DMSO
NS 41
DS 2
SWH 8012.820 Hz
FIDRES 0.122266 Hz
AQ 4.0894465 sec
RG 205.37
DW 62.400 usec
DE 6.50 usec
TE 298.0 K
D1 1.00000000 sec
TD0 1
======== CHANNEL f1 ========
SFO1 400.1524711 MHz
NUC1 1H
P1 12.00 usec
PLW1 18.00000000 W
F2 - Processing parameters
SI 65536
SF 400.1500000 MHz
WDW EM
SSB 0
LB 0.30 Hz
GB 0
PC 1.00

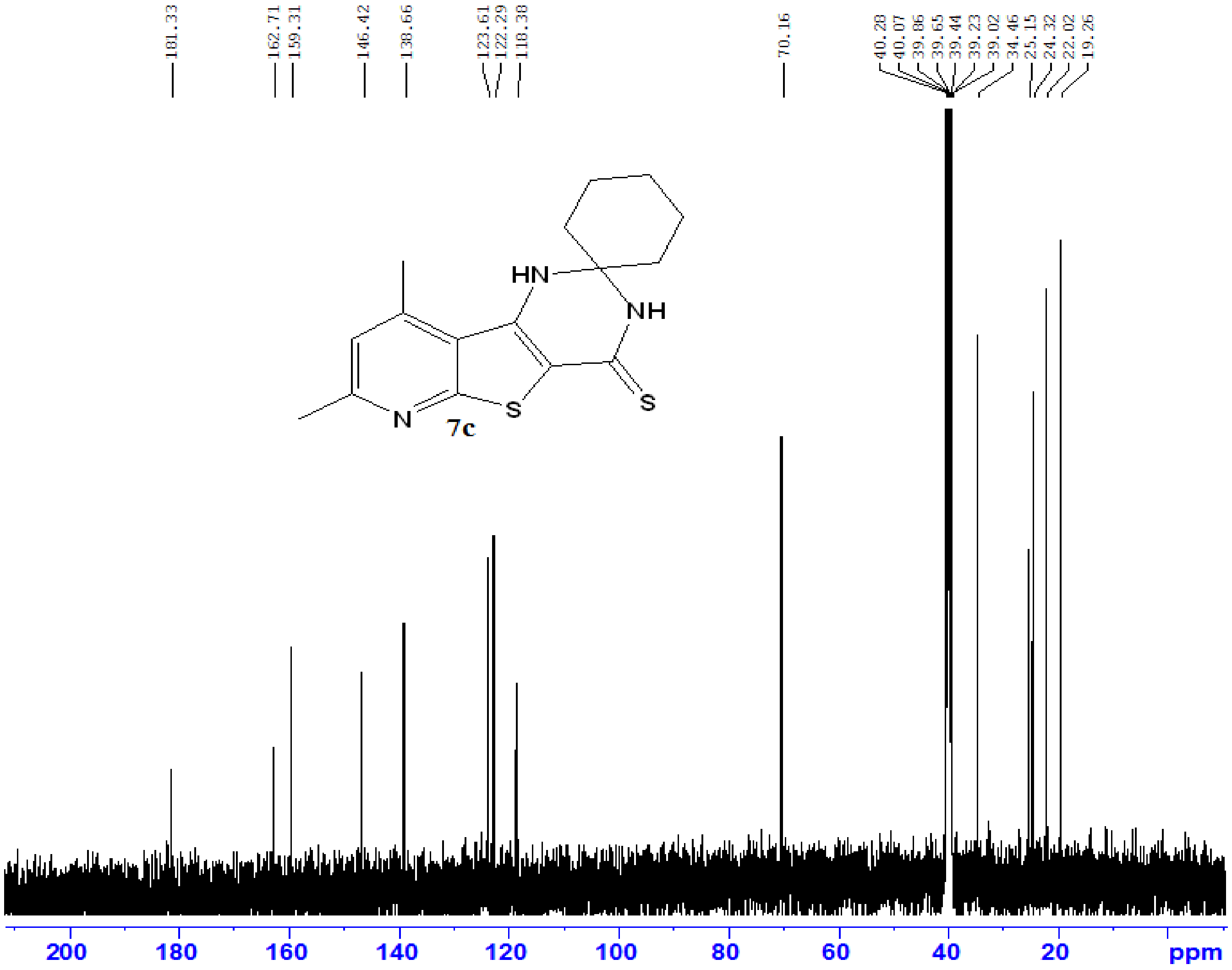


```
Current Data Parameters
NAME          amina-nasr-M4
EXPNO                     2
PROCNO                    1

F2 - Acquisition Parameters
Date_              20190113
Time                  12.29
INSTRUM               spect
PROBHD     5 mm PABBO BB/
PULPROG             zgpg30
TD                    65536
SOLVENT                DMSO
NS                     1092
DS                        4
SWH               24038.461 Hz
FIDRES             0.366798 Hz
AQ                1.3631488 sec
RG                   205.37
DW                   20.800 usec
DE                     6.50 usec
TE                    298.0 K
D1               2.00000000 sec
D11              0.03000000 sec
TD0                       1

======== CHANNEL f1 ========
SFO1            100.6278588 MHz
NUC1                    13C
P1                    10.00 usec
PLW1            47.00000000 W

======== CHANNEL f2 ========
SFO2            400.1516006 MHz
NUC2                     1H
CPDPRG[2            waltz16
PCPD2                 90.00 usec
PLW2            18.00000000 W
PLW12            0.34722000 W
PLW13            0.28125000 W

F2 - Processing parameters
SI                    32768
SF              100.6177975 MHz
WDW                      EM
SSB      0
LB                     1.00 Hz
GB       0
PC                     1.40
```

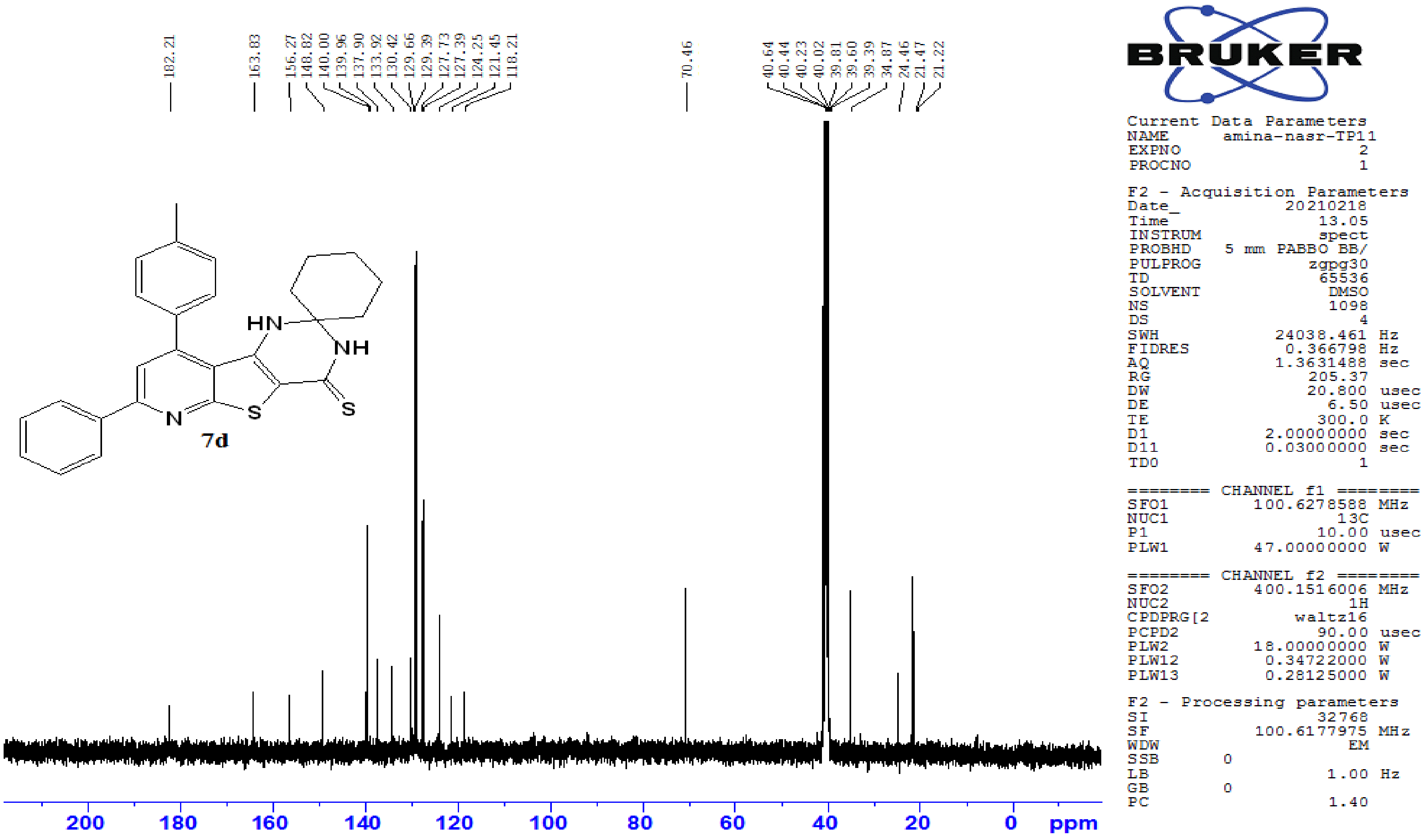
182.21
163.83
156.27
148.82
140.00
139.96
137.90
133.92
130.42
129.66
129.39
127.73
127.39
124.25
121.45
118.21
70.46
40.64
40.44
40.23
40.02
39.81
39.60
39.39
34.87
24.46
21.47
21.22
HN
NH
N
S
S
7d
200
180
160
140
120
100
80
60
40
20
0
ppm
BRUKER
Current Data Parameters
NAME amina-nasr-TP11
EXPNO 2
PROCNO 1
F2 - Acquisition Parameters
Date_ 20210218
Time 13.05
INSTRUM spect
PROBHD 5 mm PABBO BB/
PULPROG zgpg30
TD 65536
SOLVENT DMSO
NS 1098
DS 4
SWH 24038.461 Hz
FIDRES 0.366798 Hz
AQ 1.3631488 sec
RG 205.37
DW 20.800 usec
DE 6.50 usec
TE 300.0 K
D1 2.00000000 sec
D11 0.03000000 sec
TD0 1
======== CHANNEL f1 ========
SFO1 100.6278588 MHz
NUC1 13C
P1 10.00 usec
PLW1 47.00000000 W
======== CHANNEL f2 ========
SFO2 400.1516006 MHz
NUC2 1H
CPDPRG[2 waltz16
PCPD2 90.00 usec
PLW2 18.00000000 W
PLW12 0.34722000 W
PLW13 0.28125000 W
F2 - Processing parameters
SI 32768
SF 100.6177975 MHz
WDW EM
SSB 0
LB 1.00 Hz
GB 0
PC 1.40

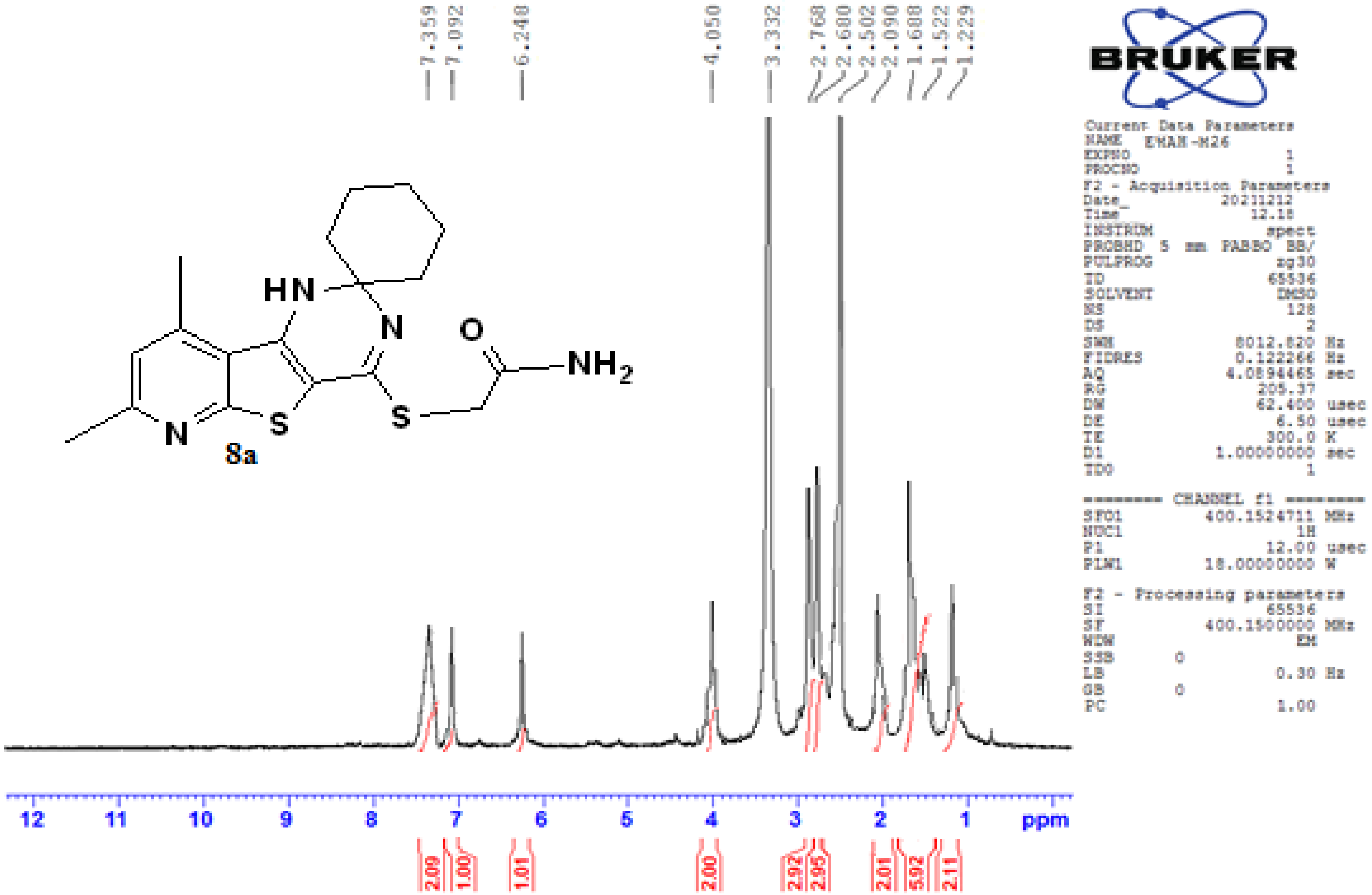
7.359
7.092
6.248
4.050
3.332
2.768
2.600
2.502
2.090
1.688
1.522
1.229
HN
N
O
NH2
N
S
S
8a
12 11 10 9 8 7 6 5 4 3 2 1 ppm
2.09
1.00
1.01
2.00
2.92
2.95
2.01
5.92
2.11
BRUKER
Current Data Parameters
NAME EHAN-H26
EXPNO 1
PROCNO 1
F2 - Acquisition Parameters
Date_ 20211212
Time 12.18
INSTRUM spect
PROBHD 5 mm PABBO BB/
PULPROG zg30
TD 65536
SOLVENT DMSO
NS 128
DS 2
SWH 8012.820 Hz
FIDRES 0.122266 Hz
AQ 4.0894465 sec
RG 205.37
DW 62.400 usec
DE 6.50 usec
TE 300.0 K
D1 1.00000000 sec
TD0 1
======== CHANNEL f1 ========
SFO1 400.1524711 MHz
NUC1 1H
P1 12.00 usec
PLW1 18.00000000 W
F2 - Processing parameters
SI 65536
SF 400.1500000 MHz
WDW EM
SSB 0
LB 0.30 Hz
GB 0
PC 1.00

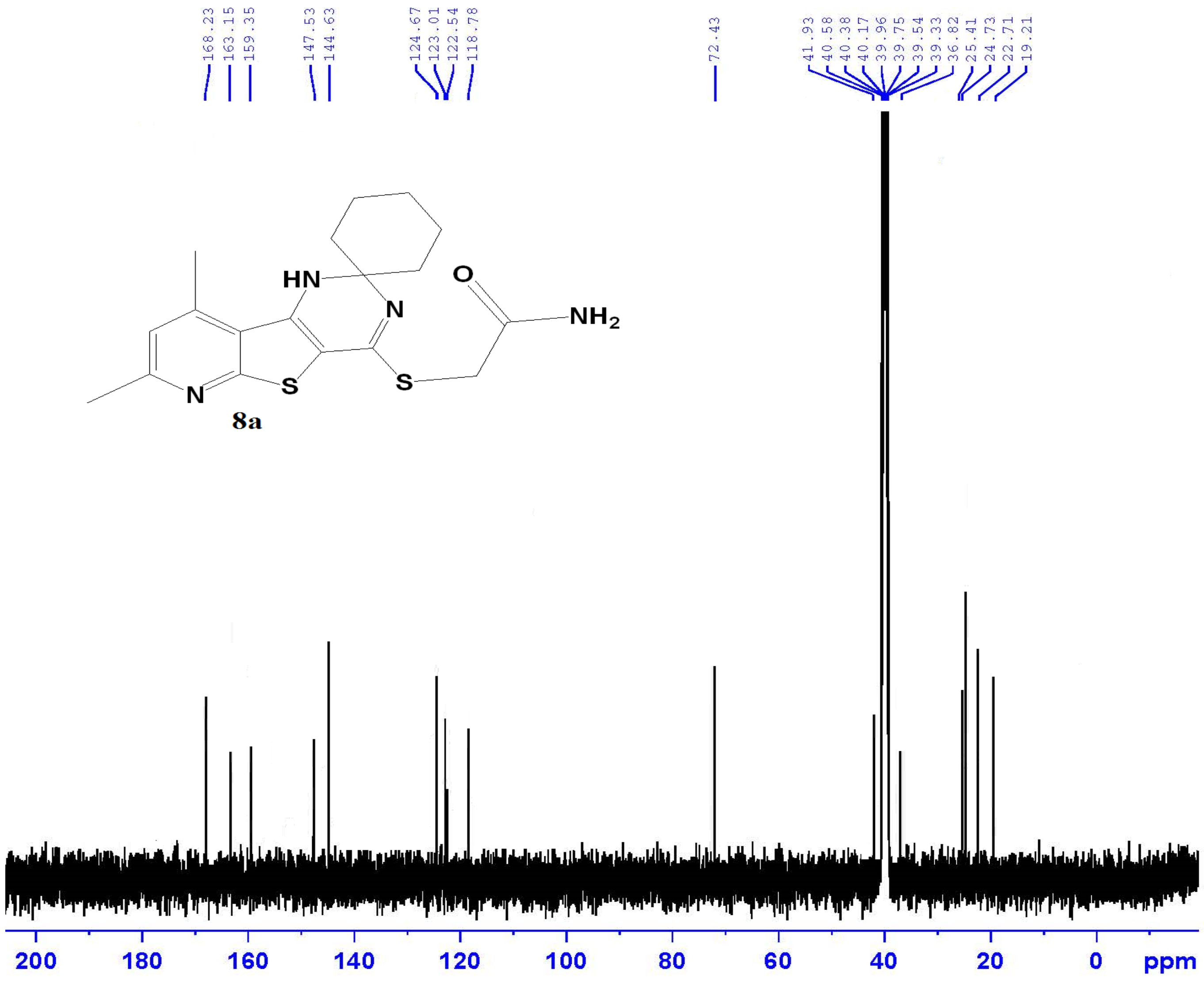

Current Data Parameters
NAME Eman -M 26
EXPNO 10
PROCNO 1

F2 - Acquisition Parameters
Date_ 20211209
Time 6.20 h
INSTRUM spect
PROBHD Z108618_0945 (
PULPROG zgpg30
TD 65536
SOLVENT DMSO
NS 1024
DS 4
SWH 24038.461 Hz
FIDRES 0.733596 Hz
AQ 1.3631488 sec
RG 197.77
DW 20.800 usec
DE 6.50 usec
TE 293.9 K
D1 2.00000000 sec
D11 0.03000000 sec
TD0 1
SFO1 100.6404331 MHz
NUC1 13C
P1 10.00 usec
PLW1 47.00000000 W
SFO2 400.2016008 MHz
NUC2 1H
CPDPRG[2 waltz16
PCPD2 90.00 usec
PLW2 13.00000000 W
PLW12 0.29249999 W
PLW13 0.14713000 W

F2 - Processing parameters
SI 32768
SF 100.6303700 MHz
WDW EM
SSB 0
LB 1.00 Hz
GB 0
PC 1.40

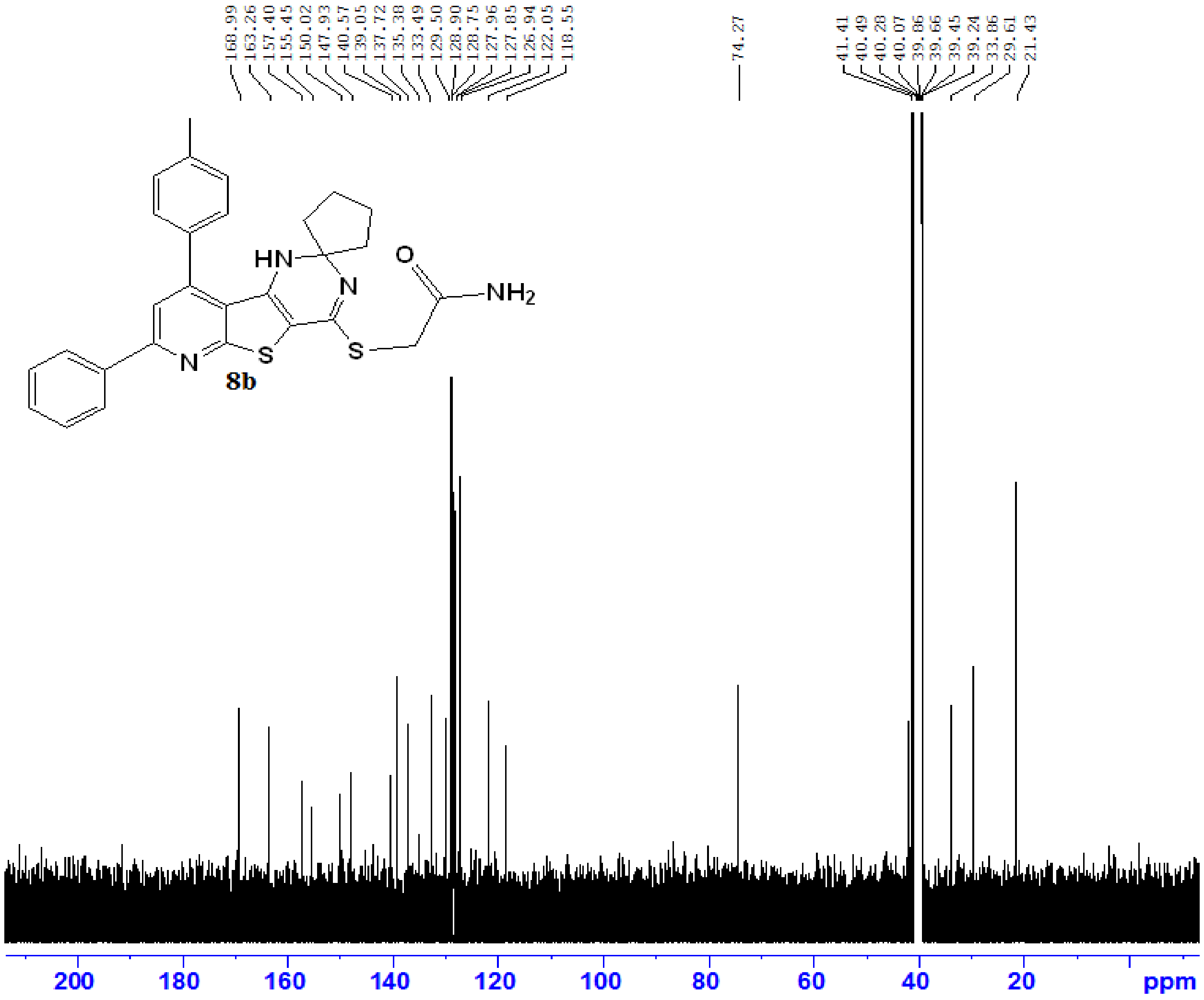

BRUKER

```
Current Data Parameters
NAME      amina-nasr-TPP23
EXPNO                    3
PROCNO                   1

F2 - Acquisition Parameters
Date_             20211018
Time                  8.11
INSTRUM              spect
PROBHD    5 mm PABBO BB/
PULPROG            zgpg30
TD                   65536
SOLVENT               DMSO
NS                   15295
DS                       4
SWH              24038.461 Hz
FIDRES            0.366798 Hz
AQ               1.3631488 sec
RG                  205.37
DW                  20.800 usec
DE                    6.50 usec
TE                   300.0 K
D1              2.00000000 sec
D11             0.03000000 sec
TD0                      1

======== CHANNEL f1 ========
SFO1          100.6278588 MHz
NUC1                  13C
P1                  10.00 usec
PLW1          47.00000000 W

======== CHANNEL f2 ========
SFO2          400.1516006 MHz
NUC2                   1H
CPDPRG[2             waltz16
PCPD2               90.00 usec
PLW2          18.00000000 W
PLW12          0.34722000 W
PLW13          0.28125000 W

F2 - Processing parameters
SI                  32768
SF            100.6177975 MHz
WDW                    EM
SSB       0
LB                   1.00 Hz
GB        0
PC                   1.40
```

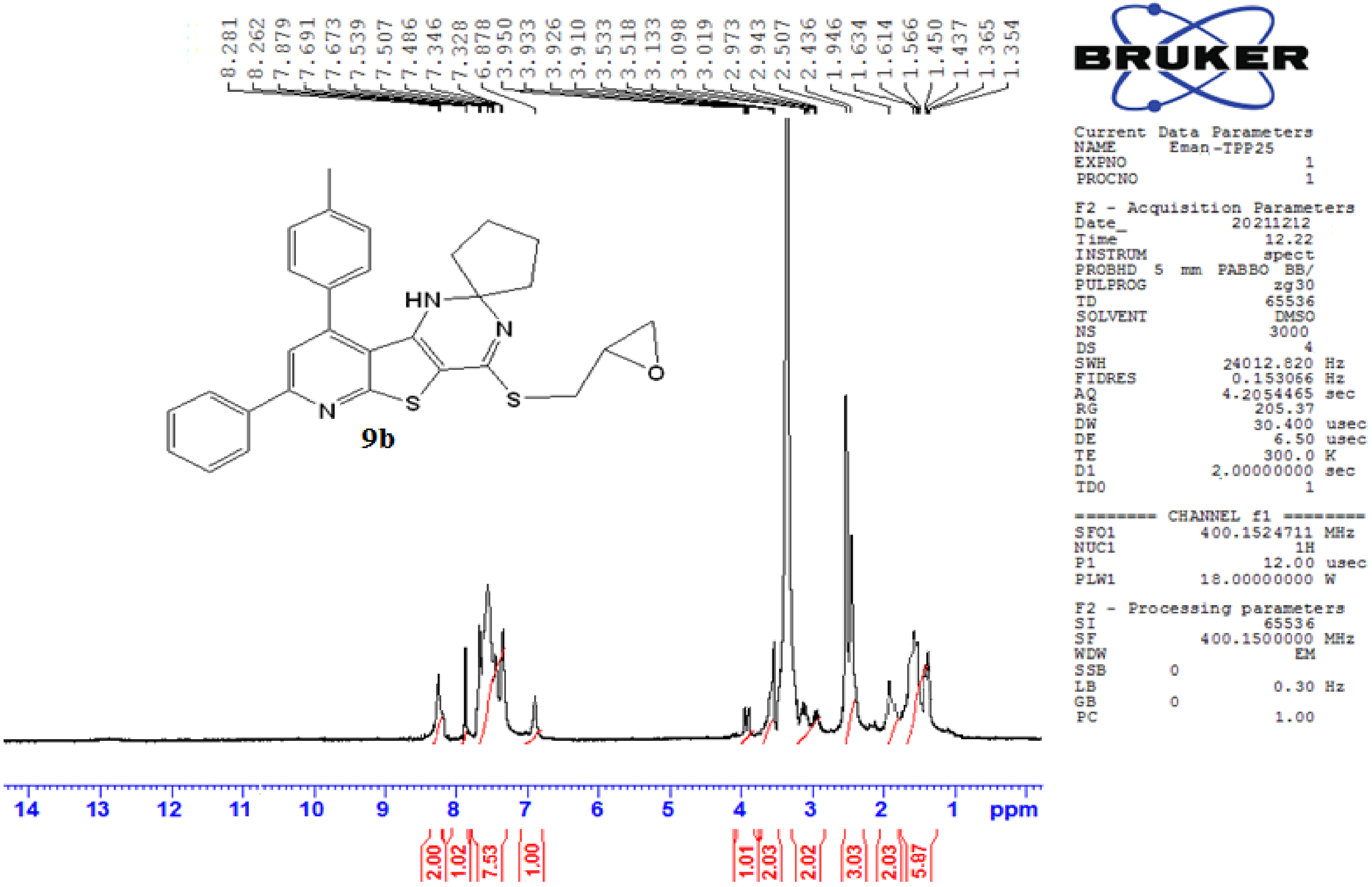
8.281
8.262
7.879
7.691
7.673
7.539
7.507
7.486
7.346
7.328
6.878
3.950
3.933
3.926
3.910
3.533
3.518
3.133
3.098
3.019
2.973
2.943
2.507
2.436
1.946
1.634
1.614
1.566
1.450
1.437
1.365
1.354
HN
N
N
S
S
O
9b
BRUKER
Current Data Parameters
NAME Eman-TPP25
EXPNO 1
PROCNO 1
F2 - Acquisition Parameters
Date_ 20211212
Time 12.22
INSTRUM spect
PROBHD 5 mm PABBO BB/
PULPROG zg30
TD 65536
SOLVENT DMSO
NS 3000
DS 4
SWH 24012.820 Hz
FIDRES 0.153066 Hz
AQ 4.2054465 sec
RG 205.37
DW 30.400 usec
DE 6.50 usec
TE 300.0 K
D1 2.00000000 sec
TD0 1
======== CHANNEL f1 ========
SFO1 400.1524711 MHz
NUC1 1H
P1 12.00 usec
PLW1 18.00000000 W
F2 - Processing parameters
SI 65536
SF 400.1500000 MHz
WDW EM
SSB 0
LB 0.30 Hz
GB 0
PC 1.00
14 13 12 11 10 9 8 7 6 5 4 3 2 1 ppm
2.00
1.02
7.53
1.00
1.01
2.03
2.02
3.03
2.03
5.87

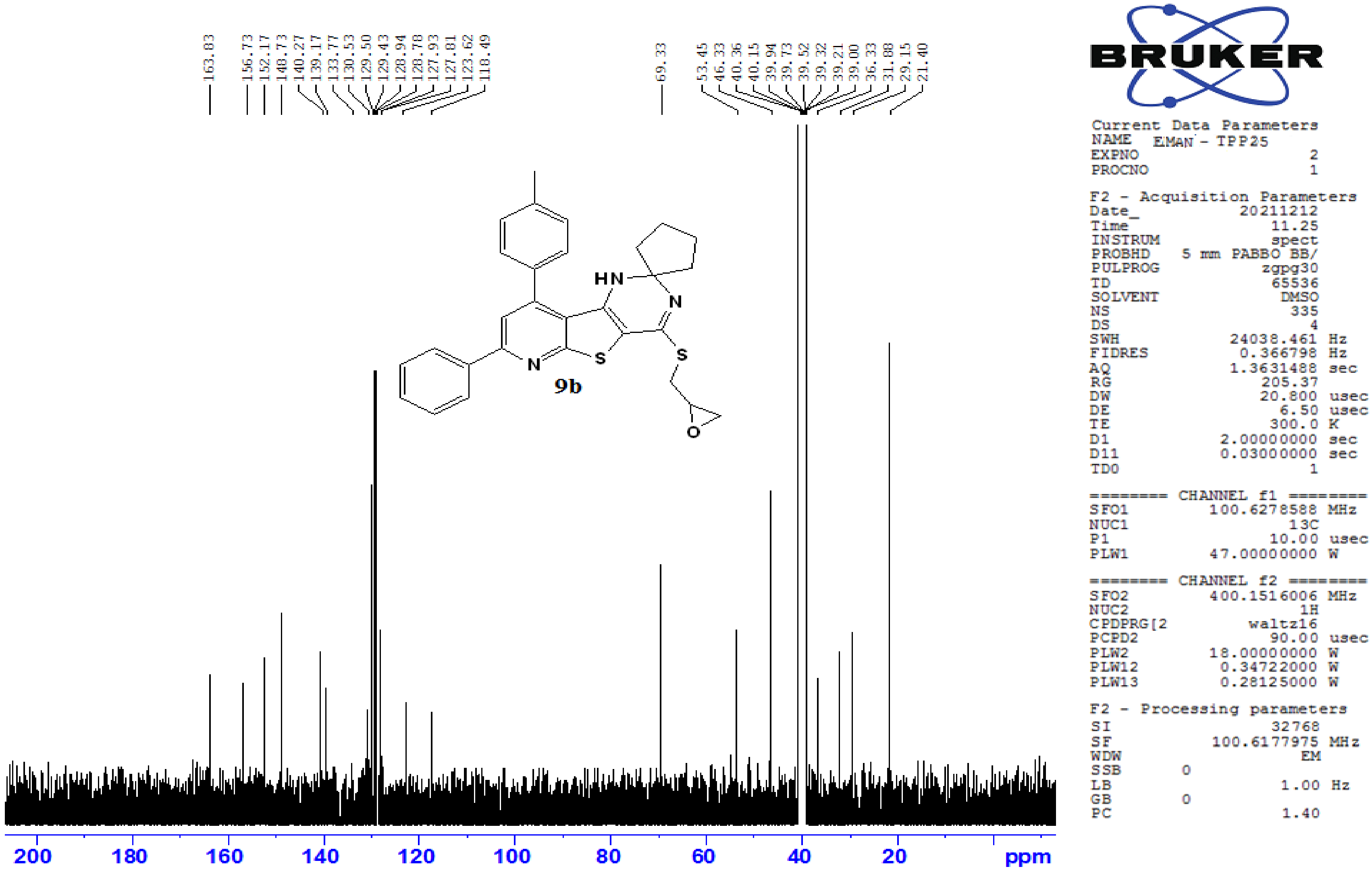
163.83
156.73
152.17
148.73
140.27
139.17
133.77
130.53
129.50
129.43
128.94
128.78
127.93
127.81
123.62
118.49
69.33
53.45
46.33
40.36
40.15
39.94
39.73
39.52
39.32
39.21
39.00
36.33
31.88
29.15
21.40
HN
N
S
N
S
O
9b
BRUKER
Current Data Parameters
NAME EMAN - TPP25
EXPNO 2
PROCNO 1
F2 - Acquisition Parameters
Date_ 20211212
Time 11.25
INSTRUM spect
PROBHD 5 mm PABBO BB/
PULPROG zgpg30
TD 65536
SOLVENT DMSO
NS 335
DS 4
SWH 24038.461 Hz
FIDRES 0.366798 Hz
AQ 1.3631488 sec
RG 205.37
DW 20.800 usec
DE 6.50 usec
TE 300.0 K
D1 2.00000000 sec
D11 0.03000000 sec
TD0 1
======== CHANNEL f1 ========
SFO1 100.6278588 MHz
NUC1 13C
P1 10.00 usec
PLW1 47.00000000 W
======== CHANNEL f2 ========
SFO2 400.1516006 MHz
NUC2 1H
CPDPRG[2 waltz16
PCPD2 90.00 usec
PLW2 18.00000000 W
PLW12 0.34722000 W
PLW13 0.28125000 W
F2 - Processing parameters
SI 32768
SF 100.6177975 MHz
WDW EM
SSB 0
LB 1.00 Hz
GB 0
PC 1.40
200
180
160
140
120
100
80
60
40
20
ppm

Minimum Inhibitory Concentration (MIC) Measurement

The minimum Inhibitory Concentration activity of the compounds was then evaluated using broth dilution method. Whereas, two-fold serial dilution at the concentrations (0.25, 0.5, 1, 2, 4, 8, 16, 32, 64, 128 µg/ml) was used to investigate the Minimum Inhibitory Concentration (MIC) values (expressed in µg/mL) for the target compounds and the reference drugs. The tubes were then inoculated with the test organisms, grown in their suitable broth for 24 h at 37 ˚C for bacteria, yeast and for 48 h at 30˚C for fungi activity (1×10^8 CFU/mL for bacteria and 1 $x10^6$ CFU/mL of yeast and fungi), each 2 mL received 0.1 mL of the above inoculums Positive controls were prepared separately for either bacteria, yeast or fungi with respective organisms in the same culture media without the target compounds. After incubation, the tube with lowest concentration of extract that shows no growth was taken as the MIC value for the respective organism.

In vitro anticancer screening

The cell lines were purchased from the American Type Culture collection as follows: breast carcinoma cell line (MCF-7) and the liver carcinoma cell line (HepG2). Cytotoxic activity screening was performed using MTT assay at Regional Center for Mycology and Biotechnology, Al- Azhar University. Exponentially, cells were placed in 10^4 cells/ well for 24 h, and then add fresh medium which containing different concentration of the tested sample. Serial two-fold dilutions of the tested sample were added using a multichannel pipette. Moreover, all cells were cultivated at 37 °C, 5% CO_2 and 95% humidity. Also, incubation of control cells occurred at 37 °C. However, after incubation for 24 h different concentrations of sample (50, 25, 12.5, 6.25, 3.125, 1.56 and 0 µg L^{-1}) were added and continued the incubation for 48 h, then, add the crystal violet solution 1% to each well for 0.5 h to examine viable cells. Rinse the wells using water until no stain. After that, add 30% glacial acetic acid to all wells with shaking plates on Microplate reader (TECAN, Inc.) to measure the absorbance, using a test wavelength of 490 nm. Besides, compare the treated samples with the control cell. The cytotoxicity was estimated by IC_{50} (the concentration that inhibits 50% of growth of cancer cell) in µM for the tested compounds and the reference drugs doxorubicin and cisplatin.

In vitro EGFR kinase assay

EGFR kinase inhibitory assay were performed for the target compounds **3b**, **4a**, **5a**, **6b**, **8b** and **9b** with erlotinib as a reference inhibitor, by using the EGFR kinase assay kit (Cat. # 40321). The assay Kit is designed to measure EGFR Kinase activity for screening applications using Kinase-Glo® MAX as a detection reagent using Kinase-Glo® MAX as a detection reagent. Thaw 5x Kinase Buffer 1: ATP and PTK substrate Poly (Glu:Tyr 4:1) (10 mg/ml) kinase was provided. Then the master mixture was prepared: N wells x (6 µl 5x Kinase Buffer 1 + 1 µl ATP (500 µM) + 1 µl PTK substrate Poly (Glu:Tyr 4:1) (10 mg/ml)+ 17 µl water) and 25 µl of the mixture was added to every well. The tested compounds were dissolved in DMSO, then 5 µl of their serial two-fold dilutions at concentrations (150, 100, 50, 25, 12.5, 6.25, 3.125 and 1.56 nM) was added in each well labeled as “Test Inhibitor”. For the well labeled as “Positive Control", 5 µl of the same solution of the reference drug erlotinib and “Blank”, 5 µl of the same solution without inhibitor (Inhibitor buffer). (3 ml of 1x Kinase Buffer 1) was prepared by mixing 600 µl of 5x Kinase Buffer 1 with 2400 µl water. To the wells designated as "Blank", add 20 µl of 1x Kinase Buffer 1. The reaction initiated by adding 20 µl of diluted EGFR enzyme to the wells designated “Positive Control” and "Test Inhibitor Control". Incubate at 30°C for 40 minutes. Thaw Kinase-G lo Max reagent. After the 40 minute reaction, 50 µl of Kinase-Glo Max reagent was added to each well. The plate was covered with aluminum foil and incubate at room temperature for 15 minutes. Then the luminescence was measured using the microplate reader (Infinite M200 microplate reader, Tecan, Männedorf, Switzerland). All assays were performed in triplicate. The relative inhibition (%) of inhibitors were then calculated compared to the control with no inhibitor. Then the IC50 values and their standard deviation (SD) for the tested compounds and the reference drug were determined in (nM).

Molecular docking study

The molecular modeling studies were carried out using Molecular Operating Environment (MOE, 2019.0102) software. All minimizations were performed with MOE until an RMSD gradient of 0.1 kcal·mol^{-1}Å^{-1} with MMFF94x force field and the partial charges were automatically calculated. The X-ray crystallographic structure of Epidermal Growth Factor Receptor (**EGFR**) kinase domain complexed with a quinazoline inhibitor erlotinib (**ERL**) (**PDB ID: 1M17**) was downloaded from the protein data bank(**https://www.rcsb.org/structure/1M17**). For each co-crystallized enzyme; water molecules and ligands which are not involved in the binding were removed, the protein was prepared for the docking study using *Protonate 3D* protocol in MOE with default options. The co-crystalized ligand (**ERL**) was used to define the binding site for docking. Triangle Matcher placement method and London dG scoring function were used for docking

The Dock workflow begins with a prepared receptor and ligand loaded in MOE. The general algorithm is divided into stages,

1. Get the protein structure from protein data bank (PDB file), it's prepared through protonation and adding the required charges, water molecules not involved in interaction are removed.
2. Ligands' structures in suitable format were collected to form a structure database as MDB file and energy minimized to get the suitable conformers for docking procedure.
3. Use triangle matching placement method to generate a collection of poses from the pool of ligand conformations. Each of the generated poses is assigned a score.
4. Initial Scoring, poses generated by the placement methodology can be rescored using London dG method. Typically, scoring functions emphasize favorable hydrophobic, ionic and hydrogen bond contacts. For the Dock framework to work properly, all new scoring methods must assign low scores to good poses.
5. Refinement, poses resulting from the placement stage can be refined using either the explicit molecular mechanics forcefield method or the grid-based energetics method.
6. Final Scoring. The final poses can be rescored using one of several scoring schemes, we use London dG scoring function.
7. Generation of docking results database.
